# Cross-country differences in self-rated health: decomposing the influence of depressive symptoms

**DOI:** 10.1093/eurpub/ckag178

**Published:** 2026-09-29

**Authors:** Jacques Wels, Jane Maddock, Praveetha Patalay

**Affiliations:** Unit for Lifelong Health and Ageing, University College London, London, United Kingdom; Health & Society Research Unit, Université libre de Bruxelles, Brussels, Belgium; Division of Surgery and Interventional Science, University College London, London, United Kingdom; Unit for Lifelong Health and Ageing, University College London, London, United Kingdom; Centre for Longitudinal Studies (CLS), University College London, London, United Kingdom

## Abstract

Despite extensive research on self-rated health (SRH), the extent to which mental health contributes to SRH and whether this relationship varies across countries remains underexplored. This study quantifies the contribution of depressive symptoms to SRH and examines cross-national variation in this association. Using data from adults aged 55+ from 28 countries in the Survey of Health, Ageing, and Retirement in Europe (SHARE, wave 9, 2022) (*N* = 69 447), we applied four approaches: (i) country-specific pseudo-*R*² models assessing the contribution of depressive symptoms (EURO-D) to SRH, adjusting for sociodemographic factors, physical health, and functional limitations; (ii) partial correlation networks to visualize relationships among SRH, depressive symptoms, and physical health; (iii) Blinder–Oaxaca decompositions to quantify cross-country differences in SRH attributable to depressive symptoms; and (iv) stratification by gender. Depressive symptoms explained nearly as much variance in SRH as physical health. Partial correlation networks showed SRH as central to all health measures, with correlations between SRH and depressive symptoms ranging from 0.15 (Israel) to 0.37 (Romania). Decomposition analyses identified three patterns: countries with low prevalence but stronger SRH-depression associations (e.g. Denmark, Sweden), countries with higher prevalence but weaker SRH-depression associations (e.g. Estonia, Lithuania), and countries with both low prevalence and weak associations (e.g. Greece, Spain). Gender differences in SRH were not meaningfully explained by differences in depressive symptoms. In conclusion, this study finds that depressive symptoms independently contribute to SRH, but their influence varies cross-nationally, reflecting culturally shaped understandings of ‘health’.

## Introduction

Self-rated health (SRH) is a key indicator for comparing population health across Europe, but its validity depends on a consistent meaning that may vary culturally. Although a strong predictor of mortality [1–3], whether SRH uniformly captures mental health remains unexamined.

SRH goes beyond disease presence or mortality, it measures ‘something more—and something less—than objective medical ratings’ [4]. In this sense, SRH aligns with the World Health Organisation definition of health as ‘a state of complete physical, mental, and social wellbeing’ rather than merely the absence of disease [5]. Whether this broad, multidimensional understanding of health is consistently applied across countries remains unknown.

Interpretations of SRH vary across population subgroups and countries. Gender differences are consistent, with women reporting worse SRH than men; roughly 30% of this gap is explained by material conditions such as employment, education, or marital status [6]. Yet, the gap’s size and unexplained portion vary across countries. Age also affects the relationship between physical health, functional limitations and SRH. Younger people tend to rate their SRH more poorly than older adults if they had similar health conditions [7]. Socioeconomic factors such as education, employment, and wealth strongly also influence SRH differences [8, 9], with non-working respondents, for instance, more likely to report poor SRH for the same conditions [9]. Differences exist in how individuals experience and translate health conditions into SRH, and these differences extend across nations.

Cross-national comparisons reveal major variations in SRH, underlining its subjective component and reflecting differing illness perceptions and reporting behaviours [10]. Population characteristics (e.g. education, employment, age) and cultural understandings of health both help explain these differences. Such cultural variation may concern whether mental health is viewed as part of overall health. As Rose noted [11], SRH illustrates the distinction between individual susceptibility and population-level health distributions, raising questions about the validity of cross-country thresholds distinguishing ‘healthy’ from ‘unhealthy’ respondents.

Poor SRH is consistently linked to poor mental health outcomes [12], but mental health is rarely considered as a component of SRH itself. Recent analyses using the Survey of Health, Ageing and Retirement in Europe (SHARE) show that higher depressive symptoms (EURO-D scale) are associated with poorer perceived physical health and economic hardship [13]. This relationship also varies with age-being stronger among younger cohorts [14]. However, most studies treat mental health as a confounder rather than a component of SRH [15]. To date, no study has systematically examined whether mental health—specifically depressive symptoms—constitutes a core dimension of SRH, and whether cross-country differences in this respect explain variations in SRH validity.

To address this gap, this study uses cross-national data from the SHARE to answer the following research questions:

[RQ1] To what extent does self-rated health encompass depressive symptoms, and does this vary by country?

[RQ2] How do self-rated health, depressive symptoms and indicators of physical health, (activities of daily living and health conditions) correlate with each other, and does this vary by country?

[RQ3] Are these observed differences due to cross-country variations in population composition (age, income, gender, education), and what share of the SRH–depression association is independent of such composition?

[RQ4] To what extent do the relationships explored in RQ1–RQ3–i.e. the association between SRH and depressive symptoms and its cross-national variation–differ by gender?

## Methods

### The survey of health, ageing, and retirement in Europe

We used the last available wave–wave 9 (2022)—from the SHARE [16] including all respondents aged 55+ from 28 countries (see Table S1 for descriptive statistics and sample sizes).

### Self-rated health

The SRH question ‘How is your health in general’ has five response options: excellent, very good, good, fair, and poor. The SRH question was randomly placed within the SHARE questionnaire to mitigate ordering effects, which are known to influence self-assessments [17, 18]. Our main analyses used SRH as a numeric variable forming a continuum from good to poor health [19]. We replicated the main analyses using an interval regression modelling as a sensitivity check.

### Depressive symptoms

Depressive symptoms were measured using the EURO-D questionnaire [20]. EURO-D was originally developed to harmonize data on depression in late-life [21] and is among the most used survey measures of depressive symptoms in older population studies [13]. EURO-D assesses experience of depression symptoms over the past month with yes-no response options for each symptom. Symptoms include depressed mood, pessimism, suicidality, guilt, sleep, interest, irritability, appetite, fatigue, concentration, enjoyment, and tearfulness. Scores range from 0 to 12, higher scores indicating higher levels of depressive symptoms.

### Control variables

We used several control variables.

Health condition reflect whether the respondents reported at least one chronic health condition diagnosed by a medical doctor. Medical conditions include heart attack; high blood pressure or hypertension; high blood cholesterol; stroke or cerebral vascular disease; diabetes or high blood sugar; chronic lung disease; arthritis, including osteoarthritis, or rheumatism; cancer or malignant tumour; stomach or duodenal ulcer, peptic ulcer; Parkinson disease; cataracts; hip fracture or femoral fracture. The variable was dichotomized distinguishing those reporting at least one condition (=1) from those reporting no listed condition (=0). We selected this variable to address physical health issues because it reflects both the existence of specific health conditions that might translate into poorer SRH scores and the experience of daily living impairment that might also affect SRH.

We additionally included information on Activities of Daily Living (ADL), i.e. the difficulty performing ADL to maintain one’s person [22]. ADL scores information on tasks of dressing, bathing/showering, eating, cutting up food, walking across a room, and getting in or out of bed with values from zero to five, five indicating the highest level of difficulty. The variable was coded as binary, distinguishing those who report at least one issue with ADL. ADL is distinguished from health conditions in the sense that all conditions do not translate into ADL impairment and ADL could exist independently of health conditions [22] and is a strong confounder of wellbeing within elderly populations [23].

Demographic information included gender (ref.: male), age-group (55–65 or above), and marital status (married and living together with spouse or registered partnership (ref.), not married or divorced, and widowed). Information on respondents’ socioeconomic status include household incomes equivalized to reflect household composition (log) [24], the highest level of education (based on the International Standard Classification of Education—ISCED) distinguishing respondent with no formal or primary education (ISCED 0–1, respondents reporting secondary to university education (ISCED 2–6) (ref.) and those reporting another type of education not included in the ISCED nomenclature), housing tenure (owner [ref.], not owner) and employment status (retired (ref.), employed or self-employed, unemployed or inactive).

### Statistical analyses

#### Explained variance

To estimate the variance in SRH attributed to depressive symptoms, we estimated a series of weighted linear regression models separately for each country with SRH being the dependent variable. We used a stepwise modelling strategy to assess how the explanatory power of depressive symptoms changed with the inclusion of different sets of variables. Model 1 included sociodemographic and socioeconomic factors. Model 2 added health conditions and ADL. Model 3 further included the EURO-D score to assess its additional contribution to explaining SRH. This approach allowed us to isolate the role of depressive symptoms after accounting for other relevant factors and to compare their influence across countries. To estimate the proportion of variance explained by each model, we computed a weighted pseudo-*R*² based on the deviance of the fitted and null models. To assess the uncertainty around pseudo-*R*², we used non-parametric bootstrapping [25]. By comparing the pseudo-*R*² values across the three models, we were able to quantify how much depressive symptoms contributed to explaining SRH in each country.

#### Partial correlations networks

To examine the associations between SRH (numeric), ADL, the presence of at least one chronic health condition (binary), and the EURO-D scale (treated as numeric) we used partial correlation networks to generate separate plots for each selected country. In each network plot, nodes represent observed variables, while edges represent regularized partial correlations between pairs of variables, controlling for all others. To ensure sparsity, we applied Lasso regularization using the graphical Lasso method. The optimal tuning parameter was selected using the Extended Bayesian Information Criterion, as proposed by Epskamp and Fried [26]. In the final figures, edge thickness reflects the magnitude of the partial correlation, with thicker edges indicating stronger conditional associations. 95% confidence intervals (CI) are shown in the Supplementary Files.

##### Blinder–Oaxaca decomposition

To assess how much of the cross-country variation in SRH reflects population composition versus contextual differences in how characteristics shape health, we employed a Blinder–Oaxaca (BO) decomposition technique. Decomposition methods are widely used in the social sciences [27], and the BO approach—originally developed in economics—has recently been recognized for its potential to study health inequalities [28].

In this study, differences in mean SRH between each of the 28 countries in SHARE and a reference category (i.e. the sum of all countries weighted for each country to have identical sample size based on the original population weights) were expressed as the sum of two components. The explained component represents the part of the SRH gap due to differences in observable characteristics (such as depressive symptoms, physical health, and socioeconomic factors) weighted by the coefficients of the reference group. A positive explained term for depressive symptoms indicates that a share of the SRH difference is attributable to variations in symptom levels across countries. The unexplained component captures cross-national differences in how these characteristics are associated with SRH, reflecting unmeasured factors or contextual variation in the interpretation of health and wellbeing.


$$\Delta \bar{\mathrm{SRH}}={\bar{\mathrm{SRH}}}_{i}-{\bar{\mathrm{SRH}}}_{m}\;[a]$$

$$\mathrm{Explained}\;\mathrm{Component}={(\bar{\mathrm{eurod}}}_{i}-{\bar{\mathrm{eurod}}}_{m})*B_{m}+({\bar{I}}_{i}-{\bar{I}}_{m})*\gamma_{i}\;[b]$$

$$\mathrm{Unexplained}\;\mathrm{Component}={\bar{\mathrm{eurod}}}_{m}*(\beta_{i}-\beta_{m})+\;{\bar{I}}_{m}*(\gamma_{i}-\gamma_{m})\;[c]$$

Each country (i) was compared to a reference group (m) representing the weighted average SRH across all 28 countries. This approach avoids dependence on a single reference country and allows comparisons to a balanced benchmark (formula [a]). The explained and unexplained components correspond conceptually to formulas [b] and [c], with the total difference equal to their sum.

Because nonlinear models such as interval regressions do not allow additive decompositions by variable, main analyses were based on linear specifications, while interval regressions were used in sensitivity analyses to assess overall robustness.

### Weights

We used complete case analysis and included cross-sectional weights provided by SHARE.

### Stratification

We first conducted the analyses in the full sample and subsequently all analyses were stratified by gender to allow examination of gender differences.

### Sensitivity analysis

To address normal distributiveness issues and the ordinal nature of SRH, we also replicated the main analyses using an interval regression modelling. We additionally replicated the analyses using a pooled sample of waves 4 (2011), 5 (2013), and 6 (2015) (pre-COVID-19 waves) including 18 countries to address time differences. Each replicated model controlled for the wave of data collection when several waves were available by country.

### Software

Data cleaning, partial correlations and network analyses were conducted in R (version 1.79) and BO models in Stata (version 18.5) (specifically, the *oaxaca* and *nldecompose* commands).

## Results

### Descriptive results

Sample size and wave availability as well as descriptive statistics for SRH, depressive symptoms (EURO-D), health conditions, and limitations in ADL across European countries are shown in Table S1. Sample sizes by country range from *N* = 736 (Cyprus) to *N* = 4828 (Poland). On average, SRH was highest in Denmark (mean = 2.64) and Switzerland (2.79) and lowest in Estonia (3.73) and Latvia (3.75). Depression scores varied considerably, with the lowest levels in Greece (1.71) and Cyprus (1.96) and the highest in Lithuania (3.3) and Portugal (3.31). The prevalence of health conditions was generally high, particularly in Israel (0.78) and Greece (0.8), while Switzerland (0.55) and Slovakia (0.55) rated the lowest means. ADL limitations were least common in Slovakia (0.05) and Latvia (0.06) and most frequent in Israel (0.16) and Portugal (0.17).

### Explained variance

Figure 1 exhibits the explanatory power of three sequentially adjusted regression models for SRH across the 28 countries. The full results are shown in Table S2. The left panel displays pseudo-*R*² values with 95% CIs for: (i) a baseline socioeconomic model, (ii) an intermediate model incorporating physical health measures (chronic conditions and functional limitations), and (iii) a full model additionally including depressive symptoms. Countries are ordered by the *R*² values of the full model. The right panel illustrates the marginal gains in explanatory power achieved by adding physical health indicators and subsequently depressive symptoms, with a dashed zero-line reference indicating no improvement. A first observation is that, in all countries, both health conditions/ADL and EURO-D add explanatory power to the model. As illustrated in Fig. 1, adding physical health indicators and then depressive symptoms consistently increased the explained variance across all countries. The gains from depressive symptoms were substantial and independent of the gains from physical health.

**Figure 1:**
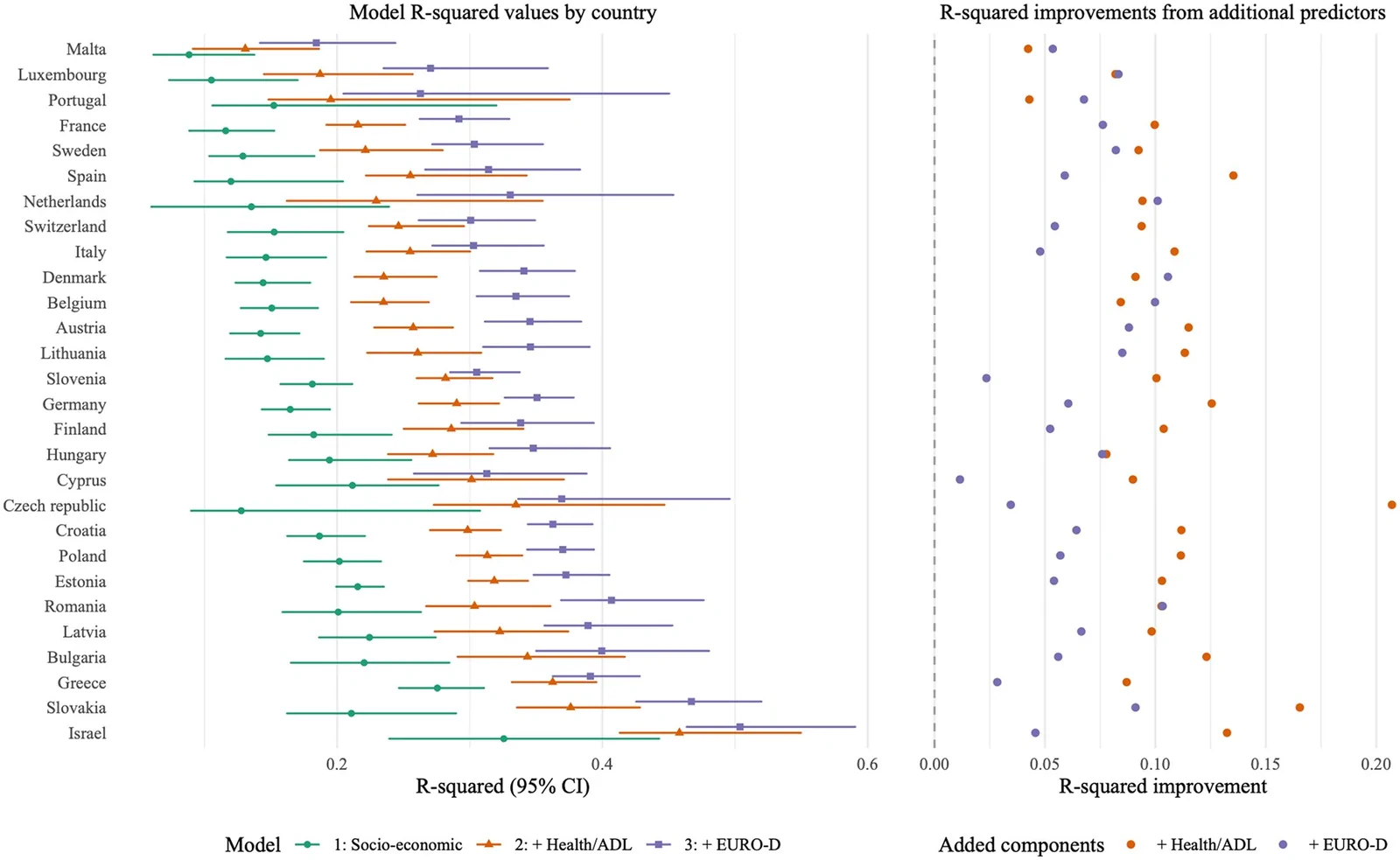
Country variations in pseudo-*R*-squared explained by socioeconomic variables, chronic conditions (health) and ADL and depressive symptoms (EUROD-D) (linear model).

We replicated the analyses using an interval regression and a linear model stratified by gender, with results in Tables S3 and S4 and Fig. S2.

### Partial correlations networks

Figure 2 shows the partial correlation networks by country. Full estimates and 95%CI are shown in Table S5. The graphs show the relative centrality of SRH among the other health-related variables. In all countries, SRH is independently associated with the presence of a health condition, ADL impairment, and depressive symptoms. However, the nature of such relationships vary from one country to another. Partial correlation networks (Fig. 2) confirmed that SRH was independently associated with health conditions, ADL, and depressive symptoms in all countries. The strength of the SRH-depression link varied notably, with partial correlations ranging from 0.15 (95%CI: −0.15; 0.27) (Israel) to 0.37 (95%CI: 0.31; 0.42) (Romania).

**Figure 2:**
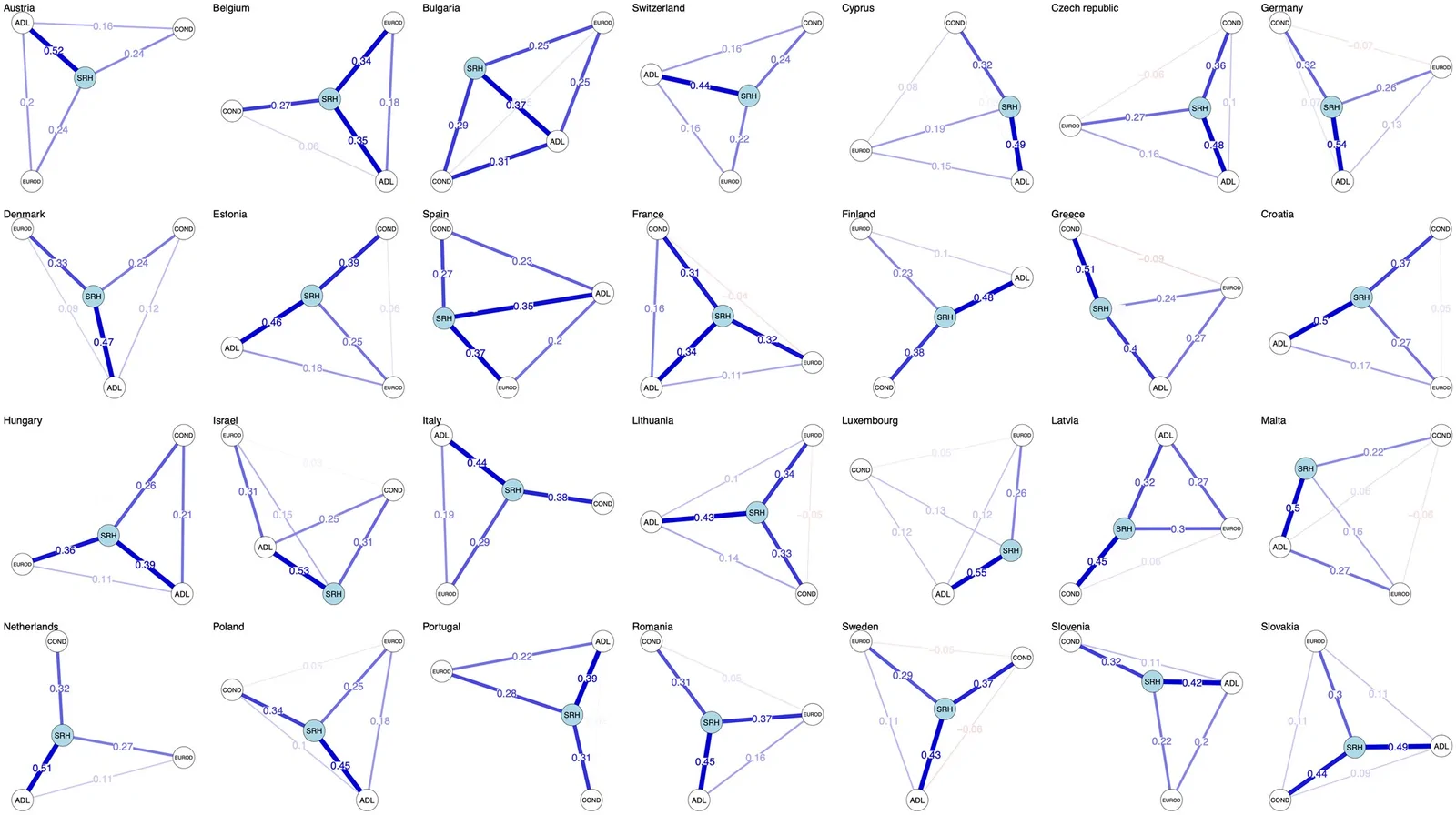
Partial correlations networks including depressive symptoms (EURO-D), ADL, chronic condition and SRH, by country. Complete cases. Legend: ADL, activities of daily living; COND, health condition(s); EUROD, depressive symptoms; SRH, self-rated health.

### BO decomposition

Main estimates from the BO decomposition are shown in Fig. 3 while full results are in Table S7.

**Figure 3:**
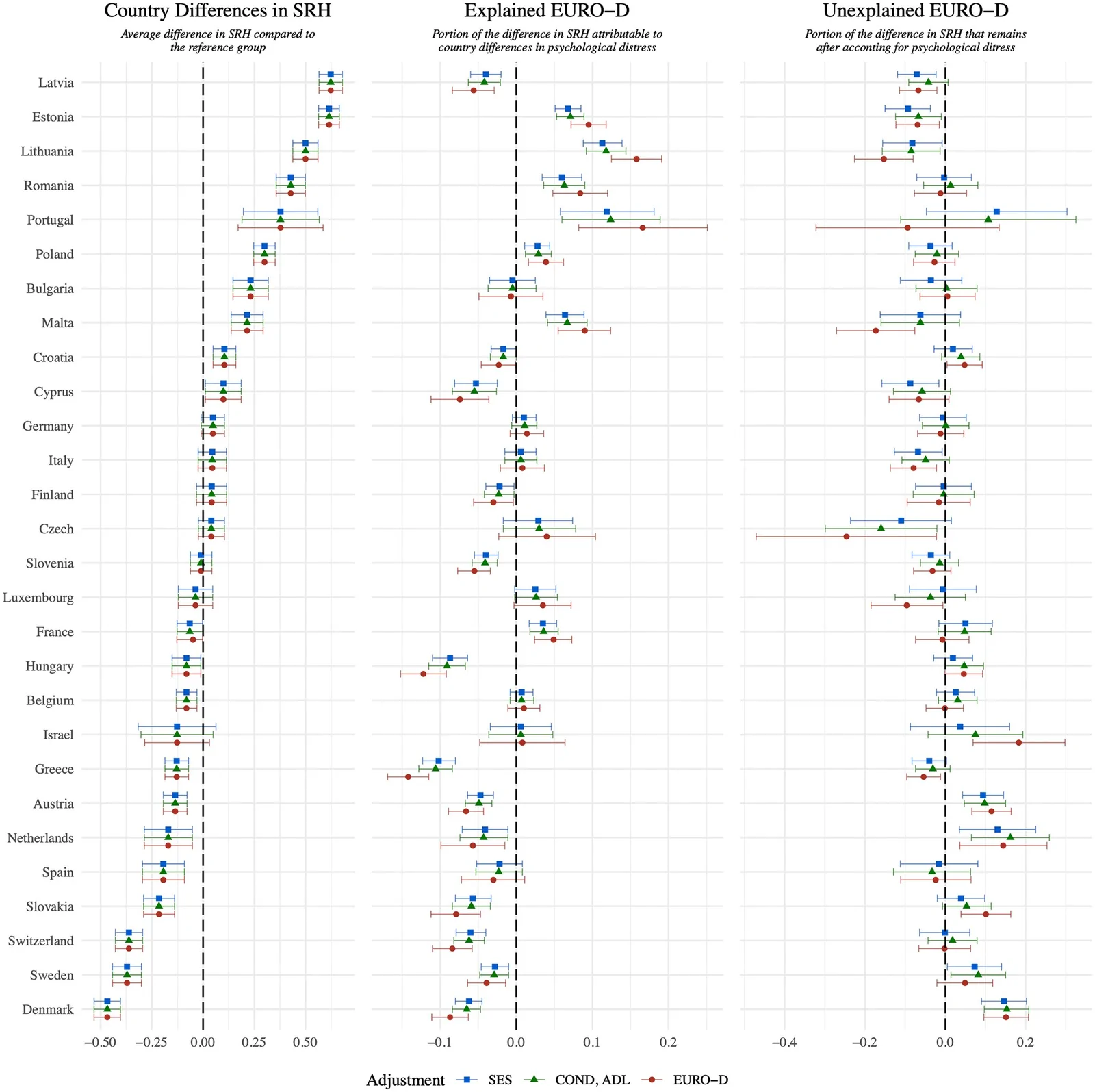
Country differences in SRH decomposed into total difference (left), the part explained by depressive symptoms (EUROD-D) levels (centre), and the part explained by differences in the SRH-depressive symptoms (EURO-D) association (right).

We observe that lower depression prevalence contributes to their SRH advantage in Nordic countries, as shown by negative explained components (Denmark: −0.062 [95%CI: −0.080 to −0.046]; Sweden: −0.028 [95%CI: −0.046 to −0.010]). However, the positive EURO-D unexplained components (Denmark: 0.146 [95%CI: 0.090–0.202]; Sweden: 0.073 [95%CI: 0.005–0.140]) indicate that the relationship between depressive symptoms and SRH is actually stronger in these countries than in the reference population.

Eastern European countries show a different configuration. Estonia’s positive EURO-D explained component (0.068 [95%CI: 0.051–0.085]) confirms that higher depression prevalence contributes to poorer SRH, while its negative unexplained component (−0.093 [95%CI: −0.150 to −0.037]) reveals that the depression-SRH association is weaker than in the reference population. Poland presents a similar pattern with depression prevalence playing a major role (explained: 0.028 [95%CI: 0.011–0.044]) but the depression-SRH relationship being slightly weaker than reference (unexplained: −0.037 [95%CI: −0.091 to 0.017]).

Mediterranean countries demonstrate yet another distinct pattern. Greece shows lower contribution from depression prevalence differences (explained: −0.102 [95%CI: −0.123 to 0.080]) and a relative negative EURO-D unexplained component (−0.040 [95%CI: −0.083 to 0.003]), indicating that the association between depressive symptoms and SRH is weaker than in the reference population. Spain and Italy present a similar but milder version of this pattern.

The stability of these EURO-D specific patterns across adjustment models strengthens their interpretation. Switzerland consistently shows near-zero unexplained components, indicating its depression-SRH relationship closely matches the reference population once prevalence differences are accounted for.

Figure 4 displays country variations in how depressive symptoms (EURO-D) influence SRH, decomposed into explained and unexplained components. The figure shows in bold countries where both the explained and unexplained coefficients are significant. Regular and grey characters indicate countries that have, respectively, at least one component significant, or both components non-significant.

**Figure 4:**
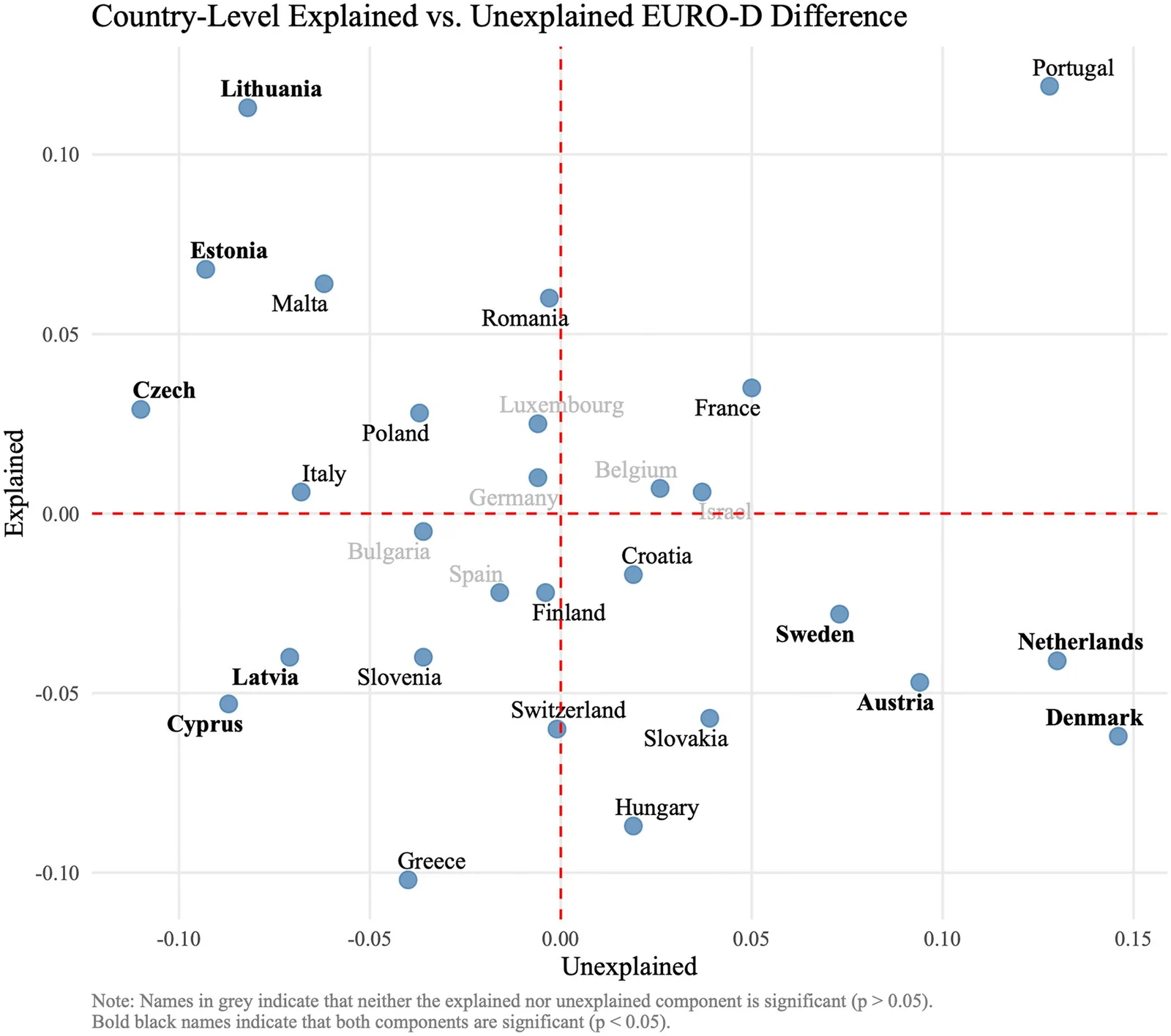
Country variations in SRH explained by depressive symptoms (EUROD-D) levels (*x*-axis), and by differences in the SRH-depressive symptoms (EURO-D) association (*y*-axis).

Three distinct patterns emerge. Countries including Latvia, Cyprus, and Slovenia show both significantly fewer depressive symptoms (negative explained component) and a negative depression-SRH association (negative unexplained component). This indicates their populations experience better mental health while simultaneously discounting depressive symptoms when evaluating overall health. Countries such as Estonia, Czech Republic, and Poland present higher depressive symptoms (positive explained component) coupled with a negative depression-SRH association (negative unexplained component), revealing that despite greater symptom prevalence, depressive symptoms carry less weight in health assessments. Countries including Denmark, Sweden, Austria, or the Netherlands demonstrate fewer depressive symptoms (negative explained component) but a positive depression-SRH association (positive unexplained component), suggesting that while depressive symptoms are generally better, these symptoms disproportionately affect health ratings. No fully significant results appear in the quadrant where both components would be positive: the study does not find strong evidence of countries suffering from the dual disadvantage of both elevated depressive symptoms and their amplified incorporation into health evaluations.

### Stratification by gender

We repeated the fully adjusted analyses with stratification by gender.

The pseudo-*R*² and partial correlations stratification by gender are shown in Table S4 and Fig. S2, respectively. No differences are observed across genders when looking at *R*-square analyses. Partial correlations reveal minimal gender differences in the strength of partial correlations. While some country-specific variations were observed, the overall pattern was consistent between men and women, suggesting that the underlying associations among these health measures are largely gender-invariant.

BO estimates, including the main difference and the explained and unexplained EURO-D components, are shown in Table S8 and Fig. S5. Across countries, no consistent gender difference in self-reported health (SRH) is observed, as CIs for males and females largely overlap. The explained EURO-D component contributes little to SRH variation. The unexplained EURO-D component differs significantly between males and females only in Czech Republic. In all other countries, CIs overlap and point estimates for males and females are similar, suggesting no meaningful gender difference in the role of psychological factors on SRH.

### Sensitivity analyses

Sensitivity analyses using interval regression models produced results consistent with our main findings (Fig. S6). We also replicated the analyses for 18 countries included in waves 4, 5, and 6 (pooled) (Table S9, Figs S9–S14) and found the same conclusions, suggesting that country differences in SRH perceptions are relatively stable over time.

## Discussion

This study brings three novel insights to the literature on SRH and cross-country comparisons.

First, depressive symptoms explained nearly as much variation in SRH as physical health conditions and functional limitations. This finding is novel because previous studies treated mental health as a confounder rather than a potential core component of SRH. Our results show that mental health contributes independently to self-assessed health status, even after adjusting for physical health, and, therefore, forms an integral part of how individuals evaluate their overall health.

Second, the association between depressive symptoms and SRH is not uniform across Europe. Using BO decomposition, we found that cross-country differences in this association cannot be reduced to differences in population composition such as age, income, or education. Instead, they reflect genuine contextual variation in how depressive symptoms translate into health ratings.

Third, these cross-country patterns broadly align with welfare state regimes [29]. Nordic social-democratic countries show low depressive symptom prevalence but strong SRH-depression links. Continental conservative countries show low prevalence but weaker links. Southern and Eastern European countries show higher depressive symptom prevalence but weaker SRH-depression links. This suggests that how societies’ organize welfare, healthcare, and labour markets may shape not only mental health outcomes but also the very meaning of health.

We also examined whether these patterns differ by gender. Gender differences in SRH were largely unexplained by differential weighting in depressive symptoms, indicating that men and women use similar criteria when assessing their health.

This study has several limitations. Our analysis focused on adults aged 55+, and SRH levels tend to be higher in SHARE than in other European surveys [5], suggesting a potentially healthier sample which might produce certain biases. The BO decomposition assumes linearity [30]; although non-linear alternatives exist [31], our sensitivity analyses treating SRH as continuous produced consistent results.

Our findings align with established gradients in European mental health [32, 33] but extend this work by showing that the meaning of SRH also varies across contexts. Differences in how depressive symptoms translate into health ratings likely reflect broader cultural and structural factors, including attitudes toward mental illness [34], professional norms [35], and national variation in health and mental health literacy [36–38].

For researchers and policymakers, these findings highlight the need for caution when interpreting SRH in cross-national analyses. Although SRH remains a valuable indicator of overall health, it is not a purely objective or universally comparable measure. Because depressive symptoms substantially contribute to SRH, cross-country differences may conflate variations in true health with differences in how mental wellbeing is valued. Accounting for mental health, or explicitly modelling its contribution, can help disentangle these effects.

## Conclusion

Self-Rated Health should be understood not only as a measure of health but as a culturally embedded perception reflecting both physical and mental dimensions of wellbeing.

## Supplementary Material

ckag178_Supplementary_Data

## Data Availability

SHARE data are available at https://share-eric.eu/ Key points Self-rated health incorporates mental health to a degree as substantial as physical health. The influence of depressive symptoms on self-rated health varies significantly across European countries. National differences in self-rated health are partly explained by variations in the prevalence of depressive symptoms. Gender gaps in self-rated health are primarily due to different levels of depressive symptoms, not how they are weighed in the assessment.
