# Supplementary material for: Cross-country differences in self-rated health: decomposing the influence of depressive symptoms
Source: Eur J Public Health. 2026 Sep 29;36(5):ckag178. doi: 10.1093/eurpub/ckag178 (PMC13622333; doi:10.1093/eurpub/ckag178)
Supplement: Supplementary file 1 [file ckag178_supplementary_data.zip › ejph-2025-11-om-1002-File021.docx]

# Table S1. Country descriptive statistics (wave 9)

| Country | **N** | **Available** |  | **SRH** | | |  | **EUROD** | | |  | **Health condition** | | |  | **ADL** | | |
| --- | --- | --- | --- | --- | --- | --- | --- | --- | --- | --- | --- | --- | --- | --- | --- | --- | --- | --- |
|  |  | **waves** |  | Mean | Median | SD |  | Mean | Median | SD |  | Mean | Median | SD |  | Mean | Median | SD |
|  |  | 9 |  |  |  |  |  |  |  |  |  |  |  |  |  |  |  |  |
| Austria | 3,405 | x |  | 3.01 | 3 | 1.05 |  | 2.10 | 2.00 | 2.01 |  | 0.65 | 1.00 | 0.48 |  | 0.09 | 0.00 | 0.29 |
| Belgium | 4,497 | x |  | 3.03 | 3 | 0.99 |  | 2.71 | 2.00 | 2.26 |  | 0.66 | 1.00 | 0.47 |  | 0.15 | 0.00 | 0.36 |
| Bulgaria | 831 | x |  | 3.32 | 3 | 1.01 |  | 2.28 | 1.00 | 2.63 |  | 0.72 | 1.00 | 0.45 |  | 0.09 | 0.00 | 0.28 |
| Croatia | 4,687 | x |  | 3.24 | 3 | 1.10 |  | 2.33 | 2.00 | 2.47 |  | 0.69 | 1.00 | 0.46 |  | 0.09 | 0.00 | 0.29 |
| Cyprus | 736 | x |  | 3.18 | 3 | 0.98 |  | 1.96 | 1.00 | 2.07 |  | 0.76 | 1.00 | 0.43 |  | 0.09 | 0.00 | 0.29 |
| Czech Republic | 3,319 | x |  | 3.12 | 3 | 0.85 |  | 2.44 | 2.00 | 2.17 |  | 0.76 | 1.00 | 0.43 |  | 0.13 | 0.00 | 0.34 |
| Denmark | 2,348 | x |  | 2.64 | 2 | 1.13 |  | 1.97 | 1.00 | 1.92 |  | 0.60 | 1.00 | 0.49 |  | 0.09 | 0.00 | 0.29 |
| Estonia | 4,486 | x |  | 3.73 | 4 | 0.89 |  | 2.93 | 3.00 | 2.23 |  | 0.69 | 1.00 | 0.46 |  | 0.14 | 0.00 | 0.35 |
| Finland | 1,758 | x |  | 3.19 | 3 | 0.97 |  | 2.35 | 2.00 | 1.86 |  | 0.72 | 1.00 | 0.45 |  | 0.10 | 0.00 | 0.31 |
| France | 2,909 | x |  | 3.14 | 3 | 1.01 |  | 2.78 | 2.00 | 2.18 |  | 0.62 | 1.00 | 0.48 |  | 0.11 | 0.00 | 0.32 |
| Germany | 4,467 | x |  | 3.21 | 3 | 0.99 |  | 2.49 | 2.00 | 2.05 |  | 0.69 | 1.00 | 0.46 |  | 0.12 | 0.00 | 0.33 |
| Greece | 3,112 | x |  | 3.16 | 3 | 0.95 |  | 1.71 | 1.00 | 2.34 |  | 0.80 | 1.00 | 0.40 |  | 0.08 | 0.00 | 0.28 |
| Hungary | 1,828 | x |  | 3.15 | 3 | 0.97 |  | 2.00 | 1.00 | 2.36 |  | 0.68 | 1.00 | 0.47 |  | 0.09 | 0.00 | 0.29 |
| Israel | 744 | x |  | 3.33 | 3 | 1.18 |  | 2.93 | 2.00 | 2.67 |  | 0.78 | 1.00 | 0.41 |  | 0.16 | 0.00 | 0.37 |
| Italy | 3,623 | x |  | 3.35 | 3 | 0.96 |  | 2.75 | 2.00 | 2.67 |  | 0.67 | 1.00 | 0.47 |  | 0.11 | 0.00 | 0.31 |
| Latvia | 1,716 | x |  | 3.75 | 4 | 0.75 |  | 2.15 | 2.00 | 2.19 |  | 0.69 | 1.00 | 0.46 |  | 0.06 | 0.00 | 0.25 |
| Lithuania | 1,414 | x |  | 3.61 | 4 | 0.81 |  | 3.30 | 3.00 | 2.42 |  | 0.74 | 1.00 | 0.44 |  | 0.14 | 0.00 | 0.34 |
| Luxembourg | 810 | x |  | 3.09 | 3 | 0.92 |  | 2.58 | 2.00 | 2.19 |  | 0.68 | 1.00 | 0.47 |  | 0.07 | 0.00 | 0.26 |
| Malta | 875 | x |  | 3.32 | 3 | 0.91 |  | 2.93 | 3.00 | 2.18 |  | 0.72 | 1.00 | 0.45 |  | 0.06 | 0.00 | 0.23 |
| Netherlands | 2,066 | x |  | 2.94 | 3 | 1.04 |  | 2.08 | 2.00 | 1.90 |  | 0.59 | 1.00 | 0.49 |  | 0.08 | 0.00 | 0.28 |
| Poland | 4,828 | x |  | 3.47 | 3 | 0.92 |  | 2.64 | 2.00 | 2.31 |  | 0.67 | 1.00 | 0.47 |  | 0.11 | 0.00 | 0.31 |
| Portugal | 1,638 | x |  | 3.72 | 4 | 1.00 |  | 3.31 | 3.00 | 2.54 |  | 0.74 | 1.00 | 0.44 |  | 0.17 | 0.00 | 0.37 |
| Romania | 1,492 | x |  | 3.49 | 3 | 0.92 |  | 2.82 | 2.00 | 2.72 |  | 0.66 | 1.00 | 0.47 |  | 0.11 | 0.00 | 0.31 |
| Slovakia | 1,055 | x |  | 2.88 | 3 | 0.94 |  | 1.96 | 1.00 | 2.10 |  | 0.55 | 1.00 | 0.50 |  | 0.05 | 0.00 | 0.21 |
| Slovenia | 4,444 | x |  | 3.16 | 3 | 1.00 |  | 2.15 | 2.00 | 2.07 |  | 0.67 | 1.00 | 0.47 |  | 0.11 | 0.00 | 0.31 |
| Spain | 2,087 | x |  | 3.32 | 3 | 1.05 |  | 2.84 | 2.00 | 2.66 |  | 0.67 | 1.00 | 0.47 |  | 0.16 | 0.00 | 0.37 |
| Sweden | 2,428 | x |  | 2.85 | 3 | 1.10 |  | 2.26 | 2.00 | 1.93 |  | 0.65 | 1.00 | 0.48 |  | 0.11 | 0.00 | 0.31 |
| Switzerland | 1,844 | x |  | 2.79 | 3 | 0.95 |  | 2.01 | 2.00 | 1.86 |  | 0.55 | 1.00 | 0.50 |  | 0.07 | 0.00 | 0.25 |
| Total/Mean | 69,447 | x |  | 3.22 | 3.11 | 0.98 |  | 2.46 | 1.93 | 2.25 |  | 0.68 | 1.00 | 0.46 |  | 0.11 | 0.00 | 0.30 |
