# Supplementary material for: Cross-country differences in self-rated health: decomposing the influence of depressive symptoms
Source: Eur J Public Health. 2026 Sep 29;36(5):ckag178. doi: 10.1093/eurpub/ckag178 (PMC13622333; doi:10.1093/eurpub/ckag178)
Supplement: Supplementary file 1 [file ckag178_supplementary_data.zip › ejph-2025-11-om-1002-File022.docx]

# Table S2. Pseudo R-square comparison across countries (linear regression)

|  | Socio-economic | | |  | + Health/ADL | | |  | +EURO-D | | |
| --- | --- | --- | --- | --- | --- | --- | --- | --- | --- | --- | --- |
| Country | R squared | CI lower | CI upper |  | R squared | CI_lower | CI_upper |  | R_squared | CI_lower | CI_upper |
| Austria | 0.142 | 0.119 | 0.171 |  | 0.257 | 0.228 | 0.287 |  | 0.345 | 0.311 | 0.384 |
| Belgium | 0.151 | 0.127 | 0.185 |  | 0.235 | 0.211 | 0.269 |  | 0.335 | 0.305 | 0.375 |
| Bulgaria | 0.220 | 0.165 | 0.285 |  | 0.344 | 0.291 | 0.417 |  | 0.400 | 0.350 | 0.480 |
| Switzerland | 0.153 | 0.118 | 0.205 |  | 0.246 | 0.224 | 0.296 |  | 0.301 | 0.262 | 0.349 |
| Cyprus | 0.212 | 0.154 | 0.276 |  | 0.301 | 0.239 | 0.371 |  | 0.313 | 0.258 | 0.388 |
| Czech republic | 0.128 | 0.090 | 0.308 |  | 0.335 | 0.273 | 0.447 |  | 0.369 | 0.337 | 0.496 |
| Germany | 0.165 | 0.143 | 0.195 |  | 0.290 | 0.262 | 0.322 |  | 0.351 | 0.326 | 0.378 |
| Denmark | 0.144 | 0.123 | 0.180 |  | 0.235 | 0.213 | 0.275 |  | 0.341 | 0.308 | 0.379 |
| Estonia | 0.216 | 0.199 | 0.235 |  | 0.318 | 0.299 | 0.344 |  | 0.373 | 0.348 | 0.405 |
| Spain | 0.120 | 0.093 | 0.204 |  | 0.255 | 0.222 | 0.343 |  | 0.314 | 0.266 | 0.383 |
| France | 0.116 | 0.089 | 0.153 |  | 0.216 | 0.192 | 0.251 |  | 0.292 | 0.262 | 0.330 |
| Finland | 0.182 | 0.148 | 0.241 |  | 0.286 | 0.250 | 0.340 |  | 0.338 | 0.294 | 0.393 |
| Greece | 0.276 | 0.247 | 0.311 |  | 0.363 | 0.332 | 0.395 |  | 0.391 | 0.363 | 0.428 |
| Croatia | 0.187 | 0.162 | 0.221 |  | 0.298 | 0.270 | 0.323 |  | 0.363 | 0.344 | 0.393 |
| Hungary | 0.194 | 0.164 | 0.256 |  | 0.272 | 0.238 | 0.318 |  | 0.348 | 0.315 | 0.406 |
| Israel | 0.326 | 0.239 | 0.443 |  | 0.458 | 0.413 | 0.550 |  | 0.504 | 0.464 | 0.591 |
| Italy | 0.146 | 0.117 | 0.192 |  | 0.255 | 0.222 | 0.300 |  | 0.303 | 0.272 | 0.356 |
| Lithuania | 0.147 | 0.116 | 0.190 |  | 0.261 | 0.223 | 0.309 |  | 0.346 | 0.310 | 0.390 |
| Luxembourg | 0.105 | 0.073 | 0.170 |  | 0.187 | 0.145 | 0.257 |  | 0.270 | 0.235 | 0.359 |
| Latvia | 0.224 | 0.186 | 0.274 |  | 0.323 | 0.274 | 0.374 |  | 0.389 | 0.356 | 0.453 |
| Malta | 0.088 | 0.062 | 0.138 |  | 0.131 | 0.091 | 0.186 |  | 0.184 | 0.142 | 0.244 |
| Netherlands | 0.135 | 0.060 | 0.239 |  | 0.230 | 0.162 | 0.355 |  | 0.331 | 0.261 | 0.453 |
| Poland | 0.202 | 0.175 | 0.233 |  | 0.313 | 0.290 | 0.340 |  | 0.370 | 0.343 | 0.394 |
| Portugal | 0.152 | 0.106 | 0.320 |  | 0.195 | 0.148 | 0.375 |  | 0.263 | 0.205 | 0.450 |
| Romania | 0.201 | 0.159 | 0.263 |  | 0.304 | 0.267 | 0.361 |  | 0.407 | 0.369 | 0.476 |
| Sweden | 0.129 | 0.104 | 0.183 |  | 0.221 | 0.187 | 0.280 |  | 0.303 | 0.272 | 0.355 |
| Slovenia | 0.181 | 0.157 | 0.211 |  | 0.282 | 0.260 | 0.317 |  | 0.305 | 0.285 | 0.338 |
| Slovakia | 0.211 | 0.162 | 0.290 |  | 0.376 | 0.336 | 0.428 |  | 0.467 | 0.426 | 0.520 |
