# Supplementary material for: Cross-country differences in self-rated health: decomposing the influence of depressive symptoms
Source: Eur J Public Health. 2026 Sep 29;36(5):ckag178. doi: 10.1093/eurpub/ckag178 (PMC13622333; doi:10.1093/eurpub/ckag178)
Supplement: Supplementary file 1 [file ckag178_supplementary_data.zip › ejph-2025-11-om-1002-File029.docx]

# Table S9. Country descriptive statistics (waves 4, 5, 6)

| Country | **N** | **Available**  **waves** | | |  | **SRH** | | |  | **EUROD** | | |  | **Health condition** | | |  | **ADL** | | |
| --- | --- | --- | --- | --- | --- | --- | --- | --- | --- | --- | --- | --- | --- | --- | --- | --- | --- | --- | --- | --- |
|  |  |  |  |  |  | Mean | Median | SD |  | Mean | Median | SD |  | Mean | Median | SD |  | Mean | Median | SD |
|  |  | 4 | 5 | 6 |  |  |  |  |  |  |  |  |  |  |  |  |  |  |  |  |
| Austria | 11,201 | x | x | x |  | 3.04 | 3.00 | 1.05 |  | 2.04 | 2.00 | 2.02 |  | 0.66 | 1.00 | 0.47 |  | 0.11 | 0.00 | 0.32 |
| Belgium | 13,713 | x | x | x |  | 3.05 | 3.00 | 0.97 |  | 2.56 | 2.00 | 2.22 |  | 0.68 | 1.00 | 0.47 |  | 0.17 | 0.00 | 0.38 |
| Croatia | 2,130 |  |  | x |  | 3.38 | 3.00 | 1.17 |  | 2.61 | 2.00 | 2.46 |  | 0.68 | 1.00 | 0.47 |  | 0.11 | 0.00 | 0.32 |
| Czech Republic | 14,395 | x | x | x |  | 3.44 | 3.00 | 0.98 |  | 2.33 | 2.00 | 2.22 |  | 0.74 | 1.00 | 0.44 |  | 0.13 | 0.00 | 0.33 |
| Denmark | 8,272 | x | x | x |  | 2.57 | 2.00 | 1.16 |  | 1.75 | 1.00 | 1.84 |  | 0.61 | 1.00 | 0.49 |  | 0.09 | 0.00 | 0.28 |
| Estonia | 15,936 | x | x | x |  | 3.93 | 4.00 | 0.82 |  | 3.13 | 3.00 | 2.29 |  | 0.73 | 1.00 | 0.44 |  | 0.18 | 0.00 | 0.38 |
| France | 12,216 | x | x | x |  | 3.27 | 3.00 | 1.02 |  | 2.86 | 2.00 | 2.29 |  | 0.65 | 1.00 | 0.48 |  | 0.14 | 0.00 | 0.35 |
| Germany | 9,832 | x | x | x |  | 3.30 | 3.00 | 0.98 |  | 2.26 | 2.00 | 1.99 |  | 0.69 | 1.00 | 0.46 |  | 0.12 | 0.00 | 0.32 |
| Greece | 4,230 |  |  | x |  | 3.11 | 3.00 | 1.01 |  | 2.64 | 2.00 | 2.62 |  | 0.72 | 1.00 | 0.45 |  | 0.09 | 0.00 | 0.28 |
| Hungary | 2,595 | x |  |  |  | 3.75 | 4.00 | 1.02 |  | 3.17 | 3.00 | 2.64 |  | 0.75 | 1.00 | 0.43 |  | 0.14 | 0.00 | 0.34 |
| Israel | 4,289 |  | x | x |  | 3.20 | 3.00 | 1.16 |  | 2.38 | 2.00 | 2.46 |  | 0.70 | 1.00 | 0.46 |  | 0.15 | 0.00 | 0.36 |
| Italy | 11,809 | x | x | x |  | 3.33 | 3.00 | 1.05 |  | 2.90 | 2.00 | 2.57 |  | 0.65 | 1.00 | 0.48 |  | 0.13 | 0.00 | 0.33 |
| Luxembourg | 2,690 |  | x | x |  | 3.10 | 3.00 | 1.04 |  | 2.48 | 2.00 | 2.21 |  | 0.70 | 1.00 | 0.46 |  | 0.12 | 0.00 | 0.32 |
| Netherlands | 6,018 | x | x |  |  | 2.98 | 3.00 | 1.03 |  | 1.85 | 1.00 | 1.93 |  | 0.57 | 1.00 | 0.49 |  | 0.08 | 0.00 | 0.27 |
| Slovenia | 8,717 | x | x | x |  | 3.38 | 3.00 | 1.00 |  | 2.39 | 2.00 | 2.08 |  | 0.70 | 1.00 | 0.46 |  | 0.12 | 0.00 | 0.32 |
| Spain | 14,318 | x | x | x |  | 3.42 | 3.00 | 1.02 |  | 2.62 | 2.00 | 2.65 |  | 0.70 | 1.00 | 0.46 |  | 0.14 | 0.00 | 0.35 |
| Sweden | 9,864 | x | x | x |  | 2.78 | 3.00 | 1.14 |  | 1.97 | 2.00 | 1.85 |  | 0.62 | 1.00 | 0.49 |  | 0.09 | 0.00 | 0.29 |
| Switzerland | 8,381 | x | x | x |  | 2.72 | 3.00 | 0.95 |  | 1.91 | 2.00 | 1.80 |  | 0.54 | 1.00 | 0.50 |  | 0.07 | 0.00 | 0.25 |
