# Supplementary material for: Cross-country differences in self-rated health: decomposing the influence of depressive symptoms
Source: Eur J Public Health. 2026 Sep 29;36(5):ckag178. doi: 10.1093/eurpub/ckag178 (PMC13622333; doi:10.1093/eurpub/ckag178)
Supplement: Supplementary file 1 [file ckag178_supplementary_data.zip › ejph-2025-11-om-1002-File024.docx]

# Table S4. Pseudo R-square comparison across countries (linear regression), stratification by gender

**Female**

|  | Socio-economic | | |  | + Health/ADL | | |  | +EURO-D | | |
| --- | --- | --- | --- | --- | --- | --- | --- | --- | --- | --- | --- |
| Country | R squared | CI lower | CI upper |  | R squared | CI_lower | CI_upper |  | R_squared | CI_lower | CI_upper |
| Austria | 0.142 | 0.114 | 0.170 |  | 0.257 | 0.235 | 0.300 |  | 0.345 | 0.317 | 0.379 |
| Belgium | 0.151 | 0.131 | 0.192 |  | 0.235 | 0.214 | 0.265 |  | 0.335 | 0.304 | 0.376 |
| Bulgaria | 0.220 | 0.177 | 0.269 |  | 0.344 | 0.295 | 0.414 |  | 0.400 | 0.349 | 0.485 |
| Switzerland | 0.153 | 0.115 | 0.199 |  | 0.246 | 0.208 | 0.305 |  | 0.301 | 0.264 | 0.357 |
| Cyprus | 0.212 | 0.159 | 0.285 |  | 0.301 | 0.251 | 0.391 |  | 0.313 | 0.275 | 0.406 |
| Czech republic | 0.128 | 0.083 | 0.309 |  | 0.335 | 0.274 | 0.451 |  | 0.369 | 0.334 | 0.493 |
| Germany | 0.165 | 0.143 | 0.196 |  | 0.290 | 0.267 | 0.325 |  | 0.351 | 0.326 | 0.378 |
| Denmark | 0.144 | 0.119 | 0.187 |  | 0.235 | 0.210 | 0.290 |  | 0.341 | 0.315 | 0.374 |
| Estonia | 0.216 | 0.193 | 0.239 |  | 0.318 | 0.299 | 0.347 |  | 0.373 | 0.348 | 0.398 |
| Spain | 0.120 | 0.083 | 0.177 |  | 0.255 | 0.213 | 0.322 |  | 0.314 | 0.272 | 0.400 |
| France | 0.116 | 0.094 | 0.150 |  | 0.216 | 0.187 | 0.263 |  | 0.292 | 0.262 | 0.333 |
| Finland | 0.182 | 0.136 | 0.239 |  | 0.286 | 0.245 | 0.343 |  | 0.338 | 0.290 | 0.400 |
| Greece | 0.276 | 0.249 | 0.314 |  | 0.363 | 0.333 | 0.396 |  | 0.391 | 0.362 | 0.421 |
| Croatia | 0.187 | 0.161 | 0.216 |  | 0.298 | 0.276 | 0.330 |  | 0.363 | 0.338 | 0.390 |
| Hungary | 0.194 | 0.163 | 0.243 |  | 0.272 | 0.239 | 0.316 |  | 0.348 | 0.311 | 0.406 |
| Israel | 0.326 | 0.240 | 0.432 |  | 0.458 | 0.370 | 0.547 |  | 0.504 | 0.437 | 0.594 |
| Italy | 0.146 | 0.109 | 0.187 |  | 0.255 | 0.220 | 0.299 |  | 0.303 | 0.262 | 0.343 |
| Lithuania | 0.147 | 0.123 | 0.188 |  | 0.261 | 0.226 | 0.300 |  | 0.346 | 0.313 | 0.392 |
| Luxembourg | 0.105 | 0.075 | 0.187 |  | 0.187 | 0.143 | 0.276 |  | 0.270 | 0.220 | 0.344 |
| Latvia | 0.224 | 0.191 | 0.264 |  | 0.323 | 0.283 | 0.374 |  | 0.389 | 0.358 | 0.450 |
| Malta | 0.088 | 0.068 | 0.143 |  | 0.131 | 0.095 | 0.199 |  | 0.184 | 0.155 | 0.259 |
| Netherlands | 0.135 | 0.081 | 0.247 |  | 0.230 | 0.160 | 0.354 |  | 0.331 | 0.267 | 0.440 |
| Poland | 0.202 | 0.179 | 0.229 |  | 0.313 | 0.283 | 0.349 |  | 0.370 | 0.345 | 0.397 |
| Portugal | 0.152 | 0.095 | 0.293 |  | 0.195 | 0.160 | 0.410 |  | 0.263 | 0.205 | 0.456 |
| Romania | 0.201 | 0.154 | 0.261 |  | 0.304 | 0.258 | 0.370 |  | 0.407 | 0.355 | 0.448 |
| Sweden | 0.129 | 0.100 | 0.172 |  | 0.221 | 0.187 | 0.271 |  | 0.303 | 0.272 | 0.363 |
| Slovenia | 0.181 | 0.163 | 0.211 |  | 0.282 | 0.262 | 0.312 |  | 0.305 | 0.286 | 0.333 |
| Slovakia | 0.211 | 0.174 | 0.275 |  | 0.376 | 0.339 | 0.433 |  | 0.467 | 0.426 | 0.524 |

Male

|  | Socio-economic | | |  | + Health/ADL | | |  | +EURO-D | | |
| --- | --- | --- | --- | --- | --- | --- | --- | --- | --- | --- | --- |
| Country | R squared | CI lower | CI upper |  | R squared | CI_lower | CI_upper |  | R_squared | CI_lower | CI_upper |
| Austria | 0.142 | 0.121 | 0.171 |  | 0.257 | 0.221 | 0.300 |  | 0.345 | 0.319 | 0.384 |
| Belgium | 0.151 | 0.123 | 0.184 |  | 0.235 | 0.208 | 0.268 |  | 0.335 | 0.310 | 0.374 |
| Bulgaria | 0.220 | 0.179 | 0.288 |  | 0.344 | 0.296 | 0.423 |  | 0.400 | 0.339 | 0.477 |
| Switzerland | 0.153 | 0.118 | 0.199 |  | 0.246 | 0.209 | 0.291 |  | 0.301 | 0.263 | 0.347 |
| Cyprus | 0.212 | 0.164 | 0.278 |  | 0.301 | 0.239 | 0.372 |  | 0.313 | 0.264 | 0.400 |
| Czech republic | 0.128 | 0.092 | 0.285 |  | 0.335 | 0.278 | 0.454 |  | 0.369 | 0.316 | 0.478 |
| Germany | 0.165 | 0.137 | 0.191 |  | 0.290 | 0.260 | 0.318 |  | 0.351 | 0.326 | 0.379 |
| Denmark | 0.144 | 0.121 | 0.183 |  | 0.235 | 0.214 | 0.273 |  | 0.341 | 0.314 | 0.375 |
| Estonia | 0.216 | 0.193 | 0.241 |  | 0.318 | 0.292 | 0.345 |  | 0.373 | 0.346 | 0.397 |
| Spain | 0.120 | 0.094 | 0.182 |  | 0.255 | 0.225 | 0.321 |  | 0.314 | 0.274 | 0.399 |
| France | 0.116 | 0.096 | 0.155 |  | 0.216 | 0.185 | 0.265 |  | 0.292 | 0.262 | 0.325 |
| Finland | 0.182 | 0.139 | 0.244 |  | 0.286 | 0.248 | 0.344 |  | 0.338 | 0.305 | 0.397 |
| Greece | 0.276 | 0.241 | 0.319 |  | 0.363 | 0.329 | 0.404 |  | 0.391 | 0.362 | 0.438 |
| Croatia | 0.187 | 0.159 | 0.221 |  | 0.298 | 0.272 | 0.330 |  | 0.363 | 0.336 | 0.391 |
| Hungary | 0.194 | 0.147 | 0.248 |  | 0.272 | 0.230 | 0.320 |  | 0.348 | 0.313 | 0.401 |
| Israel | 0.326 | 0.256 | 0.451 |  | 0.458 | 0.401 | 0.564 |  | 0.504 | 0.451 | 0.598 |
| Italy | 0.146 | 0.117 | 0.186 |  | 0.255 | 0.215 | 0.293 |  | 0.303 | 0.265 | 0.350 |
| Lithuania | 0.147 | 0.119 | 0.189 |  | 0.261 | 0.231 | 0.307 |  | 0.346 | 0.302 | 0.401 |
| Luxembourg | 0.105 | 0.072 | 0.185 |  | 0.187 | 0.166 | 0.267 |  | 0.270 | 0.223 | 0.356 |
| Latvia | 0.224 | 0.185 | 0.281 |  | 0.323 | 0.289 | 0.388 |  | 0.389 | 0.343 | 0.447 |
| Malta | 0.088 | 0.066 | 0.151 |  | 0.131 | 0.106 | 0.198 |  | 0.184 | 0.147 | 0.257 |
| Netherlands | 0.135 | 0.085 | 0.218 |  | 0.230 | 0.158 | 0.332 |  | 0.331 | 0.271 | 0.451 |
| Poland | 0.202 | 0.177 | 0.229 |  | 0.313 | 0.284 | 0.349 |  | 0.370 | 0.335 | 0.403 |
| Portugal | 0.152 | 0.098 | 0.319 |  | 0.195 | 0.149 | 0.399 |  | 0.263 | 0.218 | 0.465 |
| Romania | 0.201 | 0.151 | 0.252 |  | 0.304 | 0.266 | 0.371 |  | 0.407 | 0.367 | 0.466 |
| Sweden | 0.129 | 0.102 | 0.183 |  | 0.221 | 0.189 | 0.267 |  | 0.303 | 0.269 | 0.351 |
| Slovenia | 0.181 | 0.155 | 0.211 |  | 0.282 | 0.258 | 0.310 |  | 0.305 | 0.276 | 0.336 |
| Slovakia | 0.211 | 0.167 | 0.278 |  | 0.376 | 0.336 | 0.432 |  | 0.467 | 0.433 | 0.516 |

(See figure S2)
