# Supplementary material for: Cross-country differences in self-rated health: decomposing the influence of depressive symptoms
Source: Eur J Public Health. 2026 Sep 29;36(5):ckag178. doi: 10.1093/eurpub/ckag178 (PMC13622333; doi:10.1093/eurpub/ckag178)
Supplement: Supplementary file 1 [file ckag178_supplementary_data.zip › ejph-2025-11-om-1002-File027.docx]

# Table S7. Blinder Oaxaca Decomposition, Linear results

### Austria

| Oaxaca decomposition | | |  |  |  |  |
| --- | --- | --- | --- | --- | --- | --- |
|  | srh | | srh | | srh | |
| group_1 | 2.967 | *** | 2.967 | *** | 2.967 | *** |
|  | [2.926 3.008] |  | [2.926 3.008] |  | [2.926 3.008] |  |
| group_2 | 3.103 | *** | 3.103 | *** | 3.103 | *** |
|  | [3.062 3.144] |  | [3.062 3.144] |  | [3.062 3.144] |  |
| difference | -0.136 | *** | -0.136 | *** | -0.136 | *** |
|  | [-0.194 -0.078] |  | [-0.194 -0.079] |  | [-0.194 -0.079] |  |
| explained | -0.066 | *** | 0.005 |  | 0.043 |  |
|  | [-0.089 -0.043] |  | [-0.027 0.036] |  | [-0.012 0.099] |  |
| unexplained | -0.071 | ** | -0.141 | *** | -0.180 | *** |
|  | [-0.123 -0.018] |  | [-0.190 -0.093] |  | [-0.236 -0.124] |  |
| EUROD | -0.066 | *** | -0.049 | *** | -0.047 | *** |
|  | [-0.089 -0.043] |  | [-0.067 -0.032] |  | [-0.064 -0.030] |  |
| ADL |  |  | -0.003 |  | -0.003 |  |
|  |  |  | [-0.012 0.006] |  | [-0.010 0.005] |  |
| chron |  |  | 0.057 | *** | 0.048 | *** |
|  |  |  | [0.039 0.075] |  | [0.033 0.064] |  |
| agegroup1 | |  |  |  | 0.024 | ** |
|  |  |  |  |  | [0.006 0.041] |  |
| agegroup3 | |  |  |  | 0.014 | *** |
|  |  |  |  |  | [0.008 0.021] |  |
| female |  |  |  |  | -0.002 |  |
|  |  |  |  |  | [-0.005 0.001] |  |
| partnerinhh== 3.0000 | | |  |  | -0.001 |  |
|  |  |  |  |  | [-0.002 0.001] |  |
| bieduc |  |  |  |  | -0.016 | *** |
|  |  |  |  |  | [-0.025 -0.008] |  |
| employment2 | |  |  |  | -0.020 | ** |
|  |  |  |  |  | [-0.033 -0.007] |  |
| employment3 | |  |  |  | -0.008 | * |
|  |  |  |  |  | [-0.015 -0.001] |  |
| employment4 | |  |  |  | 0.077 | *** |
|  |  |  |  |  | [0.044 0.111] |  |
| RECODE of hin_adjlog (hin_adjlog) | | | |  | -0.005 |  |
|  |  |  |  |  | [-0.030 0.021] |  |
| housing==not tenant | |  |  |  | -0.020 |  |
|  |  |  |  |  | [-0.051 0.011] |  |
| EUROD | 0.115 | *** | 0.098 | *** | 0.094 | *** |
|  | [0.066 0.164] |  | [0.047 0.150] |  | [0.043 0.145] |  |
| ADL |  |  | 0.010 |  | 0.009 |  |
|  |  |  | [-0.004 0.023] |  | [-0.004 0.022] |  |
| chron |  |  | -0.055 |  | -0.047 |  |
|  |  |  | [-0.125 0.016] |  | [-0.116 0.022] |  |
| agegroup1 | |  |  |  | -0.043 |  |
|  |  |  |  |  | [-0.091 0.004] |  |
| agegroup3 | |  |  |  | -0.002 |  |
|  |  |  |  |  | [-0.022 0.019] |  |
| female |  |  |  |  | -0.021 |  |
|  |  |  |  |  | [-0.081 0.040] |  |
| partnerinhh== 3.0000 | | |  |  | 0.012 |  |
|  |  |  |  |  | [-0.036 0.059] |  |
| bieduc |  |  |  |  | -0.013 |  |
|  |  |  |  |  | [-0.049 0.023] |  |
| employment2 | |  |  |  | 0.004 |  |
|  |  |  |  |  | [-0.001 0.009] |  |
| employment3 | |  |  |  | -0.008 |  |
|  |  |  |  |  | [-0.022 0.006] |  |
| employment4 | |  |  |  | -0.106 |  |
|  |  |  |  |  | [-0.216 0.004] |  |
| RECODE of hin_adjlog (hin_adjlog) | | | |  | -0.782 | ** |
|  |  |  |  |  | [-1.325 -0.239] |  |
| housing==not tenant | |  |  |  | 0.042 |  |
|  |  |  |  |  | [-0.016 0.099] |  |
| Intercept | -0.186 | *** | -0.194 | *** | 0.683 | * |
|  | [-0.259 -0.112] |  | [-0.287 -0.102] |  | [0.102 1.263] |  |
| N | 68380 |  | 68380 |  | 68380 |  |
| *** p<.001, ** p<.01, * p<.05 | | |  |  |  |  |

### Belgium

| Oaxaca decomposition | | |  |  |  |  |
| --- | --- | --- | --- | --- | --- | --- |
|  | srh | | srh | | srh | |
| group_1 | 3.023 | *** | 3.023 | *** | 3.023 | *** |
|  | [2.994 3.051] |  | [2.994 3.052] |  | [2.993 3.052] |  |
| group_2 | 3.103 | *** | 3.103 | *** | 3.103 | *** |
|  | [3.062 3.144] |  | [3.062 3.144] |  | [3.062 3.144] |  |
| difference | -0.081 | ** | -0.081 | ** | -0.081 | ** |
|  | [-0.131 -0.030] |  | [-0.131 -0.030] |  | [-0.131 -0.030] |  |
| explained | 0.010 |  | 0.105 | *** | 0.129 | *** |
|  | [-0.011 0.031] |  | [0.075 0.134] |  | [0.081 0.177] |  |
| unexplained | -0.090 | *** | -0.185 | *** | -0.210 | *** |
|  | [-0.136 -0.045] |  | [-0.227 -0.143] |  | [-0.255 -0.165] |  |
| EUROD | 0.010 |  | 0.007 |  | 0.007 |  |
|  | [-0.011 0.031] |  | [-0.008 0.023] |  | [-0.008 0.022] |  |
| ADL |  |  | 0.043 | *** | 0.036 | *** |
|  |  |  | [0.032 0.055] |  | [0.026 0.047] |  |
| chron |  |  | 0.054 | *** | 0.046 | *** |
|  |  |  | [0.038 0.071] |  | [0.031 0.060] |  |
| agegroup1 | |  |  |  | 0.013 | * |
|  |  |  |  |  | [0.003 0.023] |  |
| agegroup3 | |  |  |  | 0.007 | *** |
|  |  |  |  |  | [0.004 0.010] |  |
| female |  |  |  |  | 0.004 |  |
|  |  |  |  |  | [-0.000 0.007] |  |
| partnerinhh== 3.0000 | | |  |  | 0.003 |  |
|  |  |  |  |  | [-0.005 0.012] |  |
| bieduc |  |  |  |  | -0.016 | *** |
|  |  |  |  |  | [-0.024 -0.008] |  |
| employment2 | |  |  |  | -0.001 |  |
|  |  |  |  |  | [-0.006 0.003] |  |
| employment3 | |  |  |  | 0.000 |  |
|  |  |  |  |  | [-0.003 0.002] |  |
| employment4 | |  |  |  | 0.041 | *** |
|  |  |  |  |  | [0.023 0.059] |  |
| RECODE of hin_adjlog (hin_adjlog) | | | |  | -0.006 |  |
|  |  |  |  |  | [-0.038 0.027] |  |
| housing==not tenant | |  |  |  | -0.004 |  |
|  |  |  |  |  | [-0.009 0.002] |  |
| EUROD | -0.001 |  | 0.031 |  | 0.026 |  |
|  | [-0.048 0.045] |  | [-0.017 0.079] |  | [-0.022 0.073] |  |
| ADL |  |  | -0.012 |  | -0.012 |  |
|  |  |  | [-0.032 0.007] |  | [-0.031 0.008] |  |
| chron |  |  | -0.175 | *** | -0.143 | *** |
|  |  |  | [-0.236 -0.114] |  | [-0.204 -0.083] |  |
| agegroup1 | |  |  |  | -0.003 |  |
|  |  |  |  |  | [-0.051 0.044] |  |
| agegroup3 | |  |  |  | -0.006 |  |
|  |  |  |  |  | [-0.018 0.006] |  |
| female |  |  |  |  | 0.027 |  |
|  |  |  |  |  | [-0.022 0.076] |  |
| partnerinhh== 3.0000 | | |  |  | 0.000 |  |
|  |  |  |  |  | [-0.030 0.030] |  |
| bieduc |  |  |  |  | 0.028 |  |
|  |  |  |  |  | [-0.004 0.060] |  |
| employment2 | |  |  |  | 0.019 | * |
|  |  |  |  |  | [0.001 0.037] |  |
| employment3 | |  |  |  | -0.002 |  |
|  |  |  |  |  | [-0.021 0.017] |  |
| employment4 | |  |  |  | -0.057 |  |
|  |  |  |  |  | [-0.131 0.016] |  |
| RECODE of hin_adjlog (hin_adjlog) | | | |  | -0.301 |  |
|  |  |  |  |  | [-0.673 0.071] |  |
| housing==not tenant | |  |  |  | 0.024 |  |
|  |  |  |  |  | [-0.002 0.050] |  |
| Intercept | -0.089 | ** | -0.029 |  | 0.190 |  |
|  | [-0.154 -0.024] |  | [-0.110 0.052] |  | [-0.215 0.596] |  |
| N | 71322 |  | 71322 |  | 71322 |  |
| *** p<.001, ** p<.01, * p<.05 | | |  |  |  |  |

### Bulgaria

| Oaxaca decomposition | | |  |  |  |  |
| --- | --- | --- | --- | --- | --- | --- |
|  | srh | | srh | | srh | |
| group_1 | 3.335 | *** | 3.335 | *** | 3.335 | *** |
|  | [3.259 3.411] |  | [3.259 3.411] |  | [3.259 3.411] |  |
| group_2 | 3.103 | *** | 3.103 | *** | 3.103 | *** |
|  | [3.062 3.144] |  | [3.062 3.144] |  | [3.062 3.144] |  |
| difference | 0.232 | *** | 0.232 | *** | 0.232 | *** |
|  | [0.146 0.318] |  | [0.146 0.318] |  | [0.146 0.318] |  |
| explained | -0.007 |  | 0.090 | *** | 0.177 | *** |
|  | [-0.049 0.035] |  | [0.038 0.142] |  | [0.118 0.236] |  |
| unexplained | 0.239 | *** | 0.142 | *** | 0.055 |  |
|  | [0.164 0.314] |  | [0.073 0.211] |  | [-0.012 0.122] |  |
| EUROD | -0.007 |  | -0.005 |  | -0.005 |  |
|  | [-0.049 0.035] |  | [-0.037 0.026] |  | [-0.035 0.025] |  |
| ADL |  |  | 0.004 |  | 0.004 |  |
|  |  |  | [-0.011 0.020] |  | [-0.009 0.016] |  |
| chron |  |  | 0.091 | *** | 0.077 | *** |
|  |  |  | [0.066 0.116] |  | [0.055 0.099] |  |
| agegroup1 | |  |  |  | 0.025 | * |
|  |  |  |  |  | [0.006 0.045] |  |
| agegroup3 | |  |  |  | 0.009 | *** |
|  |  |  |  |  | [0.004 0.014] |  |
| female |  |  |  |  | -0.007 | * |
|  |  |  |  |  | [-0.013 -0.000] |  |
| partnerinhh== 3.0000 | | |  |  | -0.003 |  |
|  |  |  |  |  | [-0.012 0.005] |  |
| bieduc |  |  |  |  | 0.023 | *** |
|  |  |  |  |  | [0.012 0.034] |  |
| employment2 | |  |  |  | -0.010 | * |
|  |  |  |  |  | [-0.018 -0.002] |  |
| employment3 | |  |  |  | -0.015 | * |
|  |  |  |  |  | [-0.027 -0.003] |  |
| employment4 | |  |  |  | 0.070 | *** |
|  |  |  |  |  | [0.039 0.101] |  |
| RECODE of hin_adjlog (hin_adjlog) | | | |  | 0.001 |  |
|  |  |  |  |  | [-0.007 0.010] |  |
| housing==not tenant | |  |  |  | 0.007 |  |
|  |  |  |  |  | [-0.004 0.019] |  |
| EUROD | 0.005 |  | 0.003 |  | -0.036 |  |
|  | [-0.063 0.074] |  | [-0.073 0.079] |  | [-0.112 0.041] |  |
| ADL |  |  | -0.017 |  | -0.010 |  |
|  |  |  | [-0.041 0.008] |  | [-0.034 0.013] |  |
| chron |  |  | 0.063 |  | 0.035 |  |
|  |  |  | [-0.052 0.179] |  | [-0.080 0.149] |  |
| agegroup1 | |  |  |  | -0.040 |  |
|  |  |  |  |  | [-0.113 0.034] |  |
| agegroup3 | |  |  |  | -0.017 |  |
|  |  |  |  |  | [-0.049 0.016] |  |
| female |  |  |  |  | 0.104 | * |
|  |  |  |  |  | [0.005 0.202] |  |
| partnerinhh== 3.0000 | | |  |  | -0.020 |  |
|  |  |  |  |  | [-0.104 0.064] |  |
| bieduc |  |  |  |  | 0.007 |  |
|  |  |  |  |  | [-0.020 0.034] |  |
| employment2 | |  |  |  | 0.012 |  |
|  |  |  |  |  | [-0.005 0.029] |  |
| employment3 | |  |  |  | 0.002 |  |
|  |  |  |  |  | [-0.009 0.012] |  |
| employment4 | |  |  |  | 0.088 |  |
|  |  |  |  |  | [-0.066 0.241] |  |
| RECODE of hin_adjlog (hin_adjlog) | | | |  | -0.117 |  |
|  |  |  |  |  | [-0.703 0.469] |  |
| housing==not tenant | |  |  |  | 0.010 |  |
|  |  |  |  |  | [-0.005 0.026] |  |
| Intercept | 0.234 | *** | 0.092 |  | 0.037 |  |
|  | [0.132 0.336] |  | [-0.041 0.226] |  | [-0.620 0.694] |  |
| N | 65971 |  | 65971 |  | 65971 |  |
| *** p<.001, ** p<.01, * p<.05 | | |  |  |  |  |

### Croatia

| Oaxaca decomposition | | |  |  |  |  |
| --- | --- | --- | --- | --- | --- | --- |
|  | srh | | srh | | srh | |
| group_1 | 3.208 | *** | 3.208 | *** | 3.208 | *** |
|  | [3.170 3.245] |  | [3.170 3.245] |  | [3.170 3.245] |  |
| group_2 | 3.103 | *** | 3.103 | *** | 3.103 | *** |
|  | [3.062 3.144] |  | [3.062 3.144] |  | [3.062 3.144] |  |
| difference | 0.104 | *** | 0.104 | *** | 0.104 | *** |
|  | [0.049 0.160] |  | [0.049 0.160] |  | [0.049 0.160] |  |
| explained | -0.023 | * | 0.050 | ** | 0.122 | *** |
|  | [-0.046 -0.001] |  | [0.020 0.081] |  | [0.086 0.159] |  |
| unexplained | 0.128 | *** | 0.054 | * | -0.018 |  |
|  | [0.078 0.178] |  | [0.009 0.100] |  | [-0.060 0.024] |  |
| EUROD | -0.023 | * | -0.017 | * | -0.017 | * |
|  | [-0.046 -0.001] |  | [-0.034 -0.000] |  | [-0.033 -0.000] |  |
| ADL |  |  | -0.003 |  | -0.002 |  |
|  |  |  | [-0.011 0.006] |  | [-0.009 0.005] |  |
| chron |  |  | 0.070 | *** | 0.059 | *** |
|  |  |  | [0.053 0.088] |  | [0.044 0.075] |  |
| agegroup1 | |  |  |  | 0.014 | * |
|  |  |  |  |  | [0.003 0.025] |  |
| agegroup3 | |  |  |  | 0.004 | *** |
|  |  |  |  |  | [0.002 0.007] |  |
| female |  |  |  |  | -0.001 |  |
|  |  |  |  |  | [-0.004 0.001] |  |
| partnerinhh== 3.0000 | | |  |  | 0.000 |  |
|  |  |  |  |  | [-0.001 0.001] |  |
| bieduc |  |  |  |  | 0.019 | *** |
|  |  |  |  |  | [0.011 0.028] |  |
| employment2 | |  |  |  | -0.008 | * |
|  |  |  |  |  | [-0.014 -0.001] |  |
| employment3 | |  |  |  | -0.001 |  |
|  |  |  |  |  | [-0.004 0.002] |  |
| employment4 | |  |  |  | 0.049 | *** |
|  |  |  |  |  | [0.027 0.070] |  |
| RECODE of hin_adjlog (hin_adjlog) | | | |  | 0.002 |  |
|  |  |  |  |  | [-0.008 0.012] |  |
| housing==not tenant | |  |  |  | 0.004 |  |
|  |  |  |  |  | [-0.002 0.011] |  |
| EUROD | 0.048 | * | 0.039 |  | 0.019 |  |
|  | [0.004 0.092] |  | [-0.009 0.086] |  | [-0.028 0.067] |  |
| ADL |  |  | 0.010 |  | 0.009 |  |
|  |  |  | [-0.003 0.023] |  | [-0.003 0.022] |  |
| chron |  |  | 0.047 |  | 0.031 |  |
|  |  |  | [-0.024 0.117] |  | [-0.040 0.101] |  |
| agegroup1 | |  |  |  | -0.009 |  |
|  |  |  |  |  | [-0.062 0.045] |  |
| agegroup3 | |  |  |  | -0.004 |  |
|  |  |  |  |  | [-0.017 0.010] |  |
| female |  |  |  |  | 0.045 |  |
|  |  |  |  |  | [-0.013 0.103] |  |
| partnerinhh== 3.0000 | | |  |  | 0.024 |  |
|  |  |  |  |  | [-0.019 0.067] |  |
| bieduc |  |  |  |  | -0.012 |  |
|  |  |  |  |  | [-0.032 0.008] |  |
| employment2 | |  |  |  | 0.013 |  |
|  |  |  |  |  | [-0.001 0.028] |  |
| employment3 | |  |  |  | 0.017 |  |
|  |  |  |  |  | [-0.003 0.036] |  |
| employment4 | |  |  |  | 0.065 |  |
|  |  |  |  |  | [-0.017 0.146] |  |
| RECODE of hin_adjlog (hin_adjlog) | | | |  | -0.033 |  |
|  |  |  |  |  | [-0.373 0.307] |  |
| housing==not tenant | |  |  |  | 0.008 |  |
|  |  |  |  |  | [-0.007 0.022] |  |
| Intercept | 0.080 | * | -0.041 |  | -0.191 |  |
|  | [0.011 0.149] |  | [-0.129 0.048] |  | [-0.601 0.218] |  |
| N | 69461 |  | 69461 |  | 69461 |  |
| *** p<.001, ** p<.01, * p<.05 | | |  |  |  |  |

### Cyprus

| Oaxaca decomposition | | |  |  |  |  |
| --- | --- | --- | --- | --- | --- | --- |
|  | srh | | srh | | srh | |
| group_1 | 3.202 | *** | 3.202 | *** | 3.202 | *** |
|  | [3.125 3.279] |  | [3.124 3.279] |  | [3.124 3.280] |  |
| group_2 | 3.103 | *** | 3.103 | *** | 3.103 | *** |
|  | [3.062 3.144] |  | [3.062 3.144] |  | [3.062 3.144] |  |
| difference | 0.099 | * | 0.099 | * | 0.099 | * |
|  | [0.011 0.186] |  | [0.011 0.186] |  | [0.011 0.186] |  |
| explained | -0.074 | *** | 0.073 | ** | 0.165 | *** |
|  | [-0.112 -0.036] |  | [0.022 0.124] |  | [0.106 0.223] |  |
| unexplained | 0.173 | *** | 0.026 |  | -0.066 |  |
|  | [0.091 0.254] |  | [-0.050 0.101] |  | [-0.136 0.004] |  |
| EUROD | -0.074 | *** | -0.055 | *** | -0.053 | *** |
|  | [-0.112 -0.036] |  | [-0.084 -0.026] |  | [-0.081 -0.025] |  |
| ADL |  |  | 0.011 |  | 0.009 |  |
|  |  |  | [-0.007 0.029] |  | [-0.006 0.024] |  |
| chron |  |  | 0.117 | *** | 0.099 | *** |
|  |  |  | [0.090 0.145] |  | [0.075 0.124] |  |
| agegroup1 | |  |  |  | 0.032 | * |
|  |  |  |  |  | [0.007 0.057] |  |
| agegroup3 | |  |  |  | 0.020 | *** |
|  |  |  |  |  | [0.010 0.029] |  |
| female |  |  |  |  | -0.004 |  |
|  |  |  |  |  | [-0.009 0.001] |  |
| partnerinhh== 3.0000 | | |  |  | 0.002 |  |
|  |  |  |  |  | [-0.004 0.008] |  |
| bieduc |  |  |  |  | 0.015 | ** |
|  |  |  |  |  | [0.005 0.025] |  |
| employment2 | |  |  |  | -0.018 | ** |
|  |  |  |  |  | [-0.031 -0.006] |  |
| employment3 | |  |  |  | 0.000 |  |
|  |  |  |  |  | [-0.004 0.005] |  |
| employment4 | |  |  |  | 0.063 | *** |
|  |  |  |  |  | [0.034 0.091] |  |
| RECODE of hin_adjlog (hin_adjlog) | | | |  | 0.001 |  |
|  |  |  |  |  | [-0.003 0.004] |  |
| housing==not tenant | |  |  |  | -0.001 |  |
|  |  |  |  |  | [-0.004 0.002] |  |
| EUROD | -0.066 |  | -0.058 |  | -0.087 | * |
|  | [-0.140 0.009] |  | [-0.129 0.013] |  | [-0.158 -0.016] |  |
| ADL |  |  | 0.012 |  | 0.007 |  |
|  |  |  | [-0.011 0.036] |  | [-0.018 0.032] |  |
| chron |  |  | -0.040 |  | -0.049 |  |
|  |  |  | [-0.177 0.098] |  | [-0.179 0.080] |  |
| agegroup1 | |  |  |  | -0.002 |  |
|  |  |  |  |  | [-0.063 0.059] |  |
| agegroup3 | |  |  |  | -0.032 |  |
|  |  |  |  |  | [-0.078 0.014] |  |
| female |  |  |  |  | 0.149 | ** |
|  |  |  |  |  | [0.047 0.251] |  |
| partnerinhh== 3.0000 | | |  |  | 0.050 |  |
|  |  |  |  |  | [-0.014 0.115] |  |
| bieduc |  |  |  |  | 0.016 |  |
|  |  |  |  |  | [-0.019 0.051] |  |
| employment2 | |  |  |  | 0.003 |  |
|  |  |  |  |  | [-0.008 0.015] |  |
| employment3 | |  |  |  | 0.027 |  |
|  |  |  |  |  | [-0.005 0.060] |  |
| employment4 | |  |  |  | 0.138 |  |
|  |  |  |  |  | [-0.025 0.300] |  |
| RECODE of hin_adjlog (hin_adjlog) | | | |  | -0.006 |  |
|  |  |  |  |  | [-0.408 0.395] |  |
| housing==not tenant | |  |  |  | 0.027 |  |
|  |  |  |  |  | [-0.009 0.063] |  |
| Intercept | 0.238 | *** | 0.111 |  | -0.307 |  |
|  | [0.134 0.342] |  | [-0.048 0.270] |  | [-0.826 0.211] |  |
| N | 65860 |  | 65860 |  | 65860 |  |
| *** p<.001, ** p<.01, * p<.05 | | |  |  |  |  |

### Czech Republic

| Oaxaca decomposition | | |  |  |  |  |
| --- | --- | --- | --- | --- | --- | --- |
|  | srh | | srh | | srh | |
| group_1 | 2.961 | *** | 2.961 | *** | 2.961 | *** |
|  | [2.758 3.164] |  | [2.801 3.121] |  | [2.806 3.116] |  |
| group_2 | 3.103 | *** | 3.103 | *** | 3.103 | *** |
|  | [3.062 3.144] |  | [3.062 3.144] |  | [3.062 3.144] |  |
| difference | -0.142 |  | -0.142 |  | -0.142 |  |
|  | [-0.349 0.065] |  | [-0.307 0.023] |  | [-0.302 0.018] |  |
| explained | 0.040 |  | 0.074 | * | 0.050 |  |
|  | [-0.023 0.104] |  | [0.012 0.136] |  | [-0.008 0.109] |  |
| unexplained | -0.182 |  | -0.216 | ** | -0.192 | * |
|  | [-0.389 0.024] |  | [-0.367 -0.064] |  | [-0.345 -0.040] |  |
| EUROD | 0.040 |  | 0.030 |  | 0.029 |  |
|  | [-0.023 0.104] |  | [-0.017 0.078] |  | [-0.017 0.074] |  |
| ADL |  |  | -0.004 |  | -0.004 |  |
|  |  |  | [-0.022 0.014] |  | [-0.019 0.012] |  |
| chron |  |  | 0.048 |  | 0.040 |  |
|  |  |  | [-0.003 0.099] |  | [-0.003 0.084] |  |
| agegroup1 | |  |  |  | 0.002 |  |
|  |  |  |  |  | [-0.004 0.009] |  |
| agegroup3 | |  |  |  | -0.003 | * |
|  |  |  |  |  | [-0.005 -0.000] |  |
| female |  |  |  |  | -0.006 |  |
|  |  |  |  |  | [-0.015 0.003] |  |
| partnerinhh== 3.0000 | | |  |  | 0.001 |  |
|  |  |  |  |  | [-0.003 0.005] |  |
| bieduc |  |  |  |  | 0.020 | * |
|  |  |  |  |  | [0.003 0.036] |  |
| employment2 | |  |  |  | -0.013 | * |
|  |  |  |  |  | [-0.024 -0.002] |  |
| employment3 | |  |  |  | -0.017 | * |
|  |  |  |  |  | [-0.031 -0.004] |  |
| employment4 | |  |  |  | 0.018 |  |
|  |  |  |  |  | [-0.004 0.040] |  |
| RECODE of hin_adjlog (hin_adjlog) | | | |  | -0.002 |  |
|  |  |  |  |  | [-0.013 0.009] |  |
| housing==not tenant | |  |  |  | -0.015 |  |
|  |  |  |  |  | [-0.037 0.008] |  |
| EUROD | -0.246 | * | -0.160 | * | -0.110 |  |
|  | [-0.471 -0.022] |  | [-0.299 -0.021] |  | [-0.236 0.015] |  |
| ADL |  |  | -0.012 |  | -0.009 |  |
|  |  |  | [-0.041 0.017] |  | [-0.031 0.014] |  |
| chron |  |  | 0.184 |  | 0.167 |  |
|  |  |  | [-0.001 0.368] |  | [-0.035 0.369] |  |
| agegroup1 | |  |  |  | 0.126 |  |
|  |  |  |  |  | [-0.001 0.254] |  |
| agegroup3 | |  |  |  | 0.001 |  |
|  |  |  |  |  | [-0.006 0.009] |  |
| female |  |  |  |  | 0.019 |  |
|  |  |  |  |  | [-0.112 0.151] |  |
| partnerinhh== 3.0000 | | |  |  | 0.087 |  |
|  |  |  |  |  | [-0.029 0.204] |  |
| bieduc |  |  |  |  | 0.006 |  |
|  |  |  |  |  | [-0.046 0.058] |  |
| employment2 | |  |  |  | -0.006 |  |
|  |  |  |  |  | [-0.022 0.011] |  |
| employment3 | |  |  |  | 0.001 |  |
|  |  |  |  |  | [-0.003 0.005] |  |
| employment4 | |  |  |  | 0.007 |  |
|  |  |  |  |  | [-0.156 0.169] |  |
| RECODE of hin_adjlog (hin_adjlog) | | | |  | -0.005 |  |
|  |  |  |  |  | [-0.624 0.613] |  |
| housing==not tenant | |  |  |  | -0.053 |  |
|  |  |  |  |  | [-0.135 0.029] |  |
| Intercept | 0.064 |  | -0.227 | ** | -0.424 |  |
|  | [-0.143 0.271] |  | [-0.372 -0.082] |  | [-1.099 0.251] |  |
| N | 68249 |  | 68249 |  | 68249 |  |
| *** p<.001, ** p<.01, * p<.05 | | |  |  |  |  |

### Denmark

| Oaxaca decomposition | | |  |  |  |  |
| --- | --- | --- | --- | --- | --- | --- |
|  | srh | | srh | | srh | |
| group_1 | 2.636 | *** | 2.636 | *** | 2.636 | *** |
|  | [2.587 2.685] |  | [2.587 2.685] |  | [2.587 2.685] |  |
| group_2 | 3.103 | *** | 3.103 | *** | 3.103 | *** |
|  | [3.062 3.144] |  | [3.062 3.144] |  | [3.062 3.144] |  |
| difference | -0.467 | *** | -0.467 | *** | -0.467 | *** |
|  | [-0.532 -0.403] |  | [-0.531 -0.403] |  | [-0.531 -0.403] |  |
| explained | -0.087 | *** | -0.046 | ** | -0.073 | ** |
|  | [-0.111 -0.063] |  | [-0.078 -0.014] |  | [-0.121 -0.025] |  |
| unexplained | -0.380 | *** | -0.421 | *** | -0.394 | *** |
|  | [-0.439 -0.321] |  | [-0.476 -0.367] |  | [-0.451 -0.338] |  |
| EUROD | -0.087 | *** | -0.065 | *** | -0.062 | *** |
|  | [-0.111 -0.063] |  | [-0.084 -0.047] |  | [-0.080 -0.045] |  |
| ADL |  |  | 0.002 |  | 0.002 |  |
|  |  |  | [-0.008 0.012] |  | [-0.007 0.010] |  |
| chron |  |  | 0.017 | * | 0.015 | * |
|  |  |  | [0.000 0.035] |  | [0.000 0.029] |  |
| agegroup1 | |  |  |  | 0.019 | * |
|  |  |  |  |  | [0.004 0.034] |  |
| agegroup3 | |  |  |  | 0.007 | *** |
|  |  |  |  |  | [0.004 0.011] |  |
| female |  |  |  |  | 0.002 |  |
|  |  |  |  |  | [-0.001 0.005] |  |
| partnerinhh== 3.0000 | | |  |  | 0.001 |  |
|  |  |  |  |  | [-0.002 0.003] |  |
| bieduc |  |  |  |  | -0.055 | *** |
|  |  |  |  |  | [-0.076 -0.034] |  |
| employment2 | |  |  |  | -0.011 | ** |
|  |  |  |  |  | [-0.019 -0.003] |  |
| employment3 | |  |  |  | -0.014 | * |
|  |  |  |  |  | [-0.025 -0.003] |  |
| employment4 | |  |  |  | 0.037 | *** |
|  |  |  |  |  | [0.020 0.054] |  |
| RECODE of hin_adjlog (hin_adjlog) | | | |  | -0.005 |  |
|  |  |  |  |  | [-0.036 0.026] |  |
| housing==not tenant | |  |  |  | -0.007 |  |
|  |  |  |  |  | [-0.019 0.004] |  |
| EUROD | 0.151 | *** | 0.153 | *** | 0.146 | *** |
|  | [0.096 0.207] |  | [0.097 0.208] |  | [0.090 0.202] |  |
| ADL |  |  | 0.026 | ** | 0.022 | * |
|  |  |  | [0.009 0.042] |  | [0.005 0.038] |  |
| chron |  |  | -0.064 |  | -0.088 | * |
|  |  |  | [-0.131 0.003] |  | [-0.156 -0.020] |  |
| agegroup1 | |  |  |  | 0.043 |  |
|  |  |  |  |  | [-0.028 0.113] |  |
| agegroup3 | |  |  |  | -0.003 |  |
|  |  |  |  |  | [-0.024 0.018] |  |
| female |  |  |  |  | -0.015 |  |
|  |  |  |  |  | [-0.076 0.046] |  |
| partnerinhh== 3.0000 | | |  |  | -0.002 |  |
|  |  |  |  |  | [-0.051 0.047] |  |
| bieduc |  |  |  |  | 0.032 |  |
|  |  |  |  |  | [-0.026 0.090] |  |
| employment2 | |  |  |  | 0.015 | * |
|  |  |  |  |  | [0.001 0.028] |  |
| employment3 | |  |  |  | 0.007 |  |
|  |  |  |  |  | [-0.001 0.016] |  |
| employment4 | |  |  |  | -0.011 |  |
|  |  |  |  |  | [-0.107 0.085] |  |
| RECODE of hin_adjlog (hin_adjlog) | | | |  | -1.901 | *** |
|  |  |  |  |  | [-2.837 -0.966] |  |
| housing==not tenant | |  |  |  | 0.045 | * |
|  |  |  |  |  | [0.005 0.085] |  |
| Intercept | -0.531 | *** | -0.536 | *** | 1.316 | ** |
|  | [-0.610 -0.453] |  | [-0.628 -0.443] |  | [0.333 2.300] |  |
| N | 67450 |  | 67450 |  | 67450 |  |
| *** p<.001, ** p<.01, * p<.05 | | |  |  |  |  |

### Estonia

| Oaxaca decomposition | | |  |  |  |  |
| --- | --- | --- | --- | --- | --- | --- |
|  | srh | | srh | | srh | |
| group_1 | 3.718 | *** | 3.718 | *** | 3.718 | *** |
|  | [3.689 3.747] |  | [3.689 3.747] |  | [3.689 3.747] |  |
| group_2 | 3.103 | *** | 3.103 | *** | 3.103 | *** |
|  | [3.062 3.144] |  | [3.062 3.144] |  | [3.062 3.144] |  |
| difference | 0.615 | *** | 0.615 | *** | 0.615 | *** |
|  | [0.564 0.665] |  | [0.565 0.665] |  | [0.565 0.665] |  |
| explained | 0.095 | *** | 0.170 | *** | 0.144 | *** |
|  | [0.072 0.118] |  | [0.140 0.200] |  | [0.105 0.183] |  |
| unexplained | 0.519 | *** | 0.445 | *** | 0.470 | *** |
|  | [0.473 0.566] |  | [0.403 0.487] |  | [0.427 0.514] |  |
| EUROD | 0.095 | *** | 0.071 | *** | 0.068 | *** |
|  | [0.072 0.118] |  | [0.053 0.089] |  | [0.051 0.085] |  |
| ADL |  |  | 0.027 | *** | 0.023 | *** |
|  |  |  | [0.017 0.038] |  | [0.014 0.032] |  |
| chron |  |  | 0.071 | *** | 0.060 | *** |
|  |  |  | [0.054 0.088] |  | [0.045 0.076] |  |
| agegroup1 | |  |  |  | 0.022 | * |
|  |  |  |  |  | [0.005 0.039] |  |
| agegroup3 | |  |  |  | 0.016 | *** |
|  |  |  |  |  | [0.009 0.023] |  |
| female |  |  |  |  | -0.009 | * |
|  |  |  |  |  | [-0.016 -0.001] |  |
| partnerinhh== 3.0000 | | |  |  | -0.005 |  |
|  |  |  |  |  | [-0.018 0.008] |  |
| bieduc |  |  |  |  | -0.044 | *** |
|  |  |  |  |  | [-0.061 -0.027] |  |
| employment2 | |  |  |  | -0.009 | * |
|  |  |  |  |  | [-0.016 -0.002] |  |
| employment3 | |  |  |  | -0.017 | * |
|  |  |  |  |  | [-0.030 -0.004] |  |
| employment4 | |  |  |  | 0.041 | *** |
|  |  |  |  |  | [0.023 0.060] |  |
| RECODE of hin_adjlog (hin_adjlog) | | | |  | -0.001 |  |
|  |  |  |  |  | [-0.005 0.004] |  |
| housing==not tenant | |  |  |  | -0.002 |  |
|  |  |  |  |  | [-0.005 0.001] |  |
| EUROD | -0.069 | * | -0.067 | * | -0.093 | ** |
|  | [-0.123 -0.015] |  | [-0.124 -0.010] |  | [-0.150 -0.037] |  |
| ADL |  |  | -0.016 |  | -0.016 |  |
|  |  |  | [-0.033 0.001] |  | [-0.032 0.001] |  |
| chron |  |  | -0.020 |  | -0.050 |  |
|  |  |  | [-0.085 0.045] |  | [-0.115 0.014] |  |
| agegroup1 | |  |  |  | -0.030 |  |
|  |  |  |  |  | [-0.073 0.012] |  |
| agegroup3 | |  |  |  | 0.002 |  |
|  |  |  |  |  | [-0.015 0.019] |  |
| female |  |  |  |  | -0.004 |  |
|  |  |  |  |  | [-0.064 0.057] |  |
| partnerinhh== 3.0000 | | |  |  | 0.003 |  |
|  |  |  |  |  | [-0.052 0.057] |  |
| bieduc |  |  |  |  | 0.048 | * |
|  |  |  |  |  | [0.006 0.090] |  |
| employment2 | |  |  |  | 0.017 | ** |
|  |  |  |  |  | [0.005 0.029] |  |
| employment3 | |  |  |  | -0.001 |  |
|  |  |  |  |  | [-0.005 0.002] |  |
| employment4 | |  |  |  | 0.025 |  |
|  |  |  |  |  | [-0.044 0.094] |  |
| RECODE of hin_adjlog (hin_adjlog) | | | |  | -0.302 |  |
|  |  |  |  |  | [-0.668 0.065] |  |
| housing==not tenant | |  |  |  | 0.018 |  |
|  |  |  |  |  | [-0.004 0.041] |  |
| Intercept | 0.588 | *** | 0.548 | *** | 0.853 | *** |
|  | [0.520 0.657] |  | [0.463 0.632] |  | [0.429 1.277] |  |
| N | 69408 |  | 69408 |  | 69408 |  |
| *** p<.001, ** p<.01, * p<.05 | | |  |  |  |  |

### Finland

| Oaxaca decomposition | | |  |  |  |  |
| --- | --- | --- | --- | --- | --- | --- |
|  | srh | | srh | | srh | |
| group_1 | 3.145 | *** | 3.145 | *** | 3.145 | *** |
|  | [3.084 3.206] |  | [3.083 3.206] |  | [3.085 3.205] |  |
| group_2 | 3.103 | *** | 3.103 | *** | 3.103 | *** |
|  | [3.062 3.144] |  | [3.062 3.144] |  | [3.062 3.144] |  |
| difference | 0.042 |  | 0.042 |  | 0.042 |  |
|  | [-0.032 0.115] |  | [-0.032 0.116] |  | [-0.031 0.114] |  |
| explained | -0.030 | * | 0.070 | *** | 0.075 | ** |
|  | [-0.056 -0.004] |  | [0.032 0.108] |  | [0.024 0.127] |  |
| unexplained | 0.072 | * | -0.028 |  | -0.033 |  |
|  | [0.003 0.141] |  | [-0.092 0.035] |  | [-0.093 0.026] |  |
| EUROD | -0.030 | * | -0.023 | * | -0.022 | * |
|  | [-0.056 -0.004] |  | [-0.042 -0.003] |  | [-0.040 -0.003] |  |
| ADL |  |  | 0.005 |  | 0.004 |  |
|  |  |  | [-0.007 0.017] |  | [-0.006 0.015] |  |
| chron |  |  | 0.087 | *** | 0.074 | *** |
|  |  |  | [0.064 0.110] |  | [0.053 0.094] |  |
| agegroup1 | |  |  |  | 0.027 | * |
|  |  |  |  |  | [0.006 0.048] |  |
| agegroup3 | |  |  |  | 0.011 | *** |
|  |  |  |  |  | [0.006 0.016] |  |
| female |  |  |  |  | 0.002 |  |
|  |  |  |  |  | [-0.002 0.005] |  |
| partnerinhh== 3.0000 | | |  |  | -0.003 |  |
|  |  |  |  |  | [-0.009 0.004] |  |
| bieduc |  |  |  |  | -0.050 | *** |
|  |  |  |  |  | [-0.069 -0.030] |  |
| employment2 | |  |  |  | -0.019 | ** |
|  |  |  |  |  | [-0.031 -0.006] |  |
| employment3 | |  |  |  | -0.017 | * |
|  |  |  |  |  | [-0.031 -0.004] |  |
| employment4 | |  |  |  | 0.074 | *** |
|  |  |  |  |  | [0.041 0.107] |  |
| RECODE of hin_adjlog (hin_adjlog) | | | |  | -0.004 |  |
|  |  |  |  |  | [-0.027 0.019] |  |
| housing==not tenant | |  |  |  | -0.003 |  |
|  |  |  |  |  | [-0.008 0.002] |  |
| EUROD | -0.016 |  | -0.004 |  | -0.004 |  |
|  | [-0.095 0.062] |  | [-0.080 0.072] |  | [-0.074 0.065] |  |
| ADL |  |  | 0.014 |  | 0.008 |  |
|  |  |  | [-0.002 0.030] |  | [-0.008 0.024] |  |
| chron |  |  | 0.017 |  | -0.001 |  |
|  |  |  | [-0.086 0.120] |  | [-0.099 0.097] |  |
| agegroup1 | |  |  |  | 0.027 |  |
|  |  |  |  |  | [-0.029 0.083] |  |
| agegroup3 | |  |  |  | 0.015 |  |
|  |  |  |  |  | [-0.010 0.040] |  |
| female |  |  |  |  | -0.026 |  |
|  |  |  |  |  | [-0.094 0.042] |  |
| partnerinhh== 3.0000 | | |  |  | -0.007 |  |
|  |  |  |  |  | [-0.075 0.062] |  |
| bieduc |  |  |  |  | 0.009 |  |
|  |  |  |  |  | [-0.052 0.069] |  |
| employment2 | |  |  |  | -0.002 |  |
|  |  |  |  |  | [-0.009 0.005] |  |
| employment3 | |  |  |  | -0.005 |  |
|  |  |  |  |  | [-0.011 0.001] |  |
| employment4 | |  |  |  | 0.079 |  |
|  |  |  |  |  | [-0.064 0.221] |  |
| RECODE of hin_adjlog (hin_adjlog) | | | |  | -0.898 | ** |
|  |  |  |  |  | [-1.525 -0.271] |  |
| housing==not tenant | |  |  |  | 0.043 | ** |
|  |  |  |  |  | [0.011 0.076] |  |
| Intercept | 0.088 |  | -0.055 |  | 0.728 | * |
|  | [-0.022 0.199] |  | [-0.192 0.082] |  | [0.035 1.422] |  |
| N | 66875 |  | 66875 |  | 66875 |  |
| *** p<.001, ** p<.01, * p<.05 | | |  |  |  |  |

### France

| Oaxaca decomposition | | |  |  |  |  |
| --- | --- | --- | --- | --- | --- | --- |
|  | srh | | srh | | srh | |
| group_1 | 3.038 | *** | 3.038 | *** | 3.038 | *** |
|  | [2.991 3.085] |  | [2.991 3.085] |  | [2.991 3.085] |  |
| group_2 | 3.103 | *** | 3.103 | *** | 3.103 | *** |
|  | [3.062 3.144] |  | [3.062 3.144] |  | [3.062 3.144] |  |
| difference | -0.065 | * | -0.065 | * | -0.065 | * |
|  | [-0.128 -0.003] |  | [-0.127 -0.003] |  | [-0.127 -0.003] |  |
| explained | 0.049 | *** | 0.033 | * | 0.039 |  |
|  | [0.024 0.073] |  | [0.001 0.065] |  | [-0.004 0.083] |  |
| unexplained | -0.114 | *** | -0.098 | *** | -0.104 | *** |
|  | [-0.172 -0.056] |  | [-0.152 -0.044] |  | [-0.158 -0.051] |  |
| EUROD | 0.049 | *** | 0.036 | *** | 0.035 | *** |
|  | [0.024 0.073] |  | [0.018 0.055] |  | [0.017 0.053] |  |
| ADL |  |  | 0.004 |  | 0.003 |  |
|  |  |  | [-0.006 0.013] |  | [-0.005 0.011] |  |
| chron |  |  | -0.007 |  | -0.006 |  |
|  |  |  | [-0.025 0.011] |  | [-0.021 0.009] |  |
| agegroup1 | |  |  |  | 0.006 | * |
|  |  |  |  |  | [0.001 0.012] |  |
| agegroup3 | |  |  |  | 0.002 |  |
|  |  |  |  |  | [-0.000 0.003] |  |
| female |  |  |  |  | 0.002 |  |
|  |  |  |  |  | [-0.001 0.006] |  |
| partnerinhh== 3.0000 | | |  |  | 0.003 |  |
|  |  |  |  |  | [-0.004 0.009] |  |
| bieduc |  |  |  |  | -0.014 | *** |
|  |  |  |  |  | [-0.023 -0.006] |  |
| employment2 | |  |  |  | -0.002 |  |
|  |  |  |  |  | [-0.008 0.003] |  |
| employment3 | |  |  |  | -0.013 | * |
|  |  |  |  |  | [-0.024 -0.003] |  |
| employment4 | |  |  |  | 0.033 | *** |
|  |  |  |  |  | [0.018 0.048] |  |
| RECODE of hin_adjlog (hin_adjlog) | | | |  | -0.005 |  |
|  |  |  |  |  | [-0.033 0.023] |  |
| housing==not tenant | |  |  |  | -0.004 |  |
|  |  |  |  |  | [-0.010 0.003] |  |
| EUROD | -0.007 |  | 0.048 |  | 0.050 |  |
|  | [-0.074 0.059] |  | [-0.018 0.114] |  | [-0.016 0.117] |  |
| ADL |  |  | 0.001 |  | 0.000 |  |
|  |  |  | [-0.014 0.016] |  | [-0.015 0.014] |  |
| chron |  |  | -0.048 |  | -0.034 |  |
|  |  |  | [-0.110 0.014] |  | [-0.096 0.029] |  |
| agegroup1 | |  |  |  | -0.032 |  |
|  |  |  |  |  | [-0.103 0.038] |  |
| agegroup3 | |  |  |  | -0.007 |  |
|  |  |  |  |  | [-0.019 0.004] |  |
| female |  |  |  |  | 0.027 |  |
|  |  |  |  |  | [-0.033 0.087] |  |
| partnerinhh== 3.0000 | | |  |  | -0.017 |  |
|  |  |  |  |  | [-0.057 0.023] |  |
| bieduc |  |  |  |  | 0.019 |  |
|  |  |  |  |  | [-0.020 0.057] |  |
| employment2 | |  |  |  | 0.011 |  |
|  |  |  |  |  | [-0.010 0.032] |  |
| employment3 | |  |  |  | -0.008 |  |
|  |  |  |  |  | [-0.018 0.002] |  |
| employment4 | |  |  |  | -0.042 |  |
|  |  |  |  |  | [-0.123 0.039] |  |
| RECODE of hin_adjlog (hin_adjlog) | | | |  | -0.800 | ** |
|  |  |  |  |  | [-1.341 -0.258] |  |
| housing==not tenant | |  |  |  | 0.044 | ** |
|  |  |  |  |  | [0.012 0.077] |  |
| Intercept | -0.107 | * | -0.099 |  | 0.684 | * |
|  | [-0.195 -0.019] |  | [-0.201 0.003] |  | [0.099 1.269] |  |
| N | 67919 |  | 67919 |  | 67919 |  |
| *** p<.001, ** p<.01, * p<.05 | | |  |  |  |  |

### Germany

| Oaxaca decomposition | | |  |  |  |  |
| --- | --- | --- | --- | --- | --- | --- |
|  | srh | | srh | | srh | |
| group_1 | 3.151 | *** | 3.151 | *** | 3.151 | *** |
|  | [3.112 3.190] |  | [3.113 3.189] |  | [3.113 3.189] |  |
| group_2 | 3.103 | *** | 3.103 | *** | 3.103 | *** |
|  | [3.062 3.144] |  | [3.062 3.144] |  | [3.062 3.144] |  |
| difference | 0.048 |  | 0.048 |  | 0.048 |  |
|  | [-0.009 0.104] |  | [-0.008 0.104] |  | [-0.008 0.103] |  |
| explained | 0.014 |  | 0.094 | *** | 0.047 |  |
|  | [-0.008 0.036] |  | [0.063 0.124] |  | [-0.001 0.096] |  |
| unexplained | 0.034 |  | -0.046 |  | 0.001 |  |
|  | [-0.019 0.086] |  | [-0.093 0.001] |  | [-0.052 0.053] |  |
| EUROD | 0.014 |  | 0.011 |  | 0.010 |  |
|  | [-0.008 0.036] |  | [-0.006 0.027] |  | [-0.005 0.026] |  |
| ADL |  |  | 0.017 | ** | 0.014 | ** |
|  |  |  | [0.007 0.027] |  | [0.005 0.022] |  |
| chron |  |  | 0.066 | *** | 0.056 | *** |
|  |  |  | [0.049 0.084] |  | [0.041 0.072] |  |
| agegroup1 | |  |  |  | 0.012 | * |
|  |  |  |  |  | [0.003 0.021] |  |
| agegroup3 | |  |  |  | 0.005 | *** |
|  |  |  |  |  | [0.003 0.008] |  |
| female |  |  |  |  | 0.003 |  |
|  |  |  |  |  | [-0.000 0.006] |  |
| partnerinhh== 3.0000 | | |  |  | 0.002 |  |
|  |  |  |  |  | [-0.003 0.006] |  |
| bieduc |  |  |  |  | -0.042 | *** |
|  |  |  |  |  | [-0.057 -0.027] |  |
| employment2 | |  |  |  | -0.007 | * |
|  |  |  |  |  | [-0.013 -0.001] |  |
| employment3 | |  |  |  | -0.012 | * |
|  |  |  |  |  | [-0.022 -0.003] |  |
| employment4 | |  |  |  | 0.027 | *** |
|  |  |  |  |  | [0.014 0.040] |  |
| RECODE of hin_adjlog (hin_adjlog) | | | |  | -0.006 |  |
|  |  |  |  |  | [-0.037 0.025] |  |
| housing==not tenant | |  |  |  | -0.014 |  |
|  |  |  |  |  | [-0.037 0.009] |  |
| EUROD | -0.012 |  | 0.001 |  | -0.006 |  |
|  | [-0.069 0.046] |  | [-0.057 0.059] |  | [-0.064 0.052] |  |
| ADL |  |  | 0.020 | * | 0.017 | * |
|  |  |  | [0.003 0.037] |  | [0.001 0.033] |  |
| chron |  |  | -0.012 |  | -0.027 |  |
|  |  |  | [-0.082 0.059] |  | [-0.096 0.041] |  |
| agegroup1 | |  |  |  | 0.000 |  |
|  |  |  |  |  | [-0.061 0.060] |  |
| agegroup3 | |  |  |  | 0.005 |  |
|  |  |  |  |  | [-0.007 0.017] |  |
| female |  |  |  |  | -0.006 |  |
|  |  |  |  |  | [-0.058 0.045] |  |
| partnerinhh== 3.0000 | | |  |  | -0.014 |  |
|  |  |  |  |  | [-0.053 0.025] |  |
| bieduc |  |  |  |  | 0.005 |  |
|  |  |  |  |  | [-0.040 0.050] |  |
| employment2 | |  |  |  | 0.016 | * |
|  |  |  |  |  | [0.001 0.031] |  |
| employment3 | |  |  |  | -0.009 |  |
|  |  |  |  |  | [-0.019 0.001] |  |
| employment4 | |  |  |  | -0.024 |  |
|  |  |  |  |  | [-0.094 0.045] |  |
| RECODE of hin_adjlog (hin_adjlog) | | | |  | -1.236 | *** |
|  |  |  |  |  | [-1.830 -0.642] |  |
| housing==not tenant | |  |  |  | 0.022 |  |
|  |  |  |  |  | [-0.024 0.069] |  |
| Intercept | 0.045 |  | -0.055 |  | 1.259 | *** |
|  | [-0.031 0.121] |  | [-0.147 0.037] |  | [0.639 1.878] |  |
| N | 69500 |  | 69500 |  | 69500 |  |
| *** p<.001, ** p<.01, * p<.05 | | |  |  |  |  |

### Greece

| Oaxaca decomposition | | |  |  |  |  |
| --- | --- | --- | --- | --- | --- | --- |
|  | srh | | srh | | srh | |
| group_1 | 2.974 | *** | 2.974 | *** | 2.974 | *** |
|  | [2.934 3.014] |  | [2.935 3.014] |  | [2.935 3.014] |  |
| group_2 | 3.103 | *** | 3.103 | *** | 3.103 | *** |
|  | [3.062 3.144] |  | [3.062 3.144] |  | [3.062 3.144] |  |
| difference | -0.129 | *** | -0.129 | *** | -0.129 | *** |
|  | [-0.186 -0.071] |  | [-0.186 -0.072] |  | [-0.186 -0.072] |  |
| explained | -0.142 | *** | -0.014 |  | 0.022 |  |
|  | [-0.169 -0.115] |  | [-0.050 0.022] |  | [-0.021 0.065] |  |
| unexplained | 0.014 |  | -0.115 | *** | -0.151 | *** |
|  | [-0.041 0.068] |  | [-0.164 -0.065] |  | [-0.196 -0.105] |  |
| EUROD | -0.142 | *** | -0.106 | *** | -0.102 | *** |
|  | [-0.169 -0.115] |  | [-0.128 -0.084] |  | [-0.123 -0.080] |  |
| ADL |  |  | -0.020 | *** | -0.016 | *** |
|  |  |  | [-0.028 -0.011] |  | [-0.024 -0.009] |  |
| chron |  |  | 0.112 | *** | 0.094 | *** |
|  |  |  | [0.090 0.133] |  | [0.075 0.114] |  |
| agegroup1 | |  |  |  | 0.014 | ** |
|  |  |  |  |  | [0.004 0.025] |  |
| agegroup3 | |  |  |  | 0.005 | *** |
|  |  |  |  |  | [0.003 0.008] |  |
| female |  |  |  |  | 0.000 |  |
|  |  |  |  |  | [-0.002 0.003] |  |
| partnerinhh== 3.0000 | | |  |  | 0.004 |  |
|  |  |  |  |  | [-0.007 0.016] |  |
| bieduc |  |  |  |  | -0.003 |  |
|  |  |  |  |  | [-0.009 0.003] |  |
| employment2 | |  |  |  | -0.016 | ** |
|  |  |  |  |  | [-0.027 -0.005] |  |
| employment3 | |  |  |  | 0.024 | * |
|  |  |  |  |  | [0.005 0.043] |  |
| employment4 | |  |  |  | 0.014 | ** |
|  |  |  |  |  | [0.005 0.022] |  |
| RECODE of hin_adjlog (hin_adjlog) | | | |  | 0.001 |  |
|  |  |  |  |  | [-0.005 0.007] |  |
| housing==not tenant | |  |  |  | 0.002 |  |
|  |  |  |  |  | [-0.001 0.006] |  |
| EUROD | -0.054 | * | -0.031 |  | -0.040 |  |
|  | [-0.096 -0.012] |  | [-0.074 0.012] |  | [-0.083 0.003] |  |
| ADL |  |  | 0.002 |  | -0.004 |  |
|  |  |  | [-0.008 0.012] |  | [-0.014 0.005] |  |
| chron |  |  | 0.163 | *** | 0.095 | * |
|  |  |  | [0.079 0.248] |  | [0.010 0.180] |  |
| agegroup1 | |  |  |  | -0.148 | *** |
|  |  |  |  |  | [-0.205 -0.091] |  |
| agegroup3 | |  |  |  | 0.021 | ** |
|  |  |  |  |  | [0.008 0.034] |  |
| female |  |  |  |  | 0.040 |  |
|  |  |  |  |  | [-0.022 0.102] |  |
| partnerinhh== 3.0000 | | |  |  | 0.016 |  |
|  |  |  |  |  | [-0.014 0.046] |  |
| bieduc |  |  |  |  | 0.034 | * |
|  |  |  |  |  | [0.004 0.065] |  |
| employment2 | |  |  |  | 0.001 |  |
|  |  |  |  |  | [-0.008 0.010] |  |
| employment3 | |  |  |  | -0.008 |  |
|  |  |  |  |  | [-0.053 0.037] |  |
| employment4 | |  |  |  | -0.055 |  |
|  |  |  |  |  | [-0.120 0.010] |  |
| RECODE of hin_adjlog (hin_adjlog) | | | |  | 0.187 |  |
|  |  |  |  |  | [-0.116 0.490] |  |
| housing==not tenant | |  |  |  | 0.009 |  |
|  |  |  |  |  | [-0.009 0.027] |  |
| Intercept | 0.067 |  | -0.250 | *** | -0.299 |  |
|  | [-0.000 0.135] |  | [-0.345 -0.155] |  | [-0.674 0.075] |  |
| N | 68109 |  | 68109 |  | 68109 |  |
| *** p<.001, ** p<.01, * p<.05 | | |  |  |  |  |

### Hungary

| Oaxaca decomposition | | |  |  |  |  |
| --- | --- | --- | --- | --- | --- | --- |
|  | srh | | srh | | srh | |
| group_1 | 3.022 | *** | 3.022 | *** | 3.022 | *** |
|  | [2.966 3.078] |  | [2.965 3.079] |  | [2.965 3.079] |  |
| group_2 | 3.103 | *** | 3.103 | *** | 3.103 | *** |
|  | [3.062 3.144] |  | [3.062 3.144] |  | [3.062 3.144] |  |
| difference | -0.081 | * | -0.081 | * | -0.081 | * |
|  | [-0.151 -0.011] |  | [-0.151 -0.011] |  | [-0.152 -0.011] |  |
| explained | -0.122 | *** | -0.068 | ** | -0.042 |  |
|  | [-0.152 -0.092] |  | [-0.109 -0.027] |  | [-0.091 0.007] |  |
| unexplained | 0.041 |  | -0.013 |  | -0.039 |  |
|  | [-0.022 0.104] |  | [-0.071 0.045] |  | [-0.100 0.022] |  |
| EUROD | -0.122 | *** | -0.091 | *** | -0.087 | *** |
|  | [-0.152 -0.092] |  | [-0.115 -0.067] |  | [-0.110 -0.064] |  |
| ADL |  |  | -0.009 |  | -0.007 |  |
|  |  |  | [-0.019 0.002] |  | [-0.016 0.002] |  |
| chron |  |  | 0.032 | ** | 0.027 | ** |
|  |  |  | [0.010 0.053] |  | [0.009 0.045] |  |
| agegroup1 | |  |  |  | 0.008 | * |
|  |  |  |  |  | [0.001 0.014] |  |
| agegroup3 | |  |  |  | -0.001 |  |
|  |  |  |  |  | [-0.003 0.001] |  |
| female |  |  |  |  | -0.001 |  |
|  |  |  |  |  | [-0.004 0.003] |  |
| partnerinhh== 3.0000 | | |  |  | 0.000 |  |
|  |  |  |  |  | [-0.002 0.001] |  |
| bieduc |  |  |  |  | 0.012 | ** |
|  |  |  |  |  | [0.004 0.020] |  |
| employment2 | |  |  |  | -0.015 | ** |
|  |  |  |  |  | [-0.025 -0.004] |  |
| employment3 | |  |  |  | -0.017 | * |
|  |  |  |  |  | [-0.031 -0.004] |  |
| employment4 | |  |  |  | 0.031 | *** |
|  |  |  |  |  | [0.016 0.046] |  |
| RECODE of hin_adjlog (hin_adjlog) | | | |  | 0.002 |  |
|  |  |  |  |  | [-0.008 0.012] |  |
| housing==not tenant | |  |  |  | 0.006 |  |
|  |  |  |  |  | [-0.004 0.017] |  |
| EUROD | 0.046 |  | 0.047 |  | 0.019 |  |
|  | [-0.001 0.093] |  | [-0.001 0.095] |  | [-0.029 0.068] |  |
| ADL |  |  | -0.006 |  | -0.002 |  |
|  |  |  | [-0.020 0.008] |  | [-0.015 0.012] |  |
| chron |  |  | -0.065 |  | -0.059 |  |
|  |  |  | [-0.143 0.013] |  | [-0.139 0.022] |  |
| agegroup1 | |  |  |  | 0.057 |  |
|  |  |  |  |  | [-0.033 0.147] |  |
| agegroup3 | |  |  |  | 0.000 |  |
|  |  |  |  |  | [-0.014 0.014] |  |
| female |  |  |  |  | 0.039 |  |
|  |  |  |  |  | [-0.033 0.110] |  |
| partnerinhh== 3.0000 | | |  |  | -0.033 |  |
|  |  |  |  |  | [-0.092 0.026] |  |
| bieduc |  |  |  |  | 0.013 |  |
|  |  |  |  |  | [-0.015 0.040] |  |
| employment2 | |  |  |  | 0.004 |  |
|  |  |  |  |  | [-0.010 0.018] |  |
| employment3 | |  |  |  | 0.002 |  |
|  |  |  |  |  | [-0.002 0.006] |  |
| employment4 | |  |  |  | 0.027 |  |
|  |  |  |  |  | [-0.065 0.120] |  |
| RECODE of hin_adjlog (hin_adjlog) | | | |  | -0.218 |  |
|  |  |  |  |  | [-0.709 0.273] |  |
| housing==not tenant | |  |  |  | 0.021 | *** |
|  |  |  |  |  | [0.009 0.034] |  |
| Intercept | -0.005 |  | 0.011 |  | 0.091 |  |
|  | [-0.088 0.078] |  | [-0.098 0.119] |  | [-0.473 0.655] |  |
| N | 66939 |  | 66939 |  | 66939 |  |
| *** p<.001, ** p<.01, * p<.05 | | |  |  |  |  |

### Israel

| Oaxaca decomposition | | |  |  |  |  |
| --- | --- | --- | --- | --- | --- | --- |
|  | srh | | srh | | srh | |
| group_1 | 2.976 | *** | 2.976 | *** | 2.976 | *** |
|  | [2.823 3.129] |  | [2.805 3.147] |  | [2.791 3.162] |  |
| group_2 | 3.103 | *** | 3.103 | *** | 3.103 | *** |
|  | [3.062 3.144] |  | [3.062 3.144] |  | [3.062 3.144] |  |
| difference | -0.127 |  | -0.127 |  | -0.127 |  |
|  | [-0.285 0.031] |  | [-0.303 0.049] |  | [-0.317 0.063] |  |
| explained | 0.008 |  | 0.087 |  | 0.063 |  |
|  | [-0.048 0.064] |  | [-0.007 0.180] |  | [-0.057 0.184] |  |
| unexplained | -0.135 |  | -0.214 | *** | -0.191 | ** |
|  | [-0.275 0.005] |  | [-0.328 -0.100] |  | [-0.305 -0.076] |  |
| EUROD | 0.008 |  | 0.006 |  | 0.006 |  |
|  | [-0.048 0.064] |  | [-0.036 0.048] |  | [-0.034 0.046] |  |
| ADL |  |  | -0.005 |  | -0.004 |  |
|  |  |  | [-0.021 0.011] |  | [-0.018 0.009] |  |
| chron |  |  | 0.086 | ** | 0.073 | ** |
|  |  |  | [0.030 0.141] |  | [0.025 0.120] |  |
| agegroup1 | |  |  |  | 0.034 | * |
|  |  |  |  |  | [0.007 0.061] |  |
| agegroup3 | |  |  |  | 0.013 | *** |
|  |  |  |  |  | [0.005 0.020] |  |
| female |  |  |  |  | 0.000 |  |
|  |  |  |  |  | [-0.007 0.008] |  |
| partnerinhh== 3.0000 | | |  |  | 0.003 |  |
|  |  |  |  |  | [-0.004 0.010] |  |
| bieduc |  |  |  |  | -0.071 | *** |
|  |  |  |  |  | [-0.101 -0.041] |  |
| employment2 | |  |  |  | -0.008 |  |
|  |  |  |  |  | [-0.020 0.004] |  |
| employment3 | |  |  |  | 0.002 |  |
|  |  |  |  |  | [-0.003 0.008] |  |
| employment4 | |  |  |  | 0.023 | * |
|  |  |  |  |  | [0.004 0.042] |  |
| RECODE of hin_adjlog (hin_adjlog) | | | |  | -0.004 |  |
|  |  |  |  |  | [-0.026 0.019] |  |
| housing==not tenant | |  |  |  | -0.002 |  |
|  |  |  |  |  | [-0.007 0.003] |  |
| EUROD | 0.183 | ** | 0.075 |  | 0.037 |  |
|  | [0.069 0.298] |  | [-0.043 0.193] |  | [-0.087 0.160] |  |
| ADL |  |  | 0.015 |  | 0.015 |  |
|  |  |  | [-0.006 0.036] |  | [-0.005 0.036] |  |
| chron |  |  | 0.350 | *** | 0.286 | ** |
|  |  |  | [0.166 0.534] |  | [0.105 0.467] |  |
| agegroup1 | |  |  |  | -0.009 |  |
|  |  |  |  |  | [-0.095 0.078] |  |
| agegroup3 | |  |  |  | -0.007 |  |
|  |  |  |  |  | [-0.049 0.036] |  |
| female |  |  |  |  | -0.040 |  |
|  |  |  |  |  | [-0.164 0.085] |  |
| partnerinhh== 3.0000 | | |  |  | 0.010 |  |
|  |  |  |  |  | [-0.073 0.093] |  |
| bieduc |  |  |  |  | 0.005 |  |
|  |  |  |  |  | [-0.105 0.114] |  |
| employment2 | |  |  |  | 0.017 |  |
|  |  |  |  |  | [-0.029 0.062] |  |
| employment3 | |  |  |  | 0.008 |  |
|  |  |  |  |  | [-0.043 0.058] |  |
| employment4 | |  |  |  | -0.067 |  |
|  |  |  |  |  | [-0.208 0.075] |  |
| RECODE of hin_adjlog (hin_adjlog) | | | |  | -1.972 | * |
|  |  |  |  |  | [-3.756 -0.188] |  |
| housing==not tenant | |  |  |  | -0.018 |  |
|  |  |  |  |  | [-0.076 0.040] |  |
| Intercept | -0.319 | ** | -0.653 | *** | 1.544 |  |
|  | [-0.515 -0.123] |  | [-0.854 -0.453] |  | [-0.332 3.419] |  |
| N | 65765 |  | 65765 |  | 65765 |  |
| *** p<.001, ** p<.01, * p<.05 | | |  |  |  |  |

### Italy

| Oaxaca decomposition | | |  |  |  |  |
| --- | --- | --- | --- | --- | --- | --- |
|  | srh | | srh | | srh | |
| group_1 | 3.148 | *** | 3.148 | *** | 3.148 | *** |
|  | [3.092 3.203] |  | [3.092 3.203] |  | [3.093 3.203] |  |
| group_2 | 3.103 | *** | 3.103 | *** | 3.103 | *** |
|  | [3.062 3.144] |  | [3.062 3.144] |  | [3.062 3.144] |  |
| difference | 0.045 |  | 0.045 |  | 0.045 |  |
|  | [-0.024 0.114] |  | [-0.024 0.114] |  | [-0.024 0.113] |  |
| explained | 0.008 |  | 0.010 |  | 0.072 | *** |
|  | [-0.021 0.037] |  | [-0.026 0.047] |  | [0.031 0.114] |  |
| unexplained | 0.037 |  | 0.034 |  | -0.028 |  |
|  | [-0.027 0.101] |  | [-0.025 0.094] |  | [-0.085 0.029] |  |
| EUROD | 0.008 |  | 0.006 |  | 0.006 |  |
|  | [-0.021 0.037] |  | [-0.015 0.027] |  | [-0.015 0.026] |  |
| ADL |  |  | -0.011 | * | -0.009 | * |
|  |  |  | [-0.021 -0.000] |  | [-0.018 -0.000] |  |
| chron |  |  | 0.015 |  | 0.013 |  |
|  |  |  | [-0.005 0.035] |  | [-0.004 0.030] |  |
| agegroup1 | |  |  |  | 0.008 | ** |
|  |  |  |  |  | [0.003 0.014] |  |
| agegroup3 | |  |  |  | 0.005 | *** |
|  |  |  |  |  | [0.002 0.007] |  |
| female |  |  |  |  | 0.001 |  |
|  |  |  |  |  | [-0.002 0.004] |  |
| partnerinhh== 3.0000 | | |  |  | 0.003 |  |
|  |  |  |  |  | [-0.004 0.009] |  |
| bieduc |  |  |  |  | 0.030 | *** |
|  |  |  |  |  | [0.018 0.042] |  |
| employment2 | |  |  |  | -0.008 | * |
|  |  |  |  |  | [-0.016 -0.000] |  |
| employment3 | |  |  |  | 0.017 | * |
|  |  |  |  |  | [0.004 0.031] |  |
| employment4 | |  |  |  | 0.004 |  |
|  |  |  |  |  | [-0.002 0.011] |  |
| RECODE of hin_adjlog (hin_adjlog) | | | |  | 0.000 |  |
|  |  |  |  |  | [-0.003 0.002] |  |
| housing==not tenant | |  |  |  | 0.002 |  |
|  |  |  |  |  | [-0.002 0.006] |  |
| EUROD | -0.079 | ** | -0.049 |  | -0.068 | * |
|  | [-0.137 -0.022] |  | [-0.108 0.010] |  | [-0.127 -0.008] |  |
| ADL |  |  | -0.005 |  | -0.004 |  |
|  |  |  | [-0.022 0.011] |  | [-0.019 0.011] |  |
| chron |  |  | -0.011 |  | 0.003 |  |
|  |  |  | [-0.086 0.064] |  | [-0.074 0.080] |  |
| agegroup1 | |  |  |  | -0.105 | ** |
|  |  |  |  |  | [-0.184 -0.026] |  |
| agegroup3 | |  |  |  | 0.001 |  |
|  |  |  |  |  | [-0.012 0.014] |  |
| female |  |  |  |  | 0.098 | * |
|  |  |  |  |  | [0.023 0.173] |  |
| partnerinhh== 3.0000 | | |  |  | -0.007 |  |
|  |  |  |  |  | [-0.053 0.040] |  |
| bieduc |  |  |  |  | 0.008 |  |
|  |  |  |  |  | [-0.011 0.027] |  |
| employment2 | |  |  |  | -0.003 |  |
|  |  |  |  |  | [-0.025 0.019] |  |
| employment3 | |  |  |  | -0.044 |  |
|  |  |  |  |  | [-0.091 0.003] |  |
| employment4 | |  |  |  | -0.086 | * |
|  |  |  |  |  | [-0.161 -0.011] |  |
| RECODE of hin_adjlog (hin_adjlog) | | | |  | 0.067 |  |
|  |  |  |  |  | [-0.274 0.407] |  |
| housing==not tenant | |  |  |  | 0.009 |  |
|  |  |  |  |  | [-0.014 0.032] |  |
| Intercept | 0.116 | * | 0.099 |  | 0.103 |  |
|  | [0.026 0.206] |  | [-0.007 0.206] |  | [-0.318 0.524] |  |
| N | 68404 |  | 68404 |  | 68404 |  |
| *** p<.001, ** p<.01, * p<.05 | | |  |  |  |  |

### Latvia

| Oaxaca decomposition | | |  |  |  |  |
| --- | --- | --- | --- | --- | --- | --- |
|  | srh | | srh | | srh | |
| group_1 | 3.726 | *** | 3.726 | *** | 3.726 | *** |
|  | [3.687 3.765] |  | [3.687 3.765] |  | [3.687 3.765] |  |
| group_2 | 3.103 | *** | 3.103 | *** | 3.103 | *** |
|  | [3.062 3.144] |  | [3.062 3.144] |  | [3.062 3.144] |  |
| difference | 0.623 | *** | 0.623 | *** | 0.623 | *** |
|  | [0.566 0.680] |  | [0.567 0.679] |  | [0.567 0.679] |  |
| explained | -0.056 | *** | 0.015 |  | 0.001 |  |
|  | [-0.084 -0.029] |  | [-0.022 0.052] |  | [-0.048 0.050] |  |
| unexplained | 0.679 | *** | 0.608 | *** | 0.622 | *** |
|  | [0.628 0.731] |  | [0.561 0.655] |  | [0.572 0.672] |  |
| EUROD | -0.056 | *** | -0.042 | *** | -0.040 | *** |
|  | [-0.084 -0.029] |  | [-0.063 -0.021] |  | [-0.060 -0.020] |  |
| ADL |  |  | -0.015 | ** | -0.013 | ** |
|  |  |  | [-0.026 -0.005] |  | [-0.022 -0.004] |  |
| chron |  |  | 0.072 | *** | 0.061 | *** |
|  |  |  | [0.051 0.092] |  | [0.042 0.079] |  |
| agegroup1 | |  |  |  | 0.016 | * |
|  |  |  |  |  | [0.004 0.029] |  |
| agegroup3 | |  |  |  | 0.008 | *** |
|  |  |  |  |  | [0.004 0.011] |  |
| female |  |  |  |  | -0.004 |  |
|  |  |  |  |  | [-0.008 0.000] |  |
| partnerinhh== 3.0000 | | |  |  | 0.001 |  |
|  |  |  |  |  | [-0.002 0.005] |  |
| bieduc |  |  |  |  | -0.051 | *** |
|  |  |  |  |  | [-0.070 -0.031] |  |
| employment2 | |  |  |  | -0.006 |  |
|  |  |  |  |  | [-0.012 0.001] |  |
| employment3 | |  |  |  | -0.014 | * |
|  |  |  |  |  | [-0.026 -0.003] |  |
| employment4 | |  |  |  | 0.044 | *** |
|  |  |  |  |  | [0.024 0.064] |  |
| RECODE of hin_adjlog (hin_adjlog) | | | |  | 0.002 |  |
|  |  |  |  |  | [-0.007 0.010] |  |
| housing==not tenant | |  |  |  | -0.003 |  |
|  |  |  |  |  | [-0.009 0.002] |  |
| EUROD | -0.067 | ** | -0.042 |  | -0.071 | ** |
|  | [-0.114 -0.021] |  | [-0.091 0.007] |  | [-0.119 -0.023] |  |
| ADL |  |  | -0.023 | *** | -0.021 | *** |
|  |  |  | [-0.035 -0.010] |  | [-0.033 -0.008] |  |
| chron |  |  | -0.051 |  | -0.054 |  |
|  |  |  | [-0.123 0.022] |  | [-0.125 0.018] |  |
| agegroup1 | |  |  |  | 0.042 |  |
|  |  |  |  |  | [-0.017 0.102] |  |
| agegroup3 | |  |  |  | 0.010 |  |
|  |  |  |  |  | [-0.006 0.025] |  |
| female |  |  |  |  | 0.047 |  |
|  |  |  |  |  | [-0.016 0.109] |  |
| partnerinhh== 3.0000 | | |  |  | 0.000 |  |
|  |  |  |  |  | [-0.040 0.040] |  |
| bieduc |  |  |  |  | 0.077 | ** |
|  |  |  |  |  | [0.028 0.125] |  |
| employment2 | |  |  |  | 0.015 |  |
|  |  |  |  |  | [-0.001 0.031] |  |
| employment3 | |  |  |  | -0.002 |  |
|  |  |  |  |  | [-0.010 0.005] |  |
| employment4 | |  |  |  | 0.076 |  |
|  |  |  |  |  | [-0.006 0.159] |  |
| RECODE of hin_adjlog (hin_adjlog) | | | |  | -0.136 |  |
|  |  |  |  |  | [-0.466 0.195] |  |
| housing==not tenant | |  |  |  | 0.017 |  |
|  |  |  |  |  | [-0.009 0.043] |  |
| Intercept | 0.746 | *** | 0.724 | *** | 0.622 | ** |
|  | [0.675 0.818] |  | [0.634 0.813] |  | [0.224 1.020] |  |
| N | 66808 |  | 66808 |  | 66808 |  |
| *** p<.001, ** p<.01, * p<.05 | | |  |  |  |  |

### Lithuania

| Oaxaca decomposition | | |  |  |  |  |
| --- | --- | --- | --- | --- | --- | --- |
|  | srh | | srh | | srh | |
| group_1 | 3.603 | *** | 3.603 | *** | 3.603 | *** |
|  | [3.558 3.648] |  | [3.558 3.648] |  | [3.558 3.648] |  |
| group_2 | 3.103 | *** | 3.103 | *** | 3.103 | *** |
|  | [3.062 3.144] |  | [3.062 3.144] |  | [3.062 3.144] |  |
| difference | 0.500 | *** | 0.500 | *** | 0.500 | *** |
|  | [0.438 0.561] |  | [0.439 0.561] |  | [0.439 0.561] |  |
| explained | 0.158 | *** | 0.252 | *** | 0.240 | *** |
|  | [0.125 0.191] |  | [0.211 0.293] |  | [0.190 0.291] |  |
| unexplained | 0.341 | *** | 0.247 | *** | 0.259 | *** |
|  | [0.284 0.399] |  | [0.195 0.299] |  | [0.206 0.313] |  |
| EUROD | 0.158 | *** | 0.118 | *** | 0.113 | *** |
|  | [0.125 0.191] |  | [0.092 0.144] |  | [0.088 0.139] |  |
| ADL |  |  | 0.030 | *** | 0.025 | *** |
|  |  |  | [0.015 0.045] |  | [0.013 0.038] |  |
| chron |  |  | 0.104 | *** | 0.088 | *** |
|  |  |  | [0.081 0.127] |  | [0.068 0.108] |  |
| agegroup1 | |  |  |  | 0.019 | * |
|  |  |  |  |  | [0.004 0.033] |  |
| agegroup3 | |  |  |  | 0.013 | *** |
|  |  |  |  |  | [0.007 0.019] |  |
| female |  |  |  |  | -0.006 | * |
|  |  |  |  |  | [-0.011 -0.000] |  |
| partnerinhh== 3.0000 | | |  |  | 0.000 |  |
|  |  |  |  |  | [-0.001 0.001] |  |
| bieduc |  |  |  |  | -0.051 | *** |
|  |  |  |  |  | [-0.071 -0.032] |  |
| employment2 | |  |  |  | 0.001 |  |
|  |  |  |  |  | [-0.004 0.007] |  |
| employment3 | |  |  |  | -0.017 | * |
|  |  |  |  |  | [-0.030 -0.004] |  |
| employment4 | |  |  |  | 0.051 | *** |
|  |  |  |  |  | [0.028 0.074] |  |
| RECODE of hin_adjlog (hin_adjlog) | | | |  | 0.001 |  |
|  |  |  |  |  | [-0.003 0.004] |  |
| housing==not tenant | |  |  |  | 0.003 |  |
|  |  |  |  |  | [-0.002 0.009] |  |
| EUROD | -0.153 | *** | -0.085 | * | -0.082 | * |
|  | [-0.226 -0.080] |  | [-0.157 -0.013] |  | [-0.156 -0.008] |  |
| ADL |  |  | -0.024 | * | -0.018 |  |
|  |  |  | [-0.045 -0.003] |  | [-0.039 0.003] |  |
| chron |  |  | -0.069 |  | -0.053 |  |
|  |  |  | [-0.159 0.020] |  | [-0.142 0.035] |  |
| agegroup1 | |  |  |  | -0.026 |  |
|  |  |  |  |  | [-0.088 0.036] |  |
| agegroup3 | |  |  |  | -0.012 |  |
|  |  |  |  |  | [-0.035 0.011] |  |
| female |  |  |  |  | -0.023 |  |
|  |  |  |  |  | [-0.092 0.046] |  |
| partnerinhh== 3.0000 | | |  |  | -0.035 |  |
|  |  |  |  |  | [-0.085 0.015] |  |
| bieduc |  |  |  |  | 0.075 | ** |
|  |  |  |  |  | [0.021 0.129] |  |
| employment2 | |  |  |  | -0.007 |  |
|  |  |  |  |  | [-0.029 0.015] |  |
| employment3 | |  |  |  | 0.002 |  |
|  |  |  |  |  | [-0.002 0.006] |  |
| employment4 | |  |  |  | -0.009 |  |
|  |  |  |  |  | [-0.104 0.086] |  |
| RECODE of hin_adjlog (hin_adjlog) | | | |  | -0.353 |  |
|  |  |  |  |  | [-0.792 0.087] |  |
| housing==not tenant | |  |  |  | 0.004 |  |
|  |  |  |  |  | [-0.013 0.022] |  |
| Intercept | 0.494 | *** | 0.426 | *** | 0.795 | ** |
|  | [0.405 0.584] |  | [0.308 0.544] |  | [0.279 1.312] |  |
| N | 66519 |  | 66519 |  | 66519 |  |
| *** p<.001, ** p<.01, * p<.05 | | |  |  |  |  |

### Luxembourg

| Oaxaca decomposition | | |  |  |  |  |
| --- | --- | --- | --- | --- | --- | --- |
|  | srh | | srh | | srh | |
| group_1 | 3.066 | *** | 3.066 | *** | 3.066 | *** |
|  | [2.993 3.140] |  | [2.992 3.141] |  | [2.994 3.138] |  |
| group_2 | 3.103 | *** | 3.103 | *** | 3.103 | *** |
|  | [3.062 3.144] |  | [3.062 3.144] |  | [3.062 3.144] |  |
| difference | -0.037 |  | -0.037 |  | -0.037 |  |
|  | [-0.121 0.047] |  | [-0.122 0.048] |  | [-0.120 0.046] |  |
| explained | 0.035 |  | 0.061 | * | 0.107 | *** |
|  | [-0.003 0.072] |  | [0.013 0.108] |  | [0.043 0.171] |  |
| unexplained | -0.072 |  | -0.098 | * | -0.144 | *** |
|  | [-0.150 0.007] |  | [-0.173 -0.022] |  | [-0.218 -0.069] |  |
| EUROD | 0.035 |  | 0.026 |  | 0.025 |  |
|  | [-0.003 0.072] |  | [-0.002 0.054] |  | [-0.002 0.052] |  |
| ADL |  |  | -0.011 |  | -0.009 |  |
|  |  |  | [-0.027 0.005] |  | [-0.023 0.004] |  |
| chron |  |  | 0.046 | ** | 0.039 | ** |
|  |  |  | [0.018 0.074] |  | [0.015 0.063] |  |
| agegroup1 | |  |  |  | 0.017 | * |
|  |  |  |  |  | [0.004 0.031] |  |
| agegroup3 | |  |  |  | 0.001 |  |
|  |  |  |  |  | [-0.002 0.004] |  |
| female |  |  |  |  | 0.000 |  |
|  |  |  |  |  | [-0.004 0.004] |  |
| partnerinhh== 3.0000 | | |  |  | 0.002 |  |
|  |  |  |  |  | [-0.003 0.007] |  |
| bieduc |  |  |  |  | -0.012 | * |
|  |  |  |  |  | [-0.023 -0.001] |  |
| employment2 | |  |  |  | -0.018 | ** |
|  |  |  |  |  | [-0.030 -0.006] |  |
| employment3 | |  |  |  | 0.006 |  |
|  |  |  |  |  | [-0.001 0.012] |  |
| employment4 | |  |  |  | 0.062 | *** |
|  |  |  |  |  | [0.033 0.090] |  |
| RECODE of hin_adjlog (hin_adjlog) | | | |  | -0.006 |  |
|  |  |  |  |  | [-0.041 0.029] |  |
| housing==not tenant | |  |  |  | 0.001 |  |
|  |  |  |  |  | [-0.002 0.004] |  |
| EUROD | -0.096 | * | -0.037 |  | -0.006 |  |
|  | [-0.185 -0.006] |  | [-0.125 0.050] |  | [-0.089 0.077] |  |
| ADL |  |  | 0.008 |  | 0.008 |  |
|  |  |  | [-0.014 0.029] |  | [-0.009 0.026] |  |
| chron |  |  | -0.191 | *** | -0.182 | *** |
|  |  |  | [-0.295 -0.088] |  | [-0.283 -0.082] |  |
| agegroup1 | |  |  |  | -0.028 |  |
|  |  |  |  |  | [-0.098 0.043] |  |
| agegroup3 | |  |  |  | -0.004 |  |
|  |  |  |  |  | [-0.027 0.018] |  |
| female |  |  |  |  | -0.072 |  |
|  |  |  |  |  | [-0.163 0.019] |  |
| partnerinhh== 3.0000 | | |  |  | 0.028 |  |
|  |  |  |  |  | [-0.029 0.086] |  |
| bieduc |  |  |  |  | -0.017 |  |
|  |  |  |  |  | [-0.067 0.033] |  |
| employment2 | |  |  |  | 0.003 |  |
|  |  |  |  |  | [-0.008 0.014] |  |
| employment3 | |  |  |  | 0.022 |  |
|  |  |  |  |  | [-0.026 0.069] |  |
| employment4 | |  |  |  | 0.013 |  |
|  |  |  |  |  | [-0.164 0.190] |  |
| RECODE of hin_adjlog (hin_adjlog) | | | |  | -0.150 |  |
|  |  |  |  |  | [-1.133 0.832] |  |
| housing==not tenant | |  |  |  | 0.018 |  |
|  |  |  |  |  | [-0.011 0.047] |  |
| Intercept | 0.024 |  | 0.124 |  | 0.223 |  |
|  | [-0.088 0.136] |  | [-0.017 0.265] |  | [-0.815 1.260] |  |
| N | 65942 |  | 65942 |  | 65942 |  |
| *** p<.001, ** p<.01, * p<.05 | | |  |  |  |  |

### Malta

| Oaxaca decomposition | | |  |  |  |  |
| --- | --- | --- | --- | --- | --- | --- |
|  | srh | | srh | | srh | |
| group_1 | 3.318 | *** | 3.318 | *** | 3.318 | *** |
|  | [3.252 3.384] |  | [3.252 3.385] |  | [3.252 3.384] |  |
| group_2 | 3.103 | *** | 3.103 | *** | 3.103 | *** |
|  | [3.062 3.144] |  | [3.062 3.144] |  | [3.062 3.144] |  |
| difference | 0.215 | *** | 0.215 | *** | 0.215 | *** |
|  | [0.137 0.293] |  | [0.137 0.293] |  | [0.137 0.293] |  |
| explained | 0.090 | *** | 0.126 | *** | 0.208 | *** |
|  | [0.055 0.124] |  | [0.084 0.168] |  | [0.148 0.268] |  |
| unexplained | 0.125 | *** | 0.089 | * | 0.007 |  |
|  | [0.051 0.200] |  | [0.018 0.161] |  | [-0.066 0.080] |  |
| EUROD | 0.090 | *** | 0.067 | *** | 0.064 | *** |
|  | [0.055 0.124] |  | [0.041 0.093] |  | [0.039 0.089] |  |
| ADL |  |  | -0.033 | *** | -0.027 | *** |
|  |  |  | [-0.045 -0.021] |  | [-0.038 -0.017] |  |
| chron |  |  | 0.092 | *** | 0.078 | *** |
|  |  |  | [0.066 0.117] |  | [0.055 0.100] |  |
| agegroup1 | |  |  |  | 0.027 | * |
|  |  |  |  |  | [0.006 0.048] |  |
| agegroup3 | |  |  |  | 0.004 | * |
|  |  |  |  |  | [0.000 0.007] |  |
| female |  |  |  |  | 0.000 |  |
|  |  |  |  |  | [-0.004 0.004] |  |
| partnerinhh== 3.0000 | | |  |  | 0.004 |  |
|  |  |  |  |  | [-0.006 0.013] |  |
| bieduc |  |  |  |  | 0.026 | *** |
|  |  |  |  |  | [0.015 0.038] |  |
| employment2 | |  |  |  | -0.020 | ** |
|  |  |  |  |  | [-0.033 -0.006] |  |
| employment3 | |  |  |  | 0.044 | * |
|  |  |  |  |  | [0.009 0.078] |  |
| employment4 | |  |  |  | 0.016 | ** |
|  |  |  |  |  | [0.005 0.027] |  |
| RECODE of hin_adjlog (hin_adjlog) | | | |  | -0.003 |  |
|  |  |  |  |  | [-0.017 0.012] |  |
| housing==not tenant | |  |  |  | -0.004 |  |
|  |  |  |  |  | [-0.012 0.003] |  |
| EUROD | -0.173 | *** | -0.062 |  | -0.062 |  |
|  | [-0.271 -0.076] |  | [-0.160 0.035] |  | [-0.162 0.038] |  |
| ADL |  |  | -0.002 |  | -0.005 |  |
|  |  |  | [-0.014 0.009] |  | [-0.017 0.006] |  |
| chron |  |  | -0.158 | ** | -0.109 |  |
|  |  |  | [-0.277 -0.040] |  | [-0.225 0.007] |  |
| agegroup1 | |  |  |  | 0.018 |  |
|  |  |  |  |  | [-0.044 0.080] |  |
| agegroup3 | |  |  |  | 0.014 |  |
|  |  |  |  |  | [-0.011 0.040] |  |
| female |  |  |  |  | 0.027 |  |
|  |  |  |  |  | [-0.091 0.145] |  |
| partnerinhh== 3.0000 | | |  |  | -0.002 |  |
|  |  |  |  |  | [-0.057 0.054] |  |
| bieduc |  |  |  |  | -0.009 |  |
|  |  |  |  |  | [-0.040 0.022] |  |
| employment2 | |  |  |  | 0.008 |  |
|  |  |  |  |  | [-0.000 0.016] |  |
| employment3 | |  |  |  | -0.024 |  |
|  |  |  |  |  | [-0.131 0.082] |  |
| employment4 | |  |  |  | -0.070 |  |
|  |  |  |  |  | [-0.186 0.045] |  |
| RECODE of hin_adjlog (hin_adjlog) | | | |  | -0.173 |  |
|  |  |  |  |  | [-0.573 0.227] |  |
| housing==not tenant | |  |  |  | 0.016 |  |
|  |  |  |  |  | [-0.025 0.057] |  |
| Intercept | 0.299 | *** | 0.312 | *** | 0.379 |  |
|  | [0.178 0.420] |  | [0.148 0.476] |  | [-0.116 0.873] |  |
| N | 65980 |  | 65980 |  | 65980 |  |
| *** p<.001, ** p<.01, * p<.05 | | |  |  |  |  |

### Netherlands

| Oaxaca decomposition | | |  |  |  |  |
| --- | --- | --- | --- | --- | --- | --- |
|  | srh | | srh | | srh | |
| group_1 | 2.934 | *** | 2.934 | *** | 2.934 | *** |
|  | [2.824 3.044] |  | [2.822 3.045] |  | [2.820 3.047] |  |
| group_2 | 3.103 | *** | 3.103 | *** | 3.103 | *** |
|  | [3.062 3.144] |  | [3.062 3.144] |  | [3.062 3.144] |  |
| difference | -0.170 | ** | -0.170 | ** | -0.170 | ** |
|  | [-0.287 -0.052] |  | [-0.289 -0.051] |  | [-0.290 -0.049] |  |
| explained | -0.057 | ** | -0.069 | * | -0.059 |  |
|  | [-0.099 -0.015] |  | [-0.125 -0.013] |  | [-0.129 0.012] |  |
| unexplained | -0.112 | * | -0.100 |  | -0.111 | * |
|  | [-0.217 -0.007] |  | [-0.202 0.002] |  | [-0.212 -0.010] |  |
| EUROD | -0.057 | ** | -0.043 | ** | -0.041 | ** |
|  | [-0.099 -0.015] |  | [-0.074 -0.011] |  | [-0.071 -0.011] |  |
| ADL |  |  | -0.001 |  | -0.001 |  |
|  |  |  | [-0.020 0.018] |  | [-0.016 0.015] |  |
| chron |  |  | -0.026 |  | -0.022 |  |
|  |  |  | [-0.059 0.008] |  | [-0.050 0.007] |  |
| agegroup1 | |  |  |  | 0.013 | * |
|  |  |  |  |  | [0.002 0.024] |  |
| agegroup3 | |  |  |  | 0.003 | * |
|  |  |  |  |  | [0.000 0.005] |  |
| female |  |  |  |  | 0.000 |  |
|  |  |  |  |  | [-0.005 0.005] |  |
| partnerinhh== 3.0000 | | |  |  | 0.002 |  |
|  |  |  |  |  | [-0.003 0.007] |  |
| bieduc |  |  |  |  | -0.017 | ** |
|  |  |  |  |  | [-0.030 -0.004] |  |
| employment2 | |  |  |  | 0.005 |  |
|  |  |  |  |  | [-0.006 0.017] |  |
| employment3 | |  |  |  | -0.010 | * |
|  |  |  |  |  | [-0.019 -0.002] |  |
| employment4 | |  |  |  | 0.021 | ** |
|  |  |  |  |  | [0.006 0.036] |  |
| RECODE of hin_adjlog (hin_adjlog) | | | |  | -0.006 |  |
|  |  |  |  |  | [-0.039 0.027] |  |
| housing==not tenant | |  |  |  | -0.006 |  |
|  |  |  |  |  | [-0.015 0.004] |  |
| EUROD | 0.144 | ** | 0.162 | ** | 0.130 | ** |
|  | [0.036 0.253] |  | [0.065 0.259] |  | [0.035 0.225] |  |
| ADL |  |  | 0.013 |  | 0.012 |  |
|  |  |  | [-0.007 0.032] |  | [-0.006 0.031] |  |
| chron |  |  | -0.086 |  | -0.061 |  |
|  |  |  | [-0.194 0.022] |  | [-0.153 0.032] |  |
| agegroup1 | |  |  |  | 0.005 |  |
|  |  |  |  |  | [-0.095 0.105] |  |
| agegroup3 | |  |  |  | -0.031 | *** |
|  |  |  |  |  | [-0.047 -0.014] |  |
| female |  |  |  |  | 0.009 |  |
|  |  |  |  |  | [-0.107 0.125] |  |
| partnerinhh== 3.0000 | | |  |  | -0.043 |  |
|  |  |  |  |  | [-0.108 0.023] |  |
| bieduc |  |  |  |  | 0.001 |  |
|  |  |  |  |  | [-0.069 0.072] |  |
| employment2 | |  |  |  | 0.024 |  |
|  |  |  |  |  | [-0.015 0.063] |  |
| employment3 | |  |  |  | -0.008 |  |
|  |  |  |  |  | [-0.028 0.011] |  |
| employment4 | |  |  |  | -0.078 |  |
|  |  |  |  |  | [-0.210 0.055] |  |
| RECODE of hin_adjlog (hin_adjlog) | | | |  | -0.653 |  |
|  |  |  |  |  | [-1.388 0.082] |  |
| housing==not tenant | |  |  |  | 0.046 | * |
|  |  |  |  |  | [0.003 0.089] |  |
| Intercept | -0.256 | ** | -0.189 |  | 0.535 |  |
|  | [-0.412 -0.101] |  | [-0.381 0.002] |  | [-0.273 1.343] |  |
| N | 67075 |  | 67075 |  | 67075 |  |
| *** p<.001, ** p<.01, * p<.05 | | |  |  |  |  |

### Poland

| Oaxaca decomposition | | |  |  |  |  |
| --- | --- | --- | --- | --- | --- | --- |
|  | srh | | srh | | srh | |
| group_1 | 3.403 | *** | 3.403 | *** | 3.403 | *** |
|  | [3.371 3.435] |  | [3.370 3.435] |  | [3.369 3.436] |  |
| group_2 | 3.103 | *** | 3.103 | *** | 3.103 | *** |
|  | [3.062 3.144] |  | [3.062 3.144] |  | [3.062 3.144] |  |
| difference | 0.300 | *** | 0.300 | *** | 0.300 | *** |
|  | [0.247 0.352] |  | [0.247 0.352] |  | [0.247 0.352] |  |
| explained | 0.039 | *** | 0.075 | *** | 0.134 | *** |
|  | [0.016 0.062] |  | [0.045 0.106] |  | [0.098 0.169] |  |
| unexplained | 0.260 | *** | 0.224 | *** | 0.166 | *** |
|  | [0.213 0.308] |  | [0.181 0.267] |  | [0.128 0.204] |  |
| EUROD | 0.039 | *** | 0.029 | *** | 0.028 | *** |
|  | [0.016 0.062] |  | [0.012 0.046] |  | [0.011 0.044] |  |
| ADL |  |  | 0.005 |  | 0.004 |  |
|  |  |  | [-0.004 0.014] |  | [-0.004 0.012] |  |
| chron |  |  | 0.041 | *** | 0.035 | *** |
|  |  |  | [0.025 0.058] |  | [0.021 0.049] |  |
| agegroup1 | |  |  |  | 0.014 | ** |
|  |  |  |  |  | [0.004 0.024] |  |
| agegroup3 | |  |  |  | 0.002 | ** |
|  |  |  |  |  | [0.001 0.004] |  |
| female |  |  |  |  | -0.002 |  |
|  |  |  |  |  | [-0.004 0.001] |  |
| partnerinhh== 3.0000 | | |  |  | 0.000 |  |
|  |  |  |  |  | [-0.001 0.001] |  |
| bieduc |  |  |  |  | 0.018 | *** |
|  |  |  |  |  | [0.010 0.026] |  |
| employment2 | |  |  |  | -0.002 |  |
|  |  |  |  |  | [-0.007 0.003] |  |
| employment3 | |  |  |  | -0.011 | * |
|  |  |  |  |  | [-0.019 -0.002] |  |
| employment4 | |  |  |  | 0.049 | *** |
|  |  |  |  |  | [0.028 0.071] |  |
| RECODE of hin_adjlog (hin_adjlog) | | | |  | -0.001 |  |
|  |  |  |  |  | [-0.004 0.003] |  |
| housing==not tenant | |  |  |  | -0.002 |  |
|  |  |  |  |  | [-0.006 0.002] |  |
| EUROD | -0.027 |  | -0.021 |  | -0.037 |  |
|  | [-0.079 0.024] |  | [-0.075 0.033] |  | [-0.091 0.017] |  |
| ADL |  |  | -0.004 |  | -0.004 |  |
|  |  |  | [-0.018 0.009] |  | [-0.018 0.009] |  |
| chron |  |  | -0.038 |  | -0.043 |  |
|  |  |  | [-0.098 0.021] |  | [-0.103 0.017] |  |
| agegroup1 | |  |  |  | -0.077 | ** |
|  |  |  |  |  | [-0.126 -0.027] |  |
| agegroup3 | |  |  |  | -0.006 |  |
|  |  |  |  |  | [-0.016 0.005] |  |
| female |  |  |  |  | -0.008 |  |
|  |  |  |  |  | [-0.062 0.046] |  |
| partnerinhh== 3.0000 | | |  |  | -0.004 |  |
|  |  |  |  |  | [-0.044 0.036] |  |
| bieduc |  |  |  |  | 0.027 | ** |
|  |  |  |  |  | [0.008 0.045] |  |
| employment2 | |  |  |  | 0.023 | * |
|  |  |  |  |  | [0.005 0.041] |  |
| employment3 | |  |  |  | 0.010 |  |
|  |  |  |  |  | [-0.001 0.021] |  |
| employment4 | |  |  |  | -0.007 |  |
|  |  |  |  |  | [-0.083 0.068] |  |
| RECODE of hin_adjlog (hin_adjlog) | | | |  | -0.060 |  |
|  |  |  |  |  | [-0.487 0.367] |  |
| housing==not tenant | |  |  |  | 0.033 | ** |
|  |  |  |  |  | [0.010 0.057] |  |
| Intercept | 0.288 | *** | 0.287 | *** | 0.319 |  |
|  | [0.219 0.356] |  | [0.205 0.370] |  | [-0.162 0.800] |  |
| N | 69684 |  | 69684 |  | 69684 |  |
| *** p<.001, ** p<.01, * p<.05 | | |  |  |  |  |

### Portugal

| Oaxaca decomposition | | |  |  |  |  |
| --- | --- | --- | --- | --- | --- | --- |
|  | srh | | srh | | srh | |
| group_1 | 3.482 | *** | 3.482 | *** | 3.482 | *** |
|  | [3.278 3.685] |  | [3.297 3.666] |  | [3.305 3.658] |  |
| group_2 | 3.103 | *** | 3.103 | *** | 3.103 | *** |
|  | [3.062 3.144] |  | [3.062 3.144] |  | [3.062 3.144] |  |
| difference | 0.378 | *** | 0.378 | *** | 0.378 | *** |
|  | [0.171 0.586] |  | [0.190 0.567] |  | [0.197 0.560] |  |
| explained | 0.166 | *** | 0.278 | ** | 0.255 | ** |
|  | [0.082 0.251] |  | [0.111 0.445] |  | [0.084 0.427] |  |
| unexplained | 0.212 | * | 0.100 |  | 0.123 |  |
|  | [0.016 0.408] |  | [-0.108 0.309] |  | [-0.074 0.320] |  |
| EUROD | 0.166 | *** | 0.124 | *** | 0.119 | *** |
|  | [0.082 0.251] |  | [0.060 0.189] |  | [0.058 0.181] |  |
| ADL |  |  | 0.090 | * | 0.075 | * |
|  |  |  | [0.014 0.166] |  | [0.011 0.139] |  |
| chron |  |  | 0.063 | * | 0.053 | * |
|  |  |  | [0.001 0.126] |  | [0.001 0.106] |  |
| agegroup1 | |  |  |  | -0.014 | * |
|  |  |  |  |  | [-0.025 -0.002] |  |
| agegroup3 | |  |  |  | -0.007 | *** |
|  |  |  |  |  | [-0.011 -0.003] |  |
| female |  |  |  |  | 0.003 |  |
|  |  |  |  |  | [-0.008 0.014] |  |
| partnerinhh== 3.0000 | | |  |  | 0.004 |  |
|  |  |  |  |  | [-0.005 0.013] |  |
| bieduc |  |  |  |  | 0.040 | *** |
|  |  |  |  |  | [0.023 0.056] |  |
| employment2 | |  |  |  | 0.018 |  |
|  |  |  |  |  | [-0.014 0.050] |  |
| employment3 | |  |  |  | -0.008 |  |
|  |  |  |  |  | [-0.016 0.001] |  |
| employment4 | |  |  |  | -0.029 | ** |
|  |  |  |  |  | [-0.051 -0.007] |  |
| RECODE of hin_adjlog (hin_adjlog) | | | |  | 0.000 |  |
|  |  |  |  |  | [-0.001 0.001] |  |
| housing==not tenant | |  |  |  | 0.001 |  |
|  |  |  |  |  | [-0.003 0.005] |  |
| EUROD | -0.094 |  | 0.107 |  | 0.128 |  |
|  | [-0.322 0.134] |  | [-0.111 0.325] |  | [-0.047 0.303] |  |
| ADL |  |  | -0.236 | ** | -0.199 | * |
|  |  |  | [-0.415 -0.057] |  | [-0.371 -0.027] |  |
| chron |  |  | -0.072 |  | -0.128 |  |
|  |  |  | [-0.292 0.148] |  | [-0.327 0.070] |  |
| agegroup1 | |  |  |  | 0.115 |  |
|  |  |  |  |  | [-0.229 0.459] |  |
| agegroup3 | |  |  |  | -0.003 |  |
|  |  |  |  |  | [-0.018 0.012] |  |
| female |  |  |  |  | -0.021 |  |
|  |  |  |  |  | [-0.194 0.151] |  |
| partnerinhh== 3.0000 | | |  |  | -0.104 |  |
|  |  |  |  |  | [-0.218 0.011] |  |
| bieduc |  |  |  |  | 0.005 |  |
|  |  |  |  |  | [-0.026 0.035] |  |
| employment2 | |  |  |  | 0.018 |  |
|  |  |  |  |  | [-0.083 0.119] |  |
| employment3 | |  |  |  | 0.020 |  |
|  |  |  |  |  | [-0.010 0.050] |  |
| employment4 | |  |  |  | 0.105 |  |
|  |  |  |  |  | [-0.020 0.230] |  |
| RECODE of hin_adjlog (hin_adjlog) | | | |  | -0.438 |  |
|  |  |  |  |  | [-1.072 0.196] |  |
| housing==not tenant | |  |  |  | -0.006 |  |
|  |  |  |  |  | [-0.053 0.040] |  |
| Intercept | 0.306 | * | 0.301 |  | 0.632 |  |
|  | [0.015 0.598] |  | [-0.025 0.628] |  | [-0.196 1.460] |  |
| N | 66551 |  | 66551 |  | 66551 |  |
| *** p<.001, ** p<.01, * p<.05 | | |  |  |  |  |

### Romania

| Oaxaca decomposition | | |  |  |  |  |
| --- | --- | --- | --- | --- | --- | --- |
|  | srh | | srh | | srh | |
| group_1 | 3.531 | *** | 3.531 | *** | 3.531 | *** |
|  | [3.473 3.589] |  | [3.474 3.588] |  | [3.474 3.588] |  |
| group_2 | 3.103 | *** | 3.103 | *** | 3.103 | *** |
|  | [3.062 3.144] |  | [3.062 3.144] |  | [3.062 3.144] |  |
| difference | 0.428 | *** | 0.428 | *** | 0.428 | *** |
|  | [0.357 0.499] |  | [0.357 0.498] |  | [0.358 0.498] |  |
| explained | 0.084 | *** | 0.161 | *** | 0.277 | *** |
|  | [0.048 0.120] |  | [0.115 0.207] |  | [0.222 0.332] |  |
| unexplained | 0.344 | *** | 0.267 | *** | 0.151 | *** |
|  | [0.281 0.406] |  | [0.211 0.323] |  | [0.097 0.204] |  |
| EUROD | 0.084 | *** | 0.063 | *** | 0.060 | *** |
|  | [0.048 0.120] |  | [0.036 0.090] |  | [0.034 0.086] |  |
| ADL |  |  | 0.024 | ** | 0.020 | ** |
|  |  |  | [0.007 0.041] |  | [0.006 0.034] |  |
| chron |  |  | 0.074 | *** | 0.063 | *** |
|  |  |  | [0.051 0.096] |  | [0.043 0.082] |  |
| agegroup1 | |  |  |  | 0.022 | ** |
|  |  |  |  |  | [0.007 0.037] |  |
| agegroup3 | |  |  |  | 0.007 | ** |
|  |  |  |  |  | [0.002 0.011] |  |
| female |  |  |  |  | -0.002 |  |
|  |  |  |  |  | [-0.005 0.002] |  |
| partnerinhh== 3.0000 | | |  |  | 0.002 |  |
|  |  |  |  |  | [-0.003 0.006] |  |
| bieduc |  |  |  |  | 0.031 | *** |
|  |  |  |  |  | [0.019 0.043] |  |
| employment2 | |  |  |  | -0.017 | ** |
|  |  |  |  |  | [-0.028 -0.005] |  |
| employment3 | |  |  |  | 0.000 |  |
|  |  |  |  |  | [-0.004 0.004] |  |
| employment4 | |  |  |  | 0.079 | *** |
|  |  |  |  |  | [0.044 0.114] |  |
| RECODE of hin_adjlog (hin_adjlog) | | | |  | 0.002 |  |
|  |  |  |  |  | [-0.011 0.016] |  |
| housing==not tenant | |  |  |  | 0.010 |  |
|  |  |  |  |  | [-0.005 0.026] |  |
| EUROD | -0.012 |  | 0.013 |  | -0.003 |  |
|  | [-0.077 0.053] |  | [-0.054 0.081] |  | [-0.071 0.065] |  |
| ADL |  |  | 0.004 |  | 0.001 |  |
|  |  |  | [-0.020 0.028] |  | [-0.022 0.025] |  |
| chron |  |  | -0.162 | *** | -0.112 | ** |
|  |  |  | [-0.247 -0.078] |  | [-0.196 -0.028] |  |
| agegroup1 | |  |  |  | -0.033 |  |
|  |  |  |  |  | [-0.091 0.026] |  |
| agegroup3 | |  |  |  | 0.015 |  |
|  |  |  |  |  | [-0.009 0.039] |  |
| female |  |  |  |  | 0.020 |  |
|  |  |  |  |  | [-0.052 0.092] |  |
| partnerinhh== 3.0000 | | |  |  | 0.021 |  |
|  |  |  |  |  | [-0.027 0.069] |  |
| bieduc |  |  |  |  | -0.001 |  |
|  |  |  |  |  | [-0.018 0.017] |  |
| employment2 | |  |  |  | 0.004 |  |
|  |  |  |  |  | [-0.005 0.012] |  |
| employment3 | |  |  |  | -0.013 |  |
|  |  |  |  |  | [-0.039 0.012] |  |
| employment4 | |  |  |  | -0.140 | * |
|  |  |  |  |  | [-0.268 -0.011] |  |
| RECODE of hin_adjlog (hin_adjlog) | | | |  | 0.130 |  |
|  |  |  |  |  | [-0.198 0.458] |  |
| housing==not tenant | |  |  |  | 0.001 |  |
|  |  |  |  |  | [-0.001 0.003] |  |
| Intercept | 0.356 | *** | 0.412 | *** | 0.260 |  |
|  | [0.274 0.438] |  | [0.304 0.520] |  | [-0.157 0.677] |  |
| N | 66592 |  | 66592 |  | 66592 |  |
| *** p<.001, ** p<.01, * p<.05 | | |  |  |  |  |

### Slovakia

| Oaxaca decomposition | | |  |  |  |  |
| --- | --- | --- | --- | --- | --- | --- |
|  | srh | | srh | | srh | |
| group_1 | 2.889 | *** | 2.889 | *** | 2.889 | *** |
|  | [2.825 2.952] |  | [2.826 2.951] |  | [2.826 2.951] |  |
| group_2 | 3.103 | *** | 3.103 | *** | 3.103 | *** |
|  | [3.062 3.144] |  | [3.062 3.144] |  | [3.062 3.144] |  |
| difference | -0.215 | *** | -0.215 | *** | -0.215 | *** |
|  | [-0.290 -0.139] |  | [-0.289 -0.140] |  | [-0.289 -0.140] |  |
| explained | -0.079 | *** | -0.086 | *** | 0.005 |  |
|  | [-0.112 -0.047] |  | [-0.129 -0.043] |  | [-0.045 0.055] |  |
| unexplained | -0.135 | *** | -0.129 | *** | -0.220 | *** |
|  | [-0.201 -0.069] |  | [-0.188 -0.069] |  | [-0.277 -0.162] |  |
| EUROD | -0.079 | *** | -0.059 | *** | -0.057 | *** |
|  | [-0.112 -0.047] |  | [-0.084 -0.034] |  | [-0.080 -0.033] |  |
| ADL |  |  | -0.023 | *** | -0.020 | *** |
|  |  |  | [-0.035 -0.012] |  | [-0.030 -0.010] |  |
| chron |  |  | -0.003 |  | -0.003 |  |
|  |  |  | [-0.026 0.019] |  | [-0.022 0.016] |  |
| agegroup1 | |  |  |  | 0.009 | * |
|  |  |  |  |  | [0.001 0.017] |  |
| agegroup3 | |  |  |  | -0.003 | * |
|  |  |  |  |  | [-0.005 -0.000] |  |
| female |  |  |  |  | 0.000 |  |
|  |  |  |  |  | [-0.003 0.004] |  |
| partnerinhh== 3.0000 | | |  |  | 0.002 |  |
|  |  |  |  |  | [-0.003 0.007] |  |
| bieduc |  |  |  |  | 0.041 | *** |
|  |  |  |  |  | [0.026 0.057] |  |
| employment2 | |  |  |  | -0.015 | ** |
|  |  |  |  |  | [-0.026 -0.004] |  |
| employment3 | |  |  |  | -0.017 | * |
|  |  |  |  |  | [-0.031 -0.004] |  |
| employment4 | |  |  |  | 0.058 | *** |
|  |  |  |  |  | [0.032 0.084] |  |
| RECODE of hin_adjlog (hin_adjlog) | | | |  | 0.000 |  |
|  |  |  |  |  | [-0.001 0.002] |  |
| housing==not tenant | |  |  |  | 0.008 |  |
|  |  |  |  |  | [-0.005 0.021] |  |
| EUROD | 0.101 | ** | 0.053 |  | 0.039 |  |
|  | [0.039 0.163] |  | [-0.007 0.114] |  | [-0.020 0.098] |  |
| ADL |  |  | -0.007 |  | -0.008 |  |
|  |  |  | [-0.019 0.006] |  | [-0.020 0.004] |  |
| chron |  |  | 0.063 |  | 0.060 |  |
|  |  |  | [-0.006 0.131] |  | [-0.013 0.134] |  |
| agegroup1 | |  |  |  | 0.073 |  |
|  |  |  |  |  | [-0.004 0.150] |  |
| agegroup3 | |  |  |  | 0.021 | * |
|  |  |  |  |  | [0.005 0.036] |  |
| female |  |  |  |  | 0.034 |  |
|  |  |  |  |  | [-0.034 0.102] |  |
| partnerinhh== 3.0000 | | |  |  | -0.008 |  |
|  |  |  |  |  | [-0.058 0.042] |  |
| bieduc |  |  |  |  | 0.017 | * |
|  |  |  |  |  | [0.002 0.031] |  |
| employment2 | |  |  |  | 0.011 | * |
|  |  |  |  |  | [0.001 0.022] |  |
| employment3 | |  |  |  | 0.001 |  |
|  |  |  |  |  | [-0.004 0.006] |  |
| employment4 | |  |  |  | 0.072 |  |
|  |  |  |  |  | [-0.035 0.180] |  |
| RECODE of hin_adjlog (hin_adjlog) | | | |  | -0.445 | * |
|  |  |  |  |  | [-0.822 -0.068] |  |
| housing==not tenant | |  |  |  | 0.007 |  |
|  |  |  |  |  | [-0.002 0.016] |  |
| Intercept | -0.236 | *** | -0.238 | *** | -0.094 |  |
|  | [-0.329 -0.144] |  | [-0.346 -0.130] |  | [-0.547 0.358] |  |
| N | 66196 |  | 66196 |  | 66196 |  |
| *** p<.001, ** p<.01, * p<.05 | | |  |  |  |  |

### Slovenia

| Oaxaca decomposition | | |  |  |  |  |
| --- | --- | --- | --- | --- | --- | --- |
|  | srh | | srh | | srh | |
| group_1 | 3.094 | *** | 3.094 | *** | 3.094 | *** |
|  | [3.061 3.127] |  | [3.061 3.126] |  | [3.061 3.127] |  |
| group_2 | 3.103 | *** | 3.103 | *** | 3.103 | *** |
|  | [3.062 3.144] |  | [3.062 3.144] |  | [3.062 3.144] |  |
| difference | -0.010 |  | -0.010 |  | -0.010 |  |
|  | [-0.062 0.043] |  | [-0.062 0.043] |  | [-0.062 0.043] |  |
| explained | -0.055 | *** | 0.017 |  | 0.074 | *** |
|  | [-0.077 -0.034] |  | [-0.012 0.046] |  | [0.038 0.110] |  |
| unexplained | 0.046 |  | -0.027 |  | -0.084 | *** |
|  | [-0.003 0.095] |  | [-0.071 0.017] |  | [-0.121 -0.047] |  |
| EUROD | -0.055 | *** | -0.041 | *** | -0.040 | *** |
|  | [-0.077 -0.034] |  | [-0.058 -0.025] |  | [-0.055 -0.024] |  |
| ADL |  |  | 0.003 |  | 0.002 |  |
|  |  |  | [-0.006 0.012] |  | [-0.005 0.010] |  |
| chron |  |  | 0.056 | *** | 0.047 | *** |
|  |  |  | [0.039 0.072] |  | [0.033 0.061] |  |
| agegroup1 | |  |  |  | 0.018 | * |
|  |  |  |  |  | [0.004 0.031] |  |
| agegroup3 | |  |  |  | 0.009 | *** |
|  |  |  |  |  | [0.005 0.012] |  |
| female |  |  |  |  | -0.003 |  |
|  |  |  |  |  | [-0.007 0.000] |  |
| partnerinhh== 3.0000 | | |  |  | 0.001 |  |
|  |  |  |  |  | [-0.002 0.004] |  |
| bieduc |  |  |  |  | 0.004 |  |
|  |  |  |  |  | [-0.001 0.009] |  |
| employment2 | |  |  |  | -0.011 | ** |
|  |  |  |  |  | [-0.019 -0.003] |  |
| employment3 | |  |  |  | -0.012 | * |
|  |  |  |  |  | [-0.021 -0.002] |  |
| employment4 | |  |  |  | 0.060 | *** |
|  |  |  |  |  | [0.034 0.086] |  |
| RECODE of hin_adjlog (hin_adjlog) | | | |  | -0.002 |  |
|  |  |  |  |  | [-0.013 0.009] |  |
| housing==not tenant | |  |  |  | 0.001 |  |
|  |  |  |  |  | [-0.001 0.003] |  |
| EUROD | -0.032 |  | -0.014 |  | -0.036 |  |
|  | [-0.079 0.014] |  | [-0.062 0.033] |  | [-0.083 0.011] |  |
| ADL |  |  | 0.001 |  | 0.002 |  |
|  |  |  | [-0.013 0.015] |  | [-0.011 0.015] |  |
| chron |  |  | -0.015 |  | -0.041 |  |
|  |  |  | [-0.079 0.049] |  | [-0.105 0.023] |  |
| agegroup1 | |  |  |  | -0.041 |  |
|  |  |  |  |  | [-0.090 0.007] |  |
| agegroup3 | |  |  |  | 0.004 |  |
|  |  |  |  |  | [-0.012 0.019] |  |
| female |  |  |  |  | 0.038 |  |
|  |  |  |  |  | [-0.019 0.096] |  |
| partnerinhh== 3.0000 | | |  |  | 0.002 |  |
|  |  |  |  |  | [-0.039 0.042] |  |
| bieduc |  |  |  |  | 0.019 |  |
|  |  |  |  |  | [-0.006 0.044] |  |
| employment2 | |  |  |  | 0.013 | * |
|  |  |  |  |  | [0.002 0.025] |  |
| employment3 | |  |  |  | 0.008 |  |
|  |  |  |  |  | [-0.002 0.017] |  |
| employment4 | |  |  |  | 0.039 |  |
|  |  |  |  |  | [-0.048 0.126] |  |
| RECODE of hin_adjlog (hin_adjlog) | | | |  | -0.412 | * |
|  |  |  |  |  | [-0.797 -0.028] |  |
| housing==not tenant | |  |  |  | 0.019 |  |
|  |  |  |  |  | [-0.001 0.038] |  |
| Intercept | 0.078 | * | 0.002 |  | 0.303 |  |
|  | [0.010 0.147] |  | [-0.084 0.087] |  | [-0.133 0.739] |  |
| N | 69348 |  | 69348 |  | 69348 |  |
| *** p<.001, ** p<.01, * p<.05 | | |  |  |  |  |

### Spain

| Oaxaca decomposition | | |  |  |  |  |
| --- | --- | --- | --- | --- | --- | --- |
|  | srh | | srh | | srh | |
| group_1 | 2.910 | *** | 2.910 | *** | 2.910 | *** |
|  | [2.815 3.004] |  | [2.817 3.002] |  | [2.818 3.001] |  |
| group_2 | 3.103 | *** | 3.103 | *** | 3.103 | *** |
|  | [3.062 3.144] |  | [3.062 3.144] |  | [3.062 3.144] |  |
| difference | -0.194 | *** | -0.194 | *** | -0.194 | *** |
|  | [-0.296 -0.091] |  | [-0.295 -0.093] |  | [-0.294 -0.093] |  |
| explained | -0.030 |  | -0.109 | *** | -0.180 | *** |
|  | [-0.072 0.011] |  | [-0.161 -0.056] |  | [-0.236 -0.123] |  |
| unexplained | -0.163 | *** | -0.085 |  | -0.014 |  |
|  | [-0.258 -0.069] |  | [-0.173 0.003] |  | [-0.105 0.077] |  |
| EUROD | -0.030 |  | -0.023 |  | -0.022 |  |
|  | [-0.072 0.011] |  | [-0.053 0.008] |  | [-0.052 0.008] |  |
| ADL |  |  | -0.015 | * | -0.013 | * |
|  |  |  | [-0.029 -0.001] |  | [-0.025 -0.001] |  |
| chron |  |  | -0.071 | *** | -0.061 | *** |
|  |  |  | [-0.101 -0.041] |  | [-0.087 -0.035] |  |
| agegroup1 | |  |  |  | -0.018 | * |
|  |  |  |  |  | [-0.033 -0.003] |  |
| agegroup3 | |  |  |  | -0.007 | *** |
|  |  |  |  |  | [-0.010 -0.004] |  |
| female |  |  |  |  | 0.000 |  |
|  |  |  |  |  | [-0.005 0.005] |  |
| partnerinhh== 3.0000 | | |  |  | -0.002 |  |
|  |  |  |  |  | [-0.008 0.004] |  |
| bieduc |  |  |  |  | -0.012 | * |
|  |  |  |  |  | [-0.023 -0.001] |  |
| employment2 | |  |  |  | 0.013 | * |
|  |  |  |  |  | [0.001 0.024] |  |
| employment3 | |  |  |  | 0.004 |  |
|  |  |  |  |  | [-0.002 0.009] |  |
| employment4 | |  |  |  | -0.061 | *** |
|  |  |  |  |  | [-0.087 -0.036] |  |
| RECODE of hin_adjlog (hin_adjlog) | | | |  | 0.000 |  |
|  |  |  |  |  | [-0.006 0.007] |  |
| housing==not tenant | |  |  |  | -0.001 |  |
|  |  |  |  |  | [-0.004 0.002] |  |
| EUROD | -0.024 |  | -0.033 |  | -0.016 |  |
|  | [-0.111 0.064] |  | [-0.129 0.063] |  | [-0.112 0.081] |  |
| ADL |  |  | 0.009 |  | 0.010 |  |
|  |  |  | [-0.008 0.027] |  | [-0.006 0.026] |  |
| chron |  |  | 0.007 |  | 0.024 |  |
|  |  |  | [-0.079 0.094] |  | [-0.063 0.111] |  |
| agegroup1 | |  |  |  | 0.121 |  |
|  |  |  |  |  | [-0.069 0.311] |  |
| agegroup3 | |  |  |  | -0.004 |  |
|  |  |  |  |  | [-0.010 0.001] |  |
| female |  |  |  |  | -0.055 |  |
|  |  |  |  |  | [-0.164 0.053] |  |
| partnerinhh== 3.0000 | | |  |  | 0.008 |  |
|  |  |  |  |  | [-0.081 0.096] |  |
| bieduc |  |  |  |  | 0.007 |  |
|  |  |  |  |  | [-0.052 0.065] |  |
| employment2 | |  |  |  | -0.007 |  |
|  |  |  |  |  | [-0.047 0.033] |  |
| employment3 | |  |  |  | 0.010 |  |
|  |  |  |  |  | [-0.028 0.048] |  |
| employment4 | |  |  |  | 0.001 |  |
|  |  |  |  |  | [-0.039 0.042] |  |
| RECODE of hin_adjlog (hin_adjlog) | | | |  | 0.128 |  |
|  |  |  |  |  | [-0.322 0.578] |  |
| housing==not tenant | |  |  |  | -0.005 |  |
|  |  |  |  |  | [-0.049 0.039] |  |
| Intercept | -0.140 | * | -0.068 |  | -0.236 |  |
|  | [-0.269 -0.011] |  | [-0.205 0.068] |  | [-0.840 0.368] |  |
| N | 67066 |  | 67066 |  | 67066 |  |
| *** p<.001, ** p<.01, * p<.05 | | |  |  |  |  |

### Sweden

| Oaxaca decomposition | | |  |  |  |  |
| --- | --- | --- | --- | --- | --- | --- |
|  | srh | | srh | | srh | |
| group_1 | 2.732 | *** | 2.732 | *** | 2.732 | *** |
|  | [2.675 2.789] |  | [2.676 2.788] |  | [2.675 2.789] |  |
| group_2 | 3.103 | *** | 3.103 | *** | 3.103 | *** |
|  | [3.062 3.144] |  | [3.062 3.144] |  | [3.062 3.144] |  |
| difference | -0.371 | *** | -0.371 | *** | -0.371 | *** |
|  | [-0.442 -0.301] |  | [-0.441 -0.302] |  | [-0.441 -0.301] |  |
| explained | -0.039 | ** | -0.022 |  | -0.051 |  |
|  | [-0.064 -0.014] |  | [-0.055 0.011] |  | [-0.108 0.007] |  |
| unexplained | -0.332 | *** | -0.349 | *** | -0.320 | *** |
|  | [-0.398 -0.267] |  | [-0.408 -0.290] |  | [-0.387 -0.254] |  |
| EUROD | -0.039 | ** | -0.029 | ** | -0.028 | ** |
|  | [-0.064 -0.014] |  | [-0.048 -0.010] |  | [-0.046 -0.010] |  |
| ADL |  |  | -0.002 |  | -0.002 |  |
|  |  |  | [-0.012 0.008] |  | [-0.010 0.007] |  |
| chron |  |  | 0.009 |  | 0.007 |  |
|  |  |  | [-0.011 0.028] |  | [-0.009 0.024] |  |
| agegroup1 | |  |  |  | 0.025 | * |
|  |  |  |  |  | [0.006 0.044] |  |
| agegroup3 | |  |  |  | 0.012 | *** |
|  |  |  |  |  | [0.006 0.017] |  |
| female |  |  |  |  | 0.002 |  |
|  |  |  |  |  | [-0.001 0.006] |  |
| partnerinhh== 3.0000 | | |  |  | 0.000 |  |
|  |  |  |  |  | [-0.001 0.001] |  |
| bieduc |  |  |  |  | -0.062 | *** |
|  |  |  |  |  | [-0.085 -0.039] |  |
| employment2 | |  |  |  | -0.018 | ** |
|  |  |  |  |  | [-0.030 -0.006] |  |
| employment3 | |  |  |  | -0.018 | * |
|  |  |  |  |  | [-0.032 -0.004] |  |
| employment4 | |  |  |  | 0.054 | *** |
|  |  |  |  |  | [0.030 0.078] |  |
| RECODE of hin_adjlog (hin_adjlog) | | | |  | -0.005 |  |
|  |  |  |  |  | [-0.035 0.024] |  |
| housing==not tenant | |  |  |  | -0.018 |  |
|  |  |  |  |  | [-0.048 0.011] |  |
| EUROD | 0.049 |  | 0.082 | * | 0.073 | * |
|  | [-0.021 0.118] |  | [0.014 0.150] |  | [0.005 0.140] |  |
| ADL |  |  | 0.008 |  | 0.007 |  |
|  |  |  | [-0.008 0.023] |  | [-0.008 0.022] |  |
| chron |  |  | 0.044 |  | 0.020 |  |
|  |  |  | [-0.029 0.117] |  | [-0.055 0.096] |  |
| agegroup1 | |  |  |  | 0.010 |  |
|  |  |  |  |  | [-0.055 0.076] |  |
| agegroup3 | |  |  |  | -0.003 |  |
|  |  |  |  |  | [-0.025 0.018] |  |
| female |  |  |  |  | 0.032 |  |
|  |  |  |  |  | [-0.036 0.099] |  |
| partnerinhh== 3.0000 | | |  |  | 0.000 |  |
|  |  |  |  |  | [-0.057 0.058] |  |
| bieduc |  |  |  |  | 0.047 |  |
|  |  |  |  |  | [-0.019 0.112] |  |
| employment2 | |  |  |  | 0.011 |  |
|  |  |  |  |  | [-0.001 0.023] |  |
| employment3 | |  |  |  | -0.001 |  |
|  |  |  |  |  | [-0.005 0.002] |  |
| employment4 | |  |  |  | -0.006 |  |
|  |  |  |  |  | [-0.127 0.116] |  |
| RECODE of hin_adjlog (hin_adjlog) | | | |  | -0.846 | * |
|  |  |  |  |  | [-1.675 -0.017] |  |
| housing==not tenant | |  |  |  | 0.072 | * |
|  |  |  |  |  | [0.008 0.137] |  |
| Intercept | -0.381 | *** | -0.482 | *** | 0.264 |  |
|  | [-0.475 -0.286] |  | [-0.592 -0.373] |  | [-0.626 1.154] |  |
| N | 67473 |  | 67473 |  | 67473 |  |
| *** p<.001, ** p<.01, * p<.05 | | |  |  |  |  |

### Switzerland

| Oaxaca decomposition | | |  |  |  |  |
| --- | --- | --- | --- | --- | --- | --- |
|  | srh | | srh | | srh | |
| group_1 | 2.741 | *** | 2.741 | *** | 2.741 | *** |
|  | [2.689 2.794] |  | [2.690 2.793] |  | [2.689 2.794] |  |
| group_2 | 3.103 | *** | 3.103 | *** | 3.103 | *** |
|  | [3.062 3.144] |  | [3.062 3.144] |  | [3.062 3.144] |  |
| difference | -0.362 | *** | -0.362 | *** | -0.362 | *** |
|  | [-0.428 -0.295] |  | [-0.428 -0.296] |  | [-0.428 -0.295] |  |
| explained | -0.084 | *** | -0.107 | *** | -0.106 | *** |
|  | [-0.110 -0.058] |  | [-0.142 -0.072] |  | [-0.164 -0.048] |  |
| unexplained | -0.278 | *** | -0.255 | *** | -0.256 | *** |
|  | [-0.340 -0.216] |  | [-0.312 -0.197] |  | [-0.321 -0.190] |  |
| EUROD | -0.084 | *** | -0.062 | *** | -0.060 | *** |
|  | [-0.110 -0.058] |  | [-0.082 -0.042] |  | [-0.079 -0.040] |  |
| ADL |  |  | -0.016 | ** | -0.013 | ** |
|  |  |  | [-0.026 -0.006] |  | [-0.022 -0.004] |  |
| chron |  |  | -0.028 | ** | -0.024 | ** |
|  |  |  | [-0.048 -0.008] |  | [-0.041 -0.007] |  |
| agegroup1 | |  |  |  | 0.018 | * |
|  |  |  |  |  | [0.004 0.032] |  |
| agegroup3 | |  |  |  | 0.009 | *** |
|  |  |  |  |  | [0.005 0.014] |  |
| female |  |  |  |  | 0.003 |  |
|  |  |  |  |  | [-0.001 0.007] |  |
| partnerinhh== 3.0000 | | |  |  | 0.000 |  |
|  |  |  |  |  | [-0.001 0.001] |  |
| bieduc |  |  |  |  | -0.034 | *** |
|  |  |  |  |  | [-0.048 -0.020] |  |
| employment2 | |  |  |  | -0.013 | ** |
|  |  |  |  |  | [-0.022 -0.003] |  |
| employment3 | |  |  |  | -0.010 | * |
|  |  |  |  |  | [-0.019 -0.002] |  |
| employment4 | |  |  |  | 0.040 | *** |
|  |  |  |  |  | [0.022 0.059] |  |
| RECODE of hin_adjlog (hin_adjlog) | | | |  | -0.007 |  |
|  |  |  |  |  | [-0.045 0.032] |  |
| housing==not tenant | |  |  |  | -0.016 |  |
|  |  |  |  |  | [-0.041 0.009] |  |
| EUROD | -0.002 |  | 0.018 |  | -0.001 |  |
|  | [-0.066 0.063] |  | [-0.043 0.079] |  | [-0.064 0.061] |  |
| ADL |  |  | 0.007 |  | 0.002 |  |
|  |  |  | [-0.007 0.021] |  | [-0.011 0.016] |  |
| chron |  |  | -0.045 |  | -0.047 |  |
|  |  |  | [-0.106 0.016] |  | [-0.108 0.014] |  |
| agegroup1 | |  |  |  | -0.005 |  |
|  |  |  |  |  | [-0.067 0.056] |  |
| agegroup3 | |  |  |  | 0.013 |  |
|  |  |  |  |  | [-0.007 0.033] |  |
| female |  |  |  |  | 0.055 |  |
|  |  |  |  |  | [-0.008 0.119] |  |
| partnerinhh== 3.0000 | | |  |  | 0.031 |  |
|  |  |  |  |  | [-0.022 0.084] |  |
| bieduc |  |  |  |  | 0.044 |  |
|  |  |  |  |  | [-0.006 0.093] |  |
| employment2 | |  |  |  | 0.015 | * |
|  |  |  |  |  | [0.000 0.029] |  |
| employment3 | |  |  |  | 0.000 |  |
|  |  |  |  |  | [-0.014 0.014] |  |
| employment4 | |  |  |  | -0.056 |  |
|  |  |  |  |  | [-0.148 0.036] |  |
| RECODE of hin_adjlog (hin_adjlog) | | | |  | -0.630 | * |
|  |  |  |  |  | [-1.151 -0.109] |  |
| housing==not tenant | |  |  |  | 0.077 | ** |
|  |  |  |  |  | [0.022 0.133] |  |
| Intercept | -0.276 | *** | -0.234 | *** | 0.247 |  |
|  | [-0.361 -0.191] |  | [-0.336 -0.133] |  | [-0.317 0.810] |  |
| N | 66962 |  | 66962 |  | 66962 |  |
| *** p<.001, ** p<.01, * p<.05 | | |  |  |  |  |
