# Supplementary material for: Cross-country differences in self-rated health: decomposing the influence of depressive symptoms
Source: Eur J Public Health. 2026 Sep 29;36(5):ckag178. doi: 10.1093/eurpub/ckag178 (PMC13622333; doi:10.1093/eurpub/ckag178)
Supplement: Supplementary file 1 [file ckag178_supplementary_data.zip › ejph-2025-11-om-1002-File025.docx]

# Table S5. Partial correlations for the full population, including 95%CI

| Country | Edge | Coefficient | 95%CI lower | 95%CI upper |
| --- | --- | --- | --- | --- |
| Austria | SRH-EUROD | 0.24 | 0.20 | 0.27 |
| Austria | SRH-COND | 0.24 | 0.16 | 0.30 |
| Austria | SRH-ADL | 0.52 | 0.42 | 0.57 |
| Austria | EUROD-COND | 0.00 | -0.04 | 0.05 |
| Austria | EUROD-ADL | 0.20 | 0.15 | 0.26 |
| Austria | COND-ADL | 0.16 | 0.08 | 0.28 |
| Belgium | SRH-EUROD | 0.34 | 0.31 | 0.38 |
| Belgium | SRH-COND | 0.27 | 0.24 | 0.32 |
| Belgium | SRH-ADL | 0.35 | 0.31 | 0.41 |
| Belgium | EUROD-COND | 0.00 | -0.06 | 0.00 |
| Belgium | EUROD-ADL | 0.18 | 0.13 | 0.21 |
| Belgium | COND-ADL | 0.06 | 0.00 | 0.13 |
| Bulgaria | SRH-EUROD | 0.25 | 0.14 | 0.33 |
| Bulgaria | SRH-COND | 0.29 | -0.22 | 0.44 |
| Bulgaria | SRH-ADL | 0.37 | 0.04 | 0.61 |
| Bulgaria | EUROD-COND | 0.05 | -0.50 | 0.18 |
| Bulgaria | EUROD-ADL | 0.25 | 0.09 | 0.58 |
| Bulgaria | COND-ADL | 0.31 | 0.02 | 0.93 |
| Switzerland | SRH-EUROD | 0.22 | 0.15 | 0.28 |
| Switzerland | SRH-COND | 0.24 | 0.13 | 0.32 |
| Switzerland | SRH-ADL | 0.44 | 0.34 | 0.53 |
| Switzerland | EUROD-COND | 0.00 | -0.07 | 0.03 |
| Switzerland | EUROD-ADL | 0.16 | 0.07 | 0.24 |
| Switzerland | COND-ADL | 0.16 | 0.03 | 0.31 |
| Cyprus | SRH-EUROD | 0.19 | 0.08 | 0.28 |
| Cyprus | SRH-COND | 0.32 | 0.19 | 0.45 |
| Cyprus | SRH-ADL | 0.49 | 0.34 | 0.62 |
| Cyprus | EUROD-COND | 0.08 | 0.00 | 0.21 |
| Cyprus | EUROD-ADL | 0.15 | 0.05 | 0.26 |
| Cyprus | COND-ADL | 0.03 | -0.18 | 0.29 |
| Czech republic | SRH-EUROD | 0.27 | 0.23 | 0.32 |
| Czech republic | SRH-COND | 0.36 | 0.29 | 0.41 |
| Czech republic | SRH-ADL | 0.48 | 0.42 | 0.54 |
| Czech republic | EUROD-COND | -0.06 | -0.12 | 0.00 |
| Czech republic | EUROD-ADL | 0.16 | 0.12 | 0.21 |
| Czech republic | COND-ADL | 0.10 | 0.00 | 0.22 |
| Germany | SRH-EUROD | 0.26 | 0.21 | 0.30 |
| Germany | SRH-COND | 0.32 | 0.28 | 0.37 |
| Germany | SRH-ADL | 0.54 | 0.48 | 0.57 |
| Germany | EUROD-COND | -0.07 | -0.11 | -0.03 |
| Germany | EUROD-ADL | 0.13 | 0.08 | 0.18 |
| Germany | COND-ADL | 0.07 | 0.00 | 0.14 |
| Denmark | SRH-EUROD | 0.33 | 0.29 | 0.39 |
| Denmark | SRH-COND | 0.24 | 0.17 | 0.30 |
| Denmark | SRH-ADL | 0.47 | 0.40 | 0.55 |
| Denmark | EUROD-COND | 0.00 | -0.06 | 0.04 |
| Denmark | EUROD-ADL | 0.09 | 0.01 | 0.14 |
| Denmark | COND-ADL | 0.12 | 0.01 | 0.23 |
| Estonia | SRH-EUROD | 0.25 | 0.22 | 0.29 |
| Estonia | SRH-COND | 0.39 | 0.34 | 0.43 |
| Estonia | SRH-ADL | 0.46 | 0.40 | 0.50 |
| Estonia | EUROD-COND | 0.06 | 0.01 | 0.10 |
| Estonia | EUROD-ADL | 0.18 | 0.13 | 0.23 |
| Estonia | COND-ADL | 0.01 | -0.05 | 0.09 |
| Spain | SRH-EUROD | 0.37 | 0.32 | 0.41 |
| Spain | SRH-COND | 0.27 | 0.20 | 0.33 |
| Spain | SRH-ADL | 0.35 | 0.29 | 0.44 |
| Spain | EUROD-COND | 0.00 | -0.05 | 0.08 |
| Spain | EUROD-ADL | 0.20 | 0.14 | 0.25 |
| Spain | COND-ADL | 0.23 | 0.14 | 0.33 |
| France | SRH-EUROD | 0.32 | 0.28 | 0.35 |
| France | SRH-COND | 0.31 | 0.25 | 0.35 |
| France | SRH-ADL | 0.34 | 0.28 | 0.41 |
| France | EUROD-COND | -0.04 | -0.10 | 0.00 |
| France | EUROD-ADL | 0.11 | 0.06 | 0.18 |
| France | COND-ADL | 0.16 | 0.07 | 0.22 |
| Finland | SRH-EUROD | 0.23 | 0.16 | 0.27 |
| Finland | SRH-COND | 0.38 | 0.30 | 0.44 |
| Finland | SRH-ADL | 0.48 | 0.41 | 0.56 |
| Finland | EUROD-COND | 0.00 | -0.06 | 0.06 |
| Finland | EUROD-ADL | 0.10 | 0.01 | 0.18 |
| Finland | COND-ADL | 0.00 | -0.11 | 0.13 |
| Greece | SRH-EUROD | 0.24 | 0.18 | 0.31 |
| Greece | SRH-COND | 0.51 | 0.44 | 0.56 |
| Greece | SRH-ADL | 0.40 | 0.28 | 0.49 |
| Greece | EUROD-COND | -0.09 | -0.15 | -0.01 |
| Greece | EUROD-ADL | 0.27 | 0.22 | 0.31 |
| Greece | COND-ADL | 0.03 | -0.10 | 0.23 |
| Croatia | SRH-EUROD | 0.27 | 0.23 | 0.30 |
| Croatia | SRH-COND | 0.37 | 0.32 | 0.41 |
| Croatia | SRH-ADL | 0.50 | 0.44 | 0.56 |
| Croatia | EUROD-COND | 0.05 | 0.01 | 0.11 |
| Croatia | EUROD-ADL | 0.17 | 0.12 | 0.22 |
| Croatia | COND-ADL | 0.00 | -0.08 | 0.07 |
| Hungary | SRH-EUROD | 0.36 | 0.31 | 0.41 |
| Hungary | SRH-COND | 0.26 | 0.18 | 0.33 |
| Hungary | SRH-ADL | 0.39 | 0.24 | 0.47 |
| Hungary | EUROD-COND | 0.01 | -0.05 | 0.08 |
| Hungary | EUROD-ADL | 0.11 | 0.02 | 0.20 |
| Hungary | COND-ADL | 0.21 | 0.07 | 0.39 |
| Israel | SRH-EUROD | 0.15 | -0.15 | 0.27 |
| Israel | SRH-COND | 0.31 | -0.55 | 0.50 |
| Israel | SRH-ADL | 0.53 | 0.25 | 0.72 |
| Israel | EUROD-COND | 0.03 | -0.61 | 0.23 |
| Israel | EUROD-ADL | 0.31 | 0.14 | 0.67 |
| Israel | COND-ADL | 0.25 | 0.00 | 0.94 |
| Italy | SRH-EUROD | 0.29 | 0.24 | 0.32 |
| Italy | SRH-COND | 0.38 | 0.33 | 0.41 |
| Italy | SRH-ADL | 0.44 | 0.37 | 0.50 |
| Italy | EUROD-COND | 0.00 | -0.03 | 0.03 |
| Italy | EUROD-ADL | 0.19 | 0.14 | 0.24 |
| Italy | COND-ADL | 0.00 | -0.08 | 0.10 |
| Lithuania | SRH-EUROD | 0.34 | 0.27 | 0.40 |
| Lithuania | SRH-COND | 0.33 | 0.21 | 0.41 |
| Lithuania | SRH-ADL | 0.43 | 0.34 | 0.53 |
| Lithuania | EUROD-COND | -0.05 | -0.13 | 0.03 |
| Lithuania | EUROD-ADL | 0.10 | 0.00 | 0.19 |
| Lithuania | COND-ADL | 0.14 | 0.01 | 0.29 |
| Luxembourg | SRH-EUROD | 0.26 | 0.17 | 0.36 |
| Luxembourg | SRH-COND | 0.13 | -0.07 | 0.27 |
| Luxembourg | SRH-ADL | 0.55 | 0.42 | 0.69 |
| Luxembourg | EUROD-COND | 0.05 | 0.00 | 0.14 |
| Luxembourg | EUROD-ADL | 0.12 | 0.00 | 0.23 |
| Luxembourg | COND-ADL | 0.12 | -0.12 | 0.39 |
| Latvia | SRH-EUROD | 0.30 | 0.20 | 0.37 |
| Latvia | SRH-COND | 0.45 | 0.37 | 0.51 |
| Latvia | SRH-ADL | 0.32 | 0.17 | 0.51 |
| Latvia | EUROD-COND | 0.06 | 0.00 | 0.15 |
| Latvia | EUROD-ADL | 0.27 | 0.17 | 0.33 |
| Latvia | COND-ADL | 0.02 | -0.15 | 0.23 |
| Malta | SRH-EUROD | 0.16 | 0.02 | 0.25 |
| Malta | SRH-COND | 0.22 | 0.04 | 0.35 |
| Malta | SRH-ADL | 0.50 | 0.38 | 0.61 |
| Malta | EUROD-COND | -0.06 | -0.20 | 0.00 |
| Malta | EUROD-ADL | 0.27 | 0.14 | 0.42 |
| Malta | COND-ADL | 0.06 | -0.16 | 0.31 |
| Netherlands | SRH-EUROD | 0.27 | 0.22 | 0.33 |
| Netherlands | SRH-COND | 0.32 | 0.23 | 0.36 |
| Netherlands | SRH-ADL | 0.51 | 0.43 | 0.58 |
| Netherlands | EUROD-COND | 0.00 | -0.06 | 0.02 |
| Netherlands | EUROD-ADL | 0.11 | 0.04 | 0.18 |
| Netherlands | COND-ADL | 0.00 | -0.09 | 0.12 |
| Poland | SRH-EUROD | 0.25 | 0.21 | 0.29 |
| Poland | SRH-COND | 0.34 | 0.28 | 0.39 |
| Poland | SRH-ADL | 0.45 | 0.40 | 0.51 |
| Poland | EUROD-COND | 0.05 | 0.00 | 0.09 |
| Poland | EUROD-ADL | 0.18 | 0.14 | 0.22 |
| Poland | COND-ADL | 0.10 | 0.01 | 0.19 |
| Portugal | SRH-EUROD | 0.28 | 0.22 | 0.33 |
| Portugal | SRH-COND | 0.31 | 0.22 | 0.38 |
| Portugal | SRH-ADL | 0.39 | 0.29 | 0.46 |
| Portugal | EUROD-COND | 0.00 | -0.08 | 0.06 |
| Portugal | EUROD-ADL | 0.22 | 0.16 | 0.28 |
| Portugal | COND-ADL | 0.02 | -0.11 | 0.17 |
| Romania | SRH-EUROD | 0.37 | 0.31 | 0.42 |
| Romania | SRH-COND | 0.31 | 0.24 | 0.40 |
| Romania | SRH-ADL | 0.45 | 0.36 | 0.59 |
| Romania | EUROD-COND | 0.05 | 0.00 | 0.11 |
| Romania | EUROD-ADL | 0.16 | 0.07 | 0.24 |
| Romania | COND-ADL | 0.00 | -0.18 | 0.13 |
| Sweden | SRH-EUROD | 0.29 | 0.22 | 0.34 |
| Sweden | SRH-COND | 0.37 | 0.30 | 0.44 |
| Sweden | SRH-ADL | 0.43 | 0.36 | 0.51 |
| Sweden | EUROD-COND | -0.05 | -0.10 | 0.00 |
| Sweden | EUROD-ADL | 0.11 | 0.05 | 0.17 |
| Sweden | COND-ADL | -0.06 | -0.16 | 0.00 |
| Slovenia | SRH-EUROD | 0.22 | 0.18 | 0.25 |
| Slovenia | SRH-COND | 0.32 | 0.27 | 0.36 |
| Slovenia | SRH-ADL | 0.42 | 0.37 | 0.47 |
| Slovenia | EUROD-COND | 0.00 | -0.05 | 0.02 |
| Slovenia | EUROD-ADL | 0.20 | 0.16 | 0.25 |
| Slovenia | COND-ADL | 0.11 | 0.02 | 0.20 |
| Slovakia | SRH-EUROD | 0.30 | 0.19 | 0.39 |
| Slovakia | SRH-COND | 0.44 | 0.28 | 0.55 |
| Slovakia | SRH-ADL | 0.49 | 0.27 | 0.59 |
| Slovakia | EUROD-COND | 0.11 | 0.00 | 0.22 |
| Slovakia | EUROD-ADL | 0.11 | 0.00 | 0.26 |
| Slovakia | COND-ADL | 0.09 | -0.12 | 0.41 |

Note: Partial correlations between variables estimated using EBICglasso. Values represent regularized partial correlation coefficients. 95% confidence intervals (CI) were approximated using Fisher’s z-transformation.
