# Supplementary material for: Cross-country differences in self-rated health: decomposing the influence of depressive symptoms
Source: Eur J Public Health. 2026 Sep 29;36(5):ckag178. doi: 10.1093/eurpub/ckag178 (PMC13622333; doi:10.1093/eurpub/ckag178)
Supplement: Supplementary file 1 [file ckag178_supplementary_data.zip › ejph-2025-11-om-1002-File028.docx]

# Table S8. Country differences in SRH decomposed into total difference (left), the part explained by depressive symptoms (EUROD-D) levels (centre), and the part explained by differences in the SRH-EURO-D association (right), by gender

| **Country** | **Gender** | **Difference** | 95%CI | | **EUROD explained** | 95%CI | | **EUROD unexplained** | 95%CI | |
| --- | --- | --- | --- | --- | --- | --- | --- | --- | --- | --- |
|  |  |  | Lower | Upper |  | Lower | Upper |  | Lower | Upper |
| Austria | Female | -0.130 | -0.205 | -0.055 | -0.061 | -0.086 | -0.037 | 0.069 | -0.002 | 0.140 |
| Austria | Male | -0.148 | -0.238 | -0.057 | -0.033 | -0.054 | -0.012 | 0.126 | 0.059 | 0.192 |
| Belgium | Female | -0.042 | -0.108 | 0.024 | 0.008 | -0.015 | 0.030 | -0.046 | -0.115 | 0.023 |
| Belgium | Male | -0.123 | -0.200 | -0.045 | 0.016 | -0.003 | 0.034 | 0.099 | 0.039 | 0.160 |
| Bulgaria | Female | 0.296 | 0.189 | 0.403 | -0.012 | -0.054 | 0.029 | -0.095 | -0.198 | 0.007 |
| Bulgaria | Male | 0.107 | -0.035 | 0.250 | -0.015 | -0.052 | 0.022 | 0.082 | -0.017 | 0.181 |
| Croatia | Female | 0.147 | 0.075 | 0.219 | -0.025 | -0.049 | -0.001 | -0.017 | -0.083 | 0.050 |
| Croatia | Male | 0.042 | -0.044 | 0.128 | -0.010 | -0.030 | 0.010 | 0.074 | 0.013 | 0.134 |
| Czech Republic | Female | -0.126 | -0.344 | 0.093 | 0.036 | -0.027 | 0.099 | -0.218 | -0.363 | -0.074 |
| Czech Republic | Male | -0.178 | -0.339 | -0.017 | -0.005 | -0.053 | 0.042 | 0.093 | -0.040 | 0.226 |
| Denmark | Female | -0.462 | -0.548 | -0.376 | -0.079 | -0.106 | -0.053 | 0.175 | 0.096 | 0.254 |
| Denmark | Male | -0.472 | -0.568 | -0.376 | -0.036 | -0.058 | -0.015 | 0.108 | 0.033 | 0.183 |
| Estonia | Female | 0.605 | 0.542 | 0.669 | 0.046 | 0.023 | 0.068 | -0.105 | -0.178 | -0.032 |
| Estonia | Male | 0.625 | 0.544 | 0.705 | 0.078 | 0.053 | 0.103 | -0.074 | -0.155 | 0.007 |
| Finland | Female | 0.009 | -0.083 | 0.101 | -0.037 | -0.064 | -0.011 | -0.071 | -0.175 | 0.032 |
| Finland | Male | 0.084 | -0.031 | 0.198 | 0.002 | -0.022 | 0.026 | 0.082 | -0.002 | 0.167 |
| France | Female | -0.054 | -0.135 | 0.028 | 0.039 | 0.013 | 0.064 | 0.011 | -0.083 | 0.105 |
| France | Male | -0.077 | -0.173 | 0.018 | 0.037 | 0.014 | 0.059 | 0.075 | -0.016 | 0.166 |
| Germany | Female | 0.012 | -0.060 | 0.085 | 0.002 | -0.021 | 0.024 | -0.020 | -0.105 | 0.065 |
| Germany | Male | 0.092 | 0.006 | 0.178 | 0.027 | 0.006 | 0.047 | 0.012 | -0.059 | 0.083 |
| Greece | Female | -0.135 | -0.209 | -0.062 | -0.124 | -0.154 | -0.093 | -0.068 | -0.127 | -0.009 |
| Greece | Male | -0.120 | -0.028 | -0.031 | -0.072 | -0.098 | -0.045 | -0.008 | -0.067 | 0.052 |
| Hungary | Female | -0.049 | -0.139 | 0.042 | -0.104 | -0.138 | -0.071 | 0.012 | -0.056 | 0.080 |
| Hungary | Male | -0.127 | -0.237 | -0.016 | -0.066 | -0.094 | -0.037 | 0.028 | -0.032 | 0.088 |
| Israel | Female | -0.126 | -0.323 | 0.070 | 0.001 | -0.050 | 0.052 | 0.014 | -0.136 | 0.164 |
| Israel | Male | -0.128 | -0.451 | 0.195 | 0.013 | -0.038 | 0.064 | 0.009 | -0.127 | 0.146 |
| Italy | Female | 0.076 | -0.009 | 0.161 | -0.003 | -0.033 | 0.027 | -0.095 | -0.176 | -0.014 |
| Italy | Male | 0.006 | -0.104 | 0.116 | 0.020 | -0.006 | 0.045 | -0.015 | -0.097 | 0.067 |
| Latvia | Female | 0.648 | 0.576 | 0.720 | -0.057 | -0.085 | -0.029 | -0.090 | -0.155 | -0.025 |
| Latvia | Male | 0.579 | 0.490 | 0.669 | -0.028 | -0.053 | -0.002 | -0.045 | -0.110 | 0.020 |
| Lithuania | Female | 0.497 | 0.418 | 0.576 | 0.115 | 0.081 | 0.149 | -0.089 | -0.191 | 0.013 |
| Lithuania | Male | 0.499 | 0.407 | 0.592 | 0.090 | 0.056 | 0.124 | -0.074 | -0.167 | 0.018 |
| Malta | Female | 0.245 | 0.146 | 0.343 | 0.062 | 0.026 | 0.097 | -0.040 | -0.167 | 0.088 |
| Malta | Male | 0.175 | 0.051 | 0.299 | 0.066 | 0.031 | 0.100 | -0.074 | -0.209 | 0.060 |
| Netherlands | Female | -0.125 | -0.298 | 0.048 | -0.048 | -0.090 | -0.006 | 0.132 | -0.001 | 0.266 |
| Netherlands | Male | -0.231 | -0.038 | -0.086 | -0.032 | -0.068 | 0.004 | 0.111 | 0.012 | 0.211 |
| Poland | Female | 0.301 | 0.234 | 0.369 | 0.023 | 0.000 | 0.047 | -0.080 | 0.154 | -0.006 |
| Poland | Male | 0.296 | 0.213 | 0.379 | 0.029 | 0.008 | 0.050 | 0.022 | -0.048 | 0.092 |
| Portugal | Female | 0.323 | 0.081 | 0.565 | 0.145 | 0.066 | 0.225 | -0.011 | -0.258 | 0.236 |
| Portugal | Male | 0.447 | 0.206 | 0.689 | 0.096 | 0.005 | 0.187 | 0.346 | 0.108 | 0.585 |
| Romania | Female | 0.471 | 0.382 | 0.560 | 0.065 | 0.028 | 0.101 | -0.027 | -0.121 | 0.067 |
| Romania | Male | 0.362 | 0.253 | 0.471 | 0.048 | 0.016 | 0.080 | 0.023 | -0.066 | 0.112 |
| Slovakia | Female | -0.200 | -0.302 | -0.099 | -0.083 | -0.119 | -0.048 | 0.039 | -0.042 | 0.121 |
| Slovakia | Male | -0.234 | -0.344 | -0.124 | -0.022 | -0.051 | 0.007 | 0.036 | -0.044 | 0.115 |
| Slovenia | Female | 0.019 | -0.049 | 0.087 | -0.046 | -0.069 | -0.023 | -0.054 | -0.122 | 0.013 |
| Slovenia | Male | -0.057 | -0.138 | 0.025 | -0.039 | -0.059 | -0.020 | -0.018 | -0.075 | 0.039 |
| Spain | Female | -0.237 | -0.366 | -0.107 | -0.017 | -0.063 | 0.028 | 0.023 | -0.114 | 0.160 |
| Spain | Male | -0.136 | -0.293 | 0.021 | -0.026 | -0.059 | 0.008 | -0.068 | -0.182 | 0.046 |
| Sweden | Female | -0.346 | -0.440 | -0.252 | -0.035 | -0.061 | -0.008 | 0.067 | -0.031 | 0.165 |
| Sweden | Male | -0.400 | -0.505 | -0.296 | -0.013 | -0.035 | 0.008 | 0.079 | -0.009 | 0.167 |
| Switzerland | Female | -0.307 | -0.392 | -0.222 | -0.068 | -0.096 | -0.039 | 0.007 | -0.080 | 0.094 |
| Switzerland | Male | -0.424 | -0.528 | -0.321 | -0.042 | -0.065 | -0.019 | -0.012 | -0.096 | 0.073 |
|  |  |  |  |  |  |  |  |  |  |  |

(See figure S5)
