# Supplementary material for: Cross-country differences in self-rated health: decomposing the influence of depressive symptoms
Source: Eur J Public Health. 2026 Sep 29;36(5):ckag178. doi: 10.1093/eurpub/ckag178 (PMC13622333; doi:10.1093/eurpub/ckag178)
Supplement: Supplementary file 1 [file ckag178_supplementary_data.zip › ejph-2025-11-om-1002-File023.docx]

# Table S3. Pseudo R-square comparison across countries (interval regression)

|  | Socio-economic | | |  | + Health/ADL | | |  | +EURO-D | | |
| --- | --- | --- | --- | --- | --- | --- | --- | --- | --- | --- | --- |
| Country | R squared | CI lower | CI upper |  | R squared | CI_lower | CI_upper |  | R_squared | CI_lower | CI_upper |
| Austria | 0.080 | 0.068 | 0.092 |  | 0.125 | 0.112 | 0.140 |  | 0.206 | 0.188 | 0.223 |
| Belgium | 0.091 | 0.078 | 0.106 |  | 0.126 | 0.112 | 0.143 |  | 0.190 | 0.173 | 0.208 |
| Bulgaria | 0.093 | 0.071 | 0.122 |  | 0.153 | 0.123 | 0.180 |  | 0.201 | 0.167 | 0.236 |
| Switzerland | 0.080 | 0.061 | 0.098 |  | 0.121 | 0.100 | 0.141 |  | 0.157 | 0.137 | 0.178 |
| Cyprus | 0.088 | 0.065 | 0.114 |  | 0.130 | 0.102 | 0.161 |  | 0.167 | 0.132 | 0.202 |
| Czech republic | 0.089 | 0.049 | 0.148 |  | 0.186 | 0.142 | 0.241 |  | 0.233 | 0.196 | 0.281 |
| Germany | 0.076 | 0.064 | 0.087 |  | 0.130 | 0.116 | 0.146 |  | 0.182 | 0.166 | 0.198 |
| Denmark | 0.069 | 0.056 | 0.083 |  | 0.104 | 0.090 | 0.120 |  | 0.163 | 0.148 | 0.179 |
| Estonia | 0.109 | 0.098 | 0.121 |  | 0.160 | 0.147 | 0.174 |  | 0.218 | 0.202 | 0.234 |
| Spain | 0.069 | 0.049 | 0.093 |  | 0.124 | 0.097 | 0.155 |  | 0.171 | 0.144 | 0.207 |
| France | 0.064 | 0.052 | 0.078 |  | 0.102 | 0.086 | 0.118 |  | 0.172 | 0.153 | 0.192 |
| Finland | 0.081 | 0.062 | 0.102 |  | 0.131 | 0.109 | 0.153 |  | 0.177 | 0.153 | 0.203 |
| Greece | 0.119 | 0.104 | 0.135 |  | 0.164 | 0.147 | 0.180 |  | 0.217 | 0.199 | 0.238 |
| Croatia | 0.079 | 0.069 | 0.091 |  | 0.124 | 0.113 | 0.138 |  | 0.202 | 0.189 | 0.215 |
| Hungary | 0.084 | 0.066 | 0.104 |  | 0.119 | 0.100 | 0.139 |  | 0.173 | 0.147 | 0.195 |
| Israel | 0.145 | 0.108 | 0.187 |  | 0.217 | 0.178 | 0.264 |  | 0.385 | 0.343 | 0.430 |
| Italy | 0.080 | 0.063 | 0.099 |  | 0.127 | 0.107 | 0.148 |  | 0.185 | 0.162 | 0.208 |
| Lithuania | 0.070 | 0.055 | 0.089 |  | 0.127 | 0.107 | 0.150 |  | 0.212 | 0.186 | 0.238 |
| Luxembourg | 0.066 | 0.044 | 0.092 |  | 0.101 | 0.075 | 0.129 |  | 0.176 | 0.143 | 0.209 |
| Latvia | 0.120 | 0.095 | 0.147 |  | 0.178 | 0.152 | 0.203 |  | 0.230 | 0.202 | 0.261 |
| Malta | 0.088 | 0.064 | 0.113 |  | 0.106 | 0.081 | 0.136 |  | 0.147 | 0.116 | 0.178 |
| Netherlands | 0.097 | 0.073 | 0.132 |  | 0.133 | 0.101 | 0.171 |  | 0.180 | 0.151 | 0.214 |
| Poland | 0.095 | 0.081 | 0.107 |  | 0.147 | 0.133 | 0.163 |  | 0.230 | 0.213 | 0.246 |
| Portugal | 0.090 | 0.053 | 0.135 |  | 0.116 | 0.070 | 0.173 |  | 0.188 | 0.139 | 0.246 |
| Romania | 0.082 | 0.060 | 0.104 |  | 0.131 | 0.107 | 0.155 |  | 0.210 | 0.184 | 0.240 |
| Sweden | 0.076 | 0.062 | 0.091 |  | 0.111 | 0.093 | 0.131 |  | 0.169 | 0.149 | 0.189 |
| Slovenia | 0.092 | 0.081 | 0.104 |  | 0.139 | 0.126 | 0.151 |  | 0.197 | 0.182 | 0.214 |
| Slovakia | 0.093 | 0.071 | 0.115 |  | 0.178 | 0.155 | 0.204 |  | 0.238 | 0.210 | 0.267 |

(See figure S1)
